# CO_2_‐Induced Reverse Lattice Oxygen Spillover on Pt/CeO_2_ Enables Sulfur‐Resistant Dry Reforming of Methane

**DOI:** 10.1002/anie.1664469

**Published:** 2026-05-31

**Authors:** Jun Liu, Jiang Deng, Jiajia Zheng, Mohsen Beladi Mousavi, Chunning Sun, Jin Li, Xin Chen, Yongjie Shen, Haotian Huang, Ming Xie, Emiliano Cortés, Dengsong Zhang

**Affiliations:** ^1^ Innovation Institute of Carbon Neutrality, International Joint Laboratory of Catalytic Chemistry, College of Sciences State Key Laboratory of Materials for Advanced Nuclear Energy, Shanghai University China; ^2^ Nanoinstitute Munich, Faculty of Physics Ludwig‐Maximilians‐Universität (LMU) Munich Germany; ^3^ Inorganic Chemistry and Catalysis Group, Debye Institute for Nanomaterials Science & Institute for Sustainable and Circular Chemistry Utrecht University Utrecht the Netherlands; ^4^ Department of Chemical Engineering University of Bath Bath UK

**Keywords:** biogas upgrading, dry reforming of methane, H_2_S poisoning‐resistance, platinum

## Abstract

Overcoming sulfur poisoning in dry reforming of methane (DRM), which is a critical process for biogas upgrading, is particularly challenging. In this study, we illustrate that a reverse lattice oxygen spillover (RLOS) from CeO_2_ to Pt on the Pt‐O‐Ce interface, induced by CO_2_, can oxidize S into SO_2_, aiding in the removal of S deposits. A low oxygen migration barrier at the Pt–O–Ce interface and Pt's high activity for oxidizing sulfur to SO_2_ make Pt/CeO_2_ uniquely effective at self‐recovering after H_2_S poisoning. Furthermore, the atomically dispersed Pt/CeO_2_ catalyst undergoes reaction driven adaptive restructuring, which amplifies the RLOS effect and enables dynamic S deposition and removal. As a result, the catalysts maintain constant DRM activity for 100 h, even in the presence of H_2_S. This discovery paves the way for designing catalysts that resist sulfur poisoning in H_2_S‐containing streams.

## Graphical abstract

A critical step, dry reforming of methane (DRM) converts biogas into useful syngas. Yet trace H_2_S in biogas rapidly poisons DRM catalysts, limiting practical use. We developed a robust Pt/CeO_2_ catalyst sustaining DRM activity via CO_2_‐induced reverse lattice oxygen spillover in H_2_S‐rich environments. This process continuously oxidizes and removes surface sulfur, maintaining catalytic performance. The breakthrough enables promising fuel production from biogas, accelerating the shift to sustainable energy.

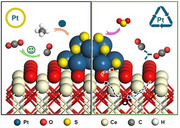

## 1 Introduction

The global push for carbon neutrality, driven by policies such as the European Union's Carbon Border Adjustment Mechanism, is increasing the demand for green energy solutions [1, 2]. Biogas, a sustainable and renewable resource, is a crucial feedstock for producing vital green fuels like methanol and sustainable aviation fuel [3, 4]. A key process for utilizing biogas is the dry reforming of methane (DRM), which converts CH_4_ and CO_2_ into syngas [5, 6, 7, 8]. Unlike methods that merely separate CO_2_, DRM not only maximizes carbon utilization from biogas but also boosts green fuel production and further reduces carbon emissions [9]. However, catalyst poisoning is a major obstacle in DRM [10, 11, 12]. Despite extensive desulfurization efforts, trace amounts of H_2_S (typically less than 15 ppm) may remain in feedstocks, which can still lead to rapid catalyst deactivation [13, 14]. This severely impedes the industrial viability and advancement of green fuel production. When H_2_S encounters catalyst surfaces, it readily bonds with the metal via its lone pair of electrons, blocking the active sites from the reactants and thus deactivating the catalyst. Although reports have indicated that co‐feeding hydrogen sulfide and methane can produce CS_2_, severe catalyst deactivation usually occurs when H_2_S is introduced into the DRM process [15, 16, 17]. A typical DRM metal catalyst nickel is especially susceptible to sulfur poisoning. It has a reaction‐free energy (Δ*G*°) of approximately −100 kJ/mol S_2_ at 1000 K, making it highly vulnerable to even trace amounts of sulfur [18].

Efforts to mitigate this issue have centered on three key strategies: the addition of promoters, optimization of support materials, and incorporation of noble metals. Specifically, rare earth metals (e.g., La and Ce) facilitate the oxidation of sulfur into SO_2_, thereby accelerating sulfur removal [19]. In contrast, transition metals such as W enhance the dissociation of Ni‐S bonds; the resulting W‐S species can then be removed by reaction with H_2_ to form H_2_S [20]. These approaches share a common mechanism: they introduce sacrificial components that preferentially bind to sulfur species. By sequestering these poisons, the sacrificial components delay catalyst deactivation. However, a critical limitation is that these sacrificial materials are eventually fully consumed, providing only temporary protection. As a result, regular catalyst regeneration is typically required [21, 22, 23, 24]. Beyond sacrificial components, some alkaline earth metal‐based supports (e.g., dolomite) can also inhibit the chemisorption of sulfur [25]. Even with this protection, however, a gradual decline in catalytic activity remains unavoidable. Additionally, the incorporation of noble metals offers another route to mitigate sulfur poisoning: H_2_S preferentially adsorbs onto noble metals and can be easily desorbed, reducing its impact on the catalyst [26]. Nevertheless, all the aforementioned strategies address sulfur poisoning in a reactive manner and thus fail to sustain high DRM activity when exposed to H_2_S. A more durable long‐term solution lies in the intrinsic design of catalysts, namely engineering catalysts that retain their activity even when catalyst poisons are present.

The exceptional metal‐support interaction between cerium oxide (CeO_2_) and metals stems from its ability to dynamically store and release oxygen atoms within its lattice. This enables it to act as an active participant rather than an inert carrier, directly promoting redox reactions [27, 28, 29]. Moreover, oxygen migration from the CeO_2_ support toward the metal surface has been observed in metal‐CeO_2_ systems (e.g., Pt [30, 31], Rh [32], Pd [33], and Cu [34]). This oxygen transfer enables the catalyst to clean itself: oxygen supplied from CeO_2_ oxidizes carbon species on the metal surface, removing them as CO or CO_2_. Lykhach et al. demonstrated the effect of this oxygen overflow on carbon removal in Pt/CeO_2_ catalysts [35]. Inspired by the regenerative behavior, we hypothesized that reverse lattice oxygen spillover (RLOS) at the metal–CeO_2_ interface could prevent sulfur poisoning by continuously oxidizing and removing deposited sulfur.

In this study, we demonstrated that CO_2_‐induced RLOS in Pt/CeO_2_ converts strongly adsorbed H_2_S into weakly bound SO_2_, enabling the continuous removal of sulfur poisons and restoring DRM activity. Density functional theory (DFT) calculations reveal a low energy barrier for lattice oxygen migration at the Pt–O–Ce interface and a low oxidation energy barrier contributed by Pt, making this interface uniquely capable of self‐regeneration after H_2_S poisoning. Isotope‐assisted temperature‐programmed surface reaction (isotope‐TPSR) experiments confirm that the Pt–O–Ce interface inhibits sulfur buildup and promotes its oxidation. This behavior distinguishes Pt/CeO_2_ from other CeO_2_‐supported catalysts with different metals or Pt catalysts supported by non‐oxide materials. Furthermore, we synthesized atomically dispersed Pt/CeO_2_ catalysts with adaptive metal‐support interactions, which can stabilize the catalyst structure. Benefiting from the dynamic balance between sulfur deposition and desorption, the catalyst maintained stable DRM performance for 100 h even under the condition of 10 ppm H_2_S. Our findings demonstrate that CO_2_‐induced RLOS at the Pt–O–Ce interface sustains catalytic activity in sulfur‐rich feeds, one of the main bottlenecks in biogas reforming.

## 2 Results and Discussion

### 2.1 Structural Characterization and Performance Evaluations

The CeO_2_‐supported platinum (Pt/CeO_2_) catalyst was synthesized by impregnating the support surface with metal precursors and subsequent high‐temperature treatment for enhanced atomic dispersion [36]. A certain amount of tetraammineplatinum nitrate was loaded onto the CeO_2_ nanorods, which were then annealed at 800°C for 5 h under an air atmosphere. As illustrated in x‐ray diffraction (XRD) patterns (Figure S1), the observed diffraction peaks align with fluorite CeO_2_ (PDF #43–1002), while no distinct peaks corresponding to Pt metal or PtO_2_ were identified, indicating a high dispersion of Pt species within the samples. This is further supported by high‐resolution transmission electron microscopy (HR‐TEM) and elemental mapping (Figure S2), which show no visible Pt nanoparticles (NPs) and a uniform distribution of Pt throughout the CeO_2_ support. To further reveal the local coordination structure of Pt single atoms on the as‐calcined Pt/CeO_2_ catalyst, the extended x‐ray absorption fine structure curve fitting was performed, and the results are plotted in R space (Figure S3 and Table S1). The absence of Pt–O–Pt and Pt–Pt coordination shells and the exclusive observation of Pt–O and Pt–O–Ce coordination shells further evidenced the formation of Pt single atoms on calcined Pt/CeO_2_.

**FIGURE 1:**
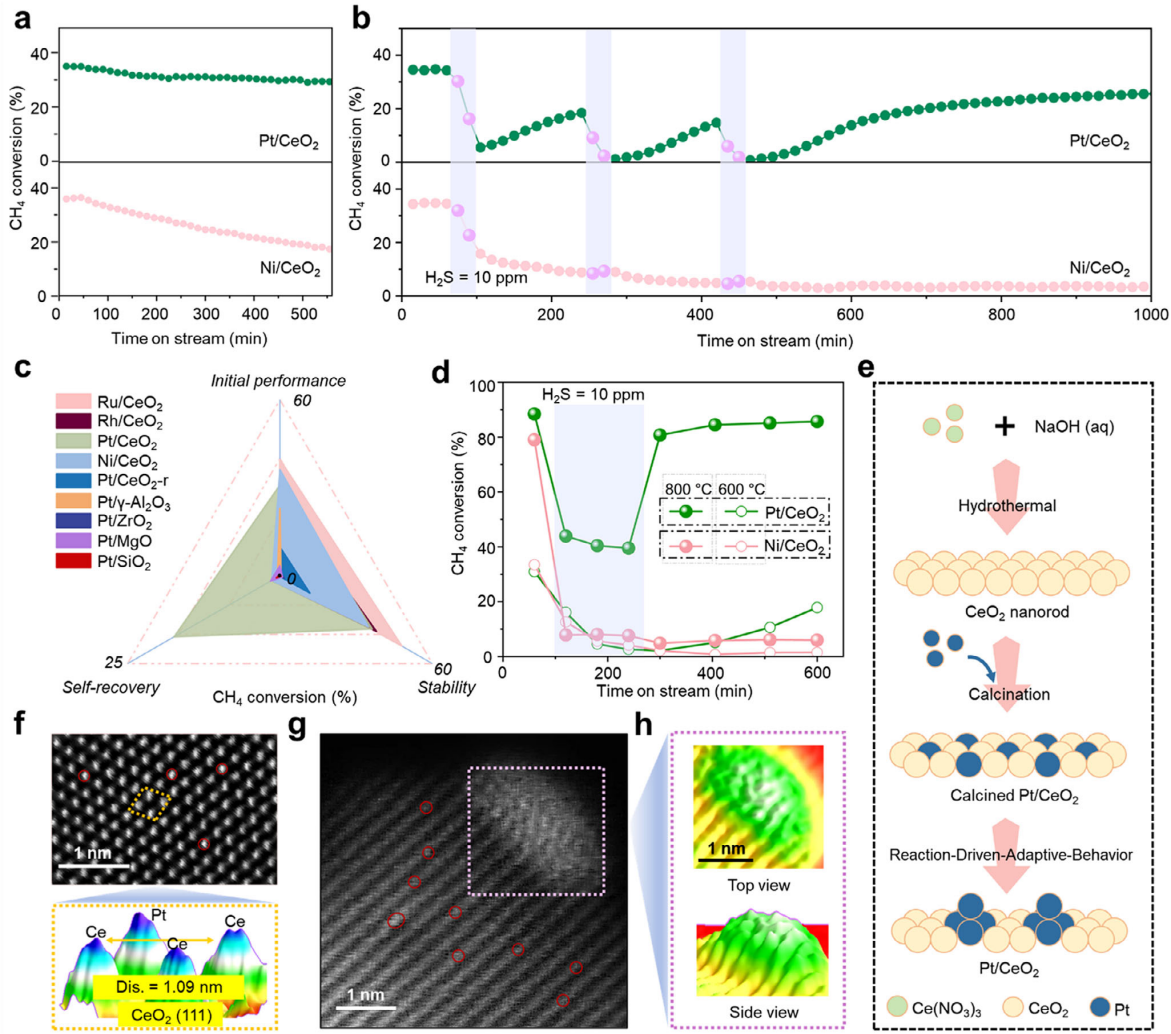
Catalytic performance and structure of catalysts. CH_4_ conversion (a) without and (b) with sequential poisoning of H_2_S over Pt/CeO_2_ and Ni/CeO_2_ catalysts at 600°C. The period highlighted with a light purple column indicates that 10 ppm H_2_S was introduced. (c) DRM performance comparison (Initial performance: initial DRM activity within 1 h, Stability: 10‐h DRM activity and self‐recovery: DRM activity after 6 h of self‐recovery after H_2_S‐poisoning). (d) CH_4_ conversion of Pt/CeO_2_ and Ni/CeO_2_ catalysts in the presence or absence of H_2_S at 600°C and 800°C, respectively. (e) Schematic diagram of reaction‐driven adaptive behavior. (f) AC‐HAADF‐STEM image of Pt/CeO_2_ and 3D atom‐overlapping Gaussian‐function fitting mapping of the yellow dashed rectangle. (g) AC‐HAADF‐STEM image of Pt/CeO_2_ after 3 h reaction and (h) 3D atom‐overlapping Gaussian‐function fitting mapping of purple dashed rectangle.

The Pt/CeO_2_ catalyst was evaluated for DRM without a pre‐reduction process in the absence of H_2_S at 600°C. The initial conversions of CH_4_ and CO_2_ were 32% and 42%, respectively (Figure 1a), and after 100 h of reaction, they decreased by 6.9 and 6.3 percentage points, respectively (Figure S4). Atomically dispersed Pt aggregated to ∼3.0 nm after 20 h of reaction (Figure S5) and remained around this size after 100 h (3.2 nm; Figure S6), suggesting that most Pt aggregation occurred during the initial reaction period. This phenomenon has been observed in other reports due to the presence of reducible gases H_2_, CO, and CH_4_ [37]. Moreover, as shown in the aberration‐corrected high‐angle annular dark‐field scanning transmission electron microscopy (AC‐HAADF‐STEM) image (Figure S7), the Pt NPs were partially embedded in the CeO_2_. This was due to the adaptive metal‐support interaction, which manifests as a tripartite equilibrium between the reaction environment, catalyst structure, and activity. However, the higher oxygen vacancy formation energy of Ni/CeO_2_ (3.73 eV) compared to Pt/CeO_2_ (2.61 eV) leads to severe methane deep cracking, which severely limits the activity and stability of the Ni/CeO_2_ catalyst (Figures S8 and S9). Additionally, there was no carbon deposition after 100 h of DRM reaction, as confirmed by both the thermogravimetric analysis (TGA) results (Figure S10) and the Raman spectra (Figure S11). We attribute the strong metal‐support interaction (SMSI): once Pt NPs form, their partial encapsulation by CeO_2_ inhibits further sintering and helps maintain an active metal surface. As a result, the catalyst sustains its DRM activity with negligible coke deposition.

When 10 ppm of H_2_S was introduced, the DRM activity of the Pt/CeO_2_ catalyst experienced an instant and dramatic drop. However, once the H_2_S was stopped, the activity began to recover (Figure S12). It should be noted that the 10 ppm H_2_S concentration used here reflects realistic post‐desulfurization levels in biogas or natural‐gas feeds (typically < 15 ppm), where H_2_S is continuously present at trace levels during operation. After three cycles of H_2_S poisoning, the Pt/CeO_2_ catalyst still exhibited excellent recovery performance. In contrast, when Pt was replaced by Ni, the Ni/CeO_2_ catalyst experienced continuous deactivation (Figure 1b) despite its constant DRM performance in the absence of H_2_S (Figure S13). Compared to the complete deactivation of Ni/CeO_2_ catalysts, the self‐recovery efficiency of Pt/CeO_2_ catalysts can reach 62% of the original CH_4_ conversion after 6 h (Figure 1c). In addition, we also evaluated different CeO_2_‐supported metal catalysts, including Rh, Ru, and Pd. Although all the CeO_2_‐supported noble metal catalysts displayed comparable or even superior initial DRM activity to Pt/CeO_2_, the DRM activity of the other CeO_2_‐supported noble metal catalysts cannot recover within 15 h after poisoning by H_2_S (Figures S14–S16). Furthermore, we replaced CeO_2_ support with other metal oxides, such as SiO_2_, ZrO_2_, γ‐Al_2_O_3_, and MgO, while keeping Pt as the active metal (Figure S17, Table S2). Due to their limited electron transfer capability with Pt and insufficient oxygen supply capacity, sintering of active sites and severe coking occur during the reaction process. This results in their activity and stability being lower than that of the Pt/CeO_2_ catalyst. However, when the Pt/CeO_2_ catalyst was reduced at 800°C (Pt/CeO_2_‐r), aggregated Pt NPs (∼6.5 nm) were formed during this period, thus minimizing the SMSI effect in the catalysts (Figure S18). As a result, the initial DRM activity decreased to approximately 10%, lower than that of the Pt/CeO_2_ catalyst at 600°C. Correspondingly, the self‐recovery after H_2_S poisoning is sluggish, with no observable recovery within 500 min and only slight recovery (∼2.5%) when the duration was extended to 760 min (Figure S19). According to the results above, the combination of Pt and CeO_2_ is crucial to ensure both excellent DRM activity and superior self‐recovery performance. Therefore, it can be concluded that the abundance of Pt–O–Ce interface sites promotes the recovery of activity. However, the activity cannot be fully restored at 600°C. When the reaction temperature was set at 800°C to accelerate the self‐recovery of H_2_S‐poisoned catalysts, constant activity in the presence of H_2_S can also be obtained (Figure 1d). In contrast, the Ni/CeO_2_ catalyst continued to suffer from irreversible deactivation in the presence of H_2_S, even at 800°C. The changes in the catalytic performance of Pt/CeO_2_ and Ni/CeO_2_ in CO_2_ conversion due to the introduction of H_2_S also showed the same trend as those in CH_4_ conversion (Figure S20). Similarly, Pt/CeO_2_ exhibits similar reaction‐driven adaptive behavior in the presence of H_2_S (Figure 1e). The atomically dispersed Pt species (Figure 1f) evolve into stable Pt NPs (Figure 1g) through a specific reaction process. Similarly, a flat surface was also identified by applying a Gaussian function to the Pt NPs. The consistent lattice fringes of Pt and CeO_2_ at the interface suggest a robust Pt–O–Ce interaction (Figure 1h).

### 2.2 Investigating the Effects of Sulfur

Before investigating the catalyst poisoning mechanism, we identified the reaction sites using infrared spectroscopy, Raman spectroscopy, and DFT calculations. In situ diffuse reflectance infrared Fourier transform spectroscopy (DRIFTS) (Figures S21–S23) identified key intermediates (CH_x_, CH_x_O) during CH_4_ activation on Pt/CeO_2_, directly linking its high activity to the Pt–O–Ce interface. Subsequently, steady‐state isotopic transient kinetic analysis (SSITKA) combined with DRIFTS (Figure S24) further demonstrated that CH_x_O* does not originate from CO_2_ hydrogenation; rather, CO_2_ preferentially adsorbs on CeO_2_ oxygen vacancies (Figure S25). Furthermore, isotope‐Raman (Figures S26 and S27) clearly revealed distinct activation sites: CH_4_ at Pt–O–Ce (inducing F2g red shift) versus CO_2_ on CeO_2_ (causing blue shift). Consistently, DFT calculations confirmed minimal CO_2_ adsorption on Pt but strong adsorption on CeO_2_ (Figures S28 and S29). The results indicate that the active sites for CH_4_ and CO_2_ in Pt/CeO_2_ are distinct, specifically Pt–O–Ce and CeO_2_, respectively.

**FIGURE 2:**
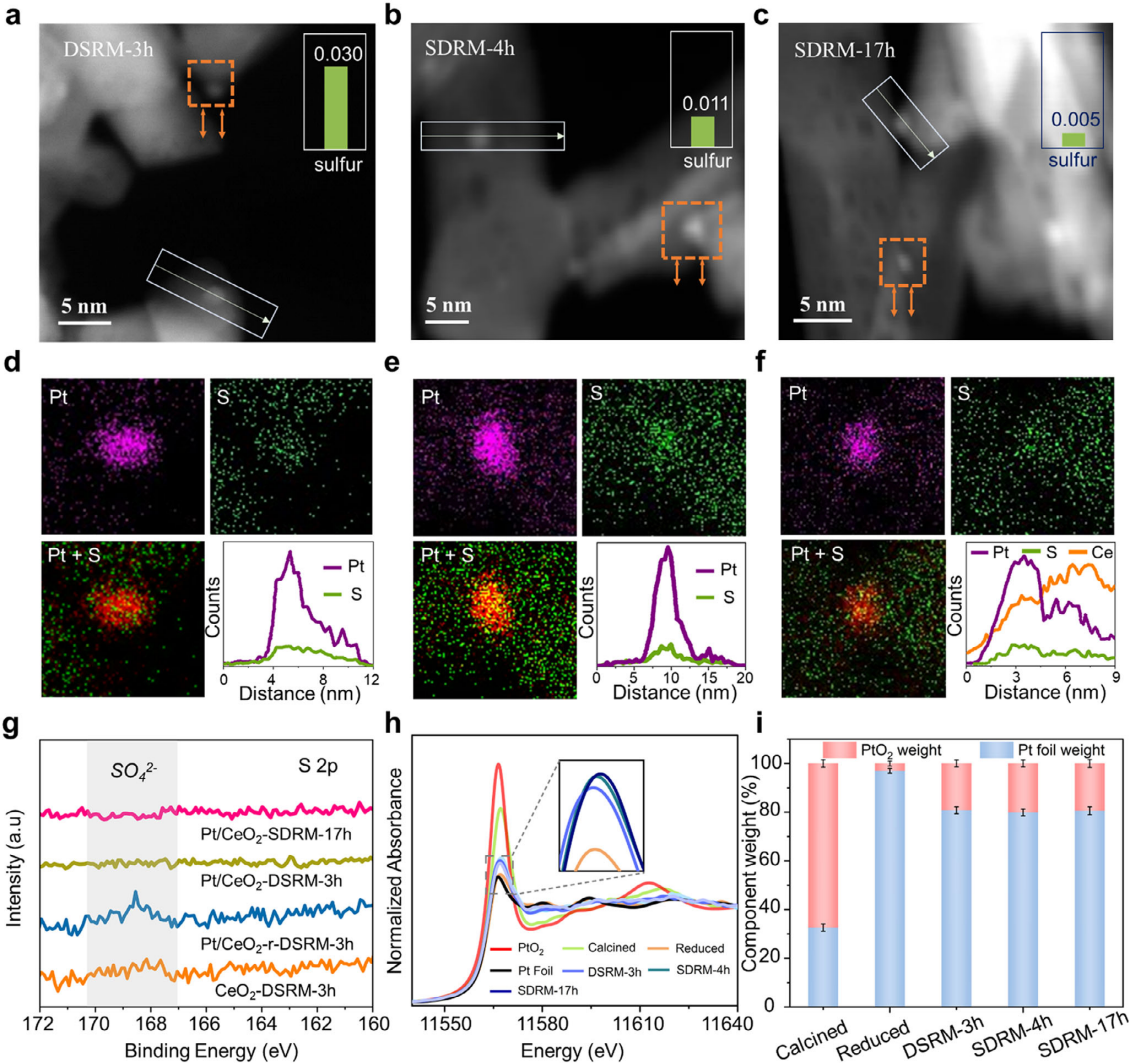
Morphological and surface structural characterizations of Pt/CeO_2_ at different conditions. HADDF‐TEM images of (a) DSRM‐3 h, (b) SDRM‐4 h, and (c) SDRM‐17 h. The inset column figure shows the sulfur content originated from the carbon and sulfur content analyzer. Elemental mapping and line scan EDS spectra were collected at the positions labeled by the orange box (mapping) and white rectangle (line scanning) in (a–c), respectively, corresponding to (d) DSRM‐3 h, (e) SDRM‐4 h, and (f) SDRM‐17 h. (g) XPS S 2p spectra of various catalysts. (h) XANES images (green dashed box zoomed in to get the tangible white line peak signal image) and (i) The linear combination fitting (LCF) results for various catalysts. Error bars represent the fitting uncertainties listed in Table S6.

Quasi‐in situ characterization experiments were performed to analyze the surface structural evolution of the Pt/CeO_2_ catalyst during the self‐recovery process. For convenience, various states of the catalysts are represented by abbreviations. The calcined Pt/CeO_2_ catalyst (Calcined) was treated via two different routes: one involved reduction at 800°C (Reduced); the other consisted of initial exposure to 10 ppm H_2_S for 3 h (DSRM–3 h), followed by regeneration under DRM atmosphere for 4 h (SDRM–4 h) and 17 h (SDRM–17 h), respectively. The High‐angle annular dark‐field transmission electron microscopy (HAADF‐TEM) images presented in Figure 2a–c indicate that the size of the Pt NPs remained stable as the self‐recovery time increased. We conducted regional energy dispersive spectroscopy (EDS) elemental mapping and line scan analysis for each sample, as illustrated in Figure 2d. It should be noted that, given the spatial resolution of EDS mapping, part of the sulfur signal may also originate from sulfur species located on nearby CeO_2_ surfaces or at the Pt–O–Ce interface. Nevertheless, the strongest overlap coincides with Pt‐rich regions, implying preferential sulfur accumulation near the active metal sites. Additionally, elemental line scan analysis, as depicted in Figure 2f, revealed that the Pt NPs were partially embedded within CeO_2_ due to the reaction‐induced SMSI effect. Moreover, the sulfur signal did not coincide with an enhancement of the Ce signal, further confirming the presence of sulfur on Pt. This suggests that while sulfur species may exist at the metal‐support boundary, they are not uniformly distributed across the CeO_2_ surface, but rather concentrated where Pt is present. The accumulation of S over Pt is also confirmed by TPSR after H_2_S‐treatment (Figure S30 and S31). The carbon and sulfur analyzer were used to accurately measure the variation in sulfur content throughout the self‐recovery process. The results indicate that the sulfur content in the Pt/CeO_2_ catalyst decreased from 0.0–3 to 0.005 wt% as the in situ self‐recovery time was extended (Table S3), providing direct evidence for the removal of S over the poisoned catalyst. This bulk analysis complements the microscopic observations, confirming that the apparent surface cleaning is accompanied by actual sulfur loss from the material. On the contrary, the Rh/CeO_2_, Ru/CeO_2_, and Ni/CeO_2_ catalysts retained high levels of sulfur residuals even after up to 15 h of self‐recovery time (Table S4).

The electronic structures of the surface species were investigated using quasi‐in situ x‐ray photoelectron spectroscopy (XPS) to reveal how the oxidation states of Pt and the nature of sulfur species evolve during the recovery process. Samples including pure CeO_2_, Pt/CeO_2_‐r, and Pt/CeO_2_ that were exposed to 10 ppm H_2_S for 3 h are designated as CeO_2_‐DSRM‐3 h, Pt/CeO_2_‐r‐DSRM‐3 h, Pt/CeO_2_‐DSRM‐3 h, respectively. The H_2_S‐poisoned Pt/CeO_2_ catalyst treated with CO_2_ and CH_4_ for 17 h is denoted as Pt/CeO_2_‐SDRM‐17 h. The S 2p spectra of the self‐recovery catalysts after H_2_S indicate that CeO_2_ and Pt/CeO_2_‐r catalysts exhibited SO_4_^2−^ signals, while Pt/CeO_2_ catalysts did not display the corresponding signals due to the low sulfur content (Figure 2g) [38]. As the self‐recovery time increases, a slight increase in the surface Pt^0^ content was also observed (Figure S32 and Table S5), originating from the reduction of the Pt–O–Ce interface. Furthermore, x‐ray absorption near‐edge structure (XANES) measurements were conducted. As shown in Figure 2h, it is evident that the intensity of the white line peaks of the fresh Pt/CeO_2_ catalysts was higher and similar to that of PtO_2_, indicating that Pt was predominantly in the high valence state. According to the TEM results, the highly dispersed Pt aggregated into Pt NPs during the DRM reaction and resulted in decreased white line peak intensity, as observed in Figure 2h. The introduction of H_2_S resulted in a slight increase in the intensity of the white line peaks, indicating a higher oxidized state of Pt (Figure 2i and Table S6) due to the strong coordination ability of S. The self‐recovery time did not significantly change the oxidation state of Pt, indicating that H_2_S poisoning occurred only on the surface of Pt NPs. This observation was further confirmed by in situ DRIFTS spectra of CO absorption. As shown in Figure S33, the re‐exposure of Pt^0^ on the surface of the Pt/CeO_2_ catalyst resulted in an enhanced CO signal in the adsorbed state of Pt^0^ with prolonged self‐recovery time. According to the results above, the poisoning of H_2_S primarily occurs on the surface of Pt NPs, leading to decreased activation of CH_4_ and CO_2_. The self‐recovery mechanism is governed by surface sulfur oxidation and removal, which directly controls the catalyst's regained activity.

### 2.3 Insight Into Self‐Recovery Mechanism After H_2_S Poisoning

**FIGURE 3:**
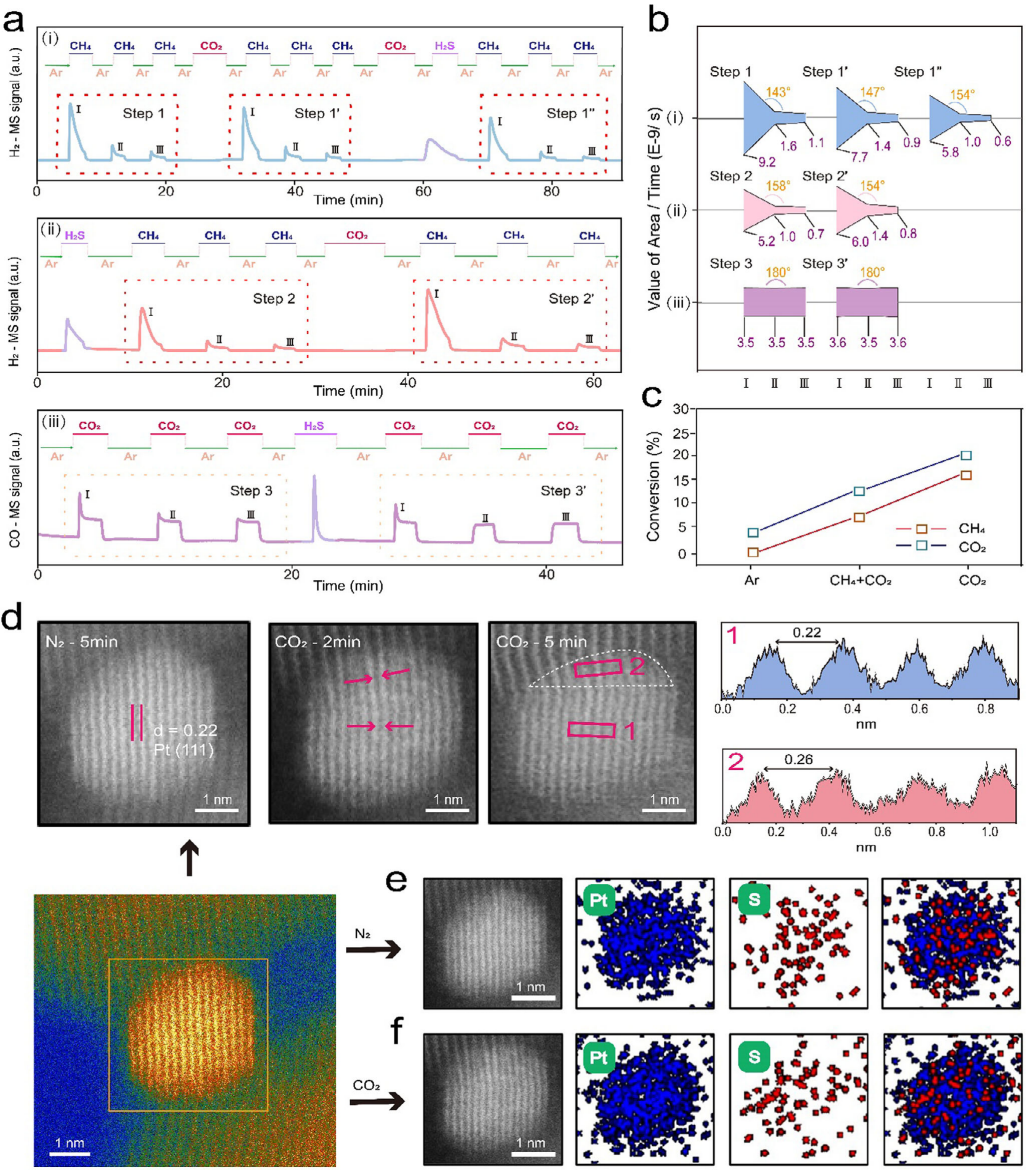
On‐line and in situ detection of poisoning sites on the surface of Pt/CeO_2_ catalysts. MS signals were detected throughout the entire process of products: (a) H_2_ and CO. (b) Corresponding integrating peak area normalized by the time obtained during multi‐pulse coupling online MS experiments at 600°C over Pt/CeO_2_ catalysts to explore the effect H_2_S for the activation of CH_4_ and CO_2_. (c) Statistical results of initial activity after 3 h treatment in different atmospheres. The flow of CH_4_ or CO_2_ remained at 25 mL min^−1^, while the total flow rate was adjusted to 100 mL min^−1^. (d) In situ structural evolution sequence with corresponding line profiles of the Pt/CeO_2_ catalyst after 3 h of H_2_S poisoning. Elemental distribution map (e) After 5‐min N_2_ exposure and (f) Subsequent elemental map following 5‐min CO_2_ treatment.

To investigate the mechanism of H_2_S poisoning, we cyclically introduced CH_4_ or CO_2_ at 600°C to distinguish how each reactant interacts with the catalyst surface before and after poisoning. By analyzing the variations in the peaks of the product signals pre‐ and post‐H_2_S exposure, the relevant outcomes were derived, as illustrated in Figure 3a. The catalysts were pretreated by DRM for 3 h before testing to ensure that the surface configuration was consistent between the on‐line and pulse experiments. Based on the signal values depicted in Figure 3a(i) for Step1 and Step1', it can be observed that the H_2_ signal of the Pt/CeO_2_ catalyst experienced a gradual decrease. This decline could be attributed to the blockage of active sites caused by extensive methane cracking or the accumulation of CH_x_ species after CH_4_ introduction. After introducing a certain amount of CO_2_, the activation ability of CH_4_ was restored, indicating that the carbon formed during CH_4_ activation is amorphous and can be converted to CO by CO_2_ (Figure S34). When the regenerated active sites (assisted by CO_2_) were poisoned by H_2_S, the activation of CH_4_ was inhibited in Step 1''. The emergence of H_2_ during H_2_S treatment indicates the decomposition of H_2_S, with residual sulfur binding to the active sites. This confirms that H_2_S not only blocks active Pt sites but also contributes sulfur species that remain anchored to the surface. To eliminate the influence of deposited carbon, the catalyst was treated with H_2_S followed by CH_4_ (Figure 3a[ii]). The subsequent intensity of the H_2_ signal is lower than that in Step 1'. It is worth noting that an increased H_2_ signal appeared in Step 2' after CO_2_ treatment, suggesting that CO_2_ may help regenerate the active sites poisoned by H_2_S. This phenomenon can also be confirmed by the fact that H_2_S hardly affects the activation of CO_2_, as shown in Figure 3a(iii).

Simultaneously, comparing peak data using area‐versus‐time normalized values provides a more intuitive observation of product quantity changes. As shown in Figure 3b, the numbers marked in purple represent peak intensities, with larger values indicating stronger product signals (higher = stronger signal). The angles marked in yellow represent the slope angles of peak decay rates, with larger angles indicating a smaller rate of change and steeper slopes corresponding to faster decay. It is evident that the initial peak data (9.2 > 5.2) was recorded in both Steps 1 and 2. This drop in peak intensity clearly shows that H_2_S exposure reduced the number of active sites capable of CH_4_ activation. Furthermore, the greater activity loss upon H_2_S poisoning compared with carbon accumulation indicates that sulfur blocking, rather than carbon deposition, is the primary cause of deactivation. The weaker signal values observed in CH_4_‐TPSR in the last two pulses of each batch can be attributed to the fact that the site of CH_4_ activation in this session was CeO_2_, which has a weaker CH_4_ activation capacity at 600°C. In contrast, the CH_4_ conversion ability was improved after treatment with CO_2_, suggesting that CO_2_ might contribute to the self‐recovery of the H_2_S‐poisoned Pt/CeO_2_ catalyst. Comparing the H_2_ signals between Step 2 and Step 1″ further confirms severe sulfur poisoning after initial exposure, compounded by carbon deposition. This is attributed to competition between the H_2_S poisoning site and the carbon accumulation site, which is consistent with other studies [39, 40].

Based on the above observed phenomenon, we concluded that H_2_S preferentially poisons the sites responsible for CH_4_ activation, that is, Pt. This aligns with previous findings that sulfur strongly binds to metallic Pt, suppressing C–H bond activation. Furthermore, to elucidate the effect of CO_2_ on poisoning self‐recovery process, the Pt/CeO_2_ catalysts were regenerated by using equal flow rates of Ar atmosphere, CO_2_ atmosphere, and CH_4_–CO_2_) atmosphere for 3 h after the catalysts were treated H_2_S poisoning for 3 h, respectively (Figure S35). Notably, we found that the CH_4_ and CO_2_ conversion of Pt/CeO_2_ increased to 15% and 19% when regenerated under the same flow rate of CO_2_.

In contrast, the catalyst activity barely recovered after treatment with Ar. The activity began to recover gradually only upon reintroducing the DRM atmosphere (Figure 3c). Because CH_x_* and C* species formed from CH_4_ activation consume O* from CO_2_, the DRM atmosphere offers fewer available oxygen species for sulfur removal, making it less effective than pure CO_2_ for regeneration. Furthermore, we hypothesize that thermal effect alone is unlikely to facilitate the removal of deposited sulfur. To verify this hypothesis, we conducted controlled‐variable experiments (Figure S36) and temperature programming decomposition under Helium (He‐TPDC) (Figure S37) on the sulfide‐treated Pt/CeO_2_ catalysts. The controlled‐variable experiments demonstrated that the catalyst activity could not be restored even at 800°C under inert gas atmosphere. Meanwhile, no SO_2_ signal was detected throughout the He‐TPDC experiment, which excluded the contribution of thermal decomposition in sulfur removal.

To atomically visualize the catalyst's specificity under CO_2_ atmosphere, we conducted an in situ STEM study on single particles at the Pt/CeO_2_ interface. As shown in Figure 3d, the Pt particle maintained the exposed (111) facet under N_2_ atmosphere, with consistent lattice spacing between its edge and central regions (Figure S38). Upon switching to CO_2_ atmosphere for 2 min, progressive lattice expansion occurs at the particle periphery. Subsequent prolonged CO_2_ exposure revealed that the non‐interfacial region of the Pt particle retained its configuration with 0.22 nm lattice spacing in the core. Conversely, the CeO_2_‐contacting edge underwent morphological evolution from polyhedral to spherical curvature, accompanied by lattice expansion to 0.26 nm. This localized surface energy reduction was attributed to edge Pt atoms bonding with coordinating oxygen from Ce^4+^, forming Pt^2+^ and Ce^3+^. Then, Ce^3+^ species are rapidly oxidized to Ce^4+^ due to its strong oxygen vacancy replenishment capacity under the atmosphere of CO_2_. Complementary in situ EDS mapping (Figure 3e,f) indicates diminished sulfur content on the Pt surface after 5‐min CO_2_ treatment, with the S/Pt mass ratio decreasing from 0.0120 to 0.0035 (Table S7). Additionally, it is clearly observable that deposited sulfur at the Pt interface is removed to a greater extent than that at the top.

In summary, atmospheric conditions are crucial in facilitating the removal of sulfur deposited on Pt NPs, rather than relying solely on temperature. Impressively, a more significant self‐recovery is observed when CO_2_ is introduced. Hence, it can be concluded that the oxidative gas CO_2_ is essential for the RLOS effect to remove deposited S over the poisoned Pt/CeO_2_ catalyst and achieve effective self‐recovery.

### 2.4 Insights Into RLOS Mechanism

**FIGURE 4:**
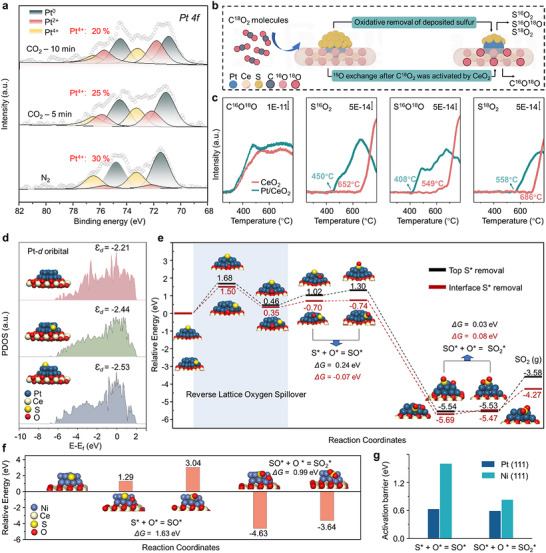
Evidence for lattice oxygen reverse spillover. (a) In situ NAP‐XPS Pt 4f spectra recorded at 600°C after poisoning Pt/CeO_2_ were exposed to N_2_ and CO_2_ in turn. (b, c) Monitoring the signal evolution of isotopically labeled species during C^18^O_2_‐TPSR experiments. (d) The PDOS of Pt/CeO_2_(111) with different configurations (*ε*_d_: *d*‐band center). (e) Electronic energy diagrams of different sulfur removal pathways over Pt/CeO_2_. (f) Electronic energy diagrams of sulfur removal over Ni/CeO_2_. All Gibbs free energy change (ΔG) values were calculated at 873.15 K, including zero‑point energy and entropy corrections. (g) Summary of the reaction activation barriers on Pt(111) and Ni(111).

To probe deeply into the mechanism of CO_2_‐induced sulfur removal, we employed in situ near‐ambient pressure x‐ray photoelectron spectroscopy (NAP‐XPS) to analyze the surface chemical behavior of the catalyst. The Pt 4f spectra were acquired at 600°C for the sulfur‐poisoned Pt/CeO_2_ catalyst following sequential exposure to N_2_ and CO_2_. As shown in Figure 4a, approximately 30% of Pt^4+^ species, attributed to surface‐sulfide Pt, were present on the poisoned catalyst. Upon switching to CO_2_ atmosphere, prolonged treatment progressively reduced the Pt^4+^ content to 20%, indicating desorption of sulfur species bound to Pt. Concurrently, the Pt^2+^ concentration increased alongside metallic Pt exposure, suggesting partial Pt oxidation. Given that metallic Pt is ineffective for CO_2_ activation, the oxygen source for PtO formation is most likely lattice oxygen from the CeO_2_ support. However, NAP‐XPS cannot detect valence changes in Ce due to the significant concentration disparity between Pt and support elements during lattice oxygen transfer to Pt sites. Thus, to validate this hypothesis, we performed a series of temperature‐programmed surface reactions assisted by labeled C^18^O_2_ (isotope‐TPSR) over H_2_S‐pretreated catalysts (Figure 4b,c). C^18^O_2_ was consumed with the production of C^18^O^16^O (m/z = 46) due to the oxygen exchange from CeO_2_. S^16^O_2_ (m/z = 64), S^16^O^18^O (m/z = 66), and S^18^O_2_ (m/z = 68) signals were attributed to the oxidation of deposited sulfur on Pt NPs. Throughout the process, no C^18^OS and C^16^OS signals were detected, excluding the possibility of CO‐assisted sulfur oxidative removal pathways in the system (Figure S39). Simultaneously, by comparing the light‐off temperatures to produce C^16^O^18^O, we can identify the oxygen exchange capacity of different catalysts. As shown in Figure 4c, the light‐off temperature sequence for C^16^O^18^O is Pt/CeO_2_ < CeO_2_, which is attributed to the deposition of Pt NPs on CeO_2_. As demonstrated by H_2_‐TPR, the presence of Pt NPs affects the surface properties of CeO_2_ (Figure S40). The release of SO_2_ during the isotope‐TPSR demonstrates that CO_2_ forces the removal of deposited S via oxidation. By comparing the peak temperatures of the sulfur dioxide curves containing different oxygen isotopes, a refined analysis of the sulfur oxidation process can be achieved. The S^16^O^18^O signal emerged first at 408°C, followed by the S^16^O_2_ signal (450°C) and the S^18^O_2_ signal (558°C). The ignition temperature hierarchy reflects the sequence of oxidation reactions. The first appearance of S^16^O^18^O at a lower temperature for the Pt/CeO_2_ catalyst rather than S^16^O_2_ and S^18^O_2_ indicates that the lattice oxygen at the Pt–^16^O–Ce interface is preferentially reverse spillover onto the Pt NPs, where it first bonds with deposited sulfur, followed by the exchanged ^18^O for the spillover process. Interestingly, the desorption peak in Pt/CeO_2_ is even smaller than that of CeO_2_, demonstrating that the RLOS might have occurred during the H_2_S poisoning process, resulting in much less sulfur deposition. Operationally, we identify CO_2_‐induced RLOS by a triad of signatures: (i) isotope‐TPSR showing S^16^O^18^O appearing before S^16^O_2_/S^18^O_2_, (ii) CO_2_‐specific regeneration (no recovery under Ar at the same temperature), and (iii) NAP‐XPS indicating reduction of high‐bonding energy Pt species upon CO_2_ exposure, consistent with lattice‐oxygen delivery from CeO_2_ to Pt. In simpler terms, the lattice oxygen at the interface of Pt–O–Ce can inhibit excess sulfur deposition on the Pt NPs by oxidation. Additionally, the filling of surface oxygen vacancies by CO_2_ generates an oxygen potential gradient, which drives the migration of lattice oxygen toward Pt. This process significantly enhances the reverse spillover phenomenon.

Calculations show that the oxygen vacancy formation energy of the subsurface in CeO_2_ is lower than that of the surface, and the energy barrier for surface oxygen migration to the oxygen vacancy at the subsurface phase is 0.79 eV, which is much smaller than migration to the surface oxygen vacancy (3.4 eV). This suggests that CeO_2_ tends to transfer oxygen between the subsurface and the surface phase through a two‐step exchange, which facilitates the participation of oxygen from CO_2_ in the reaction cycle of RLOS. This way of surface oxygen filling is favorable for the continuous operation of the reaction (Figure S41).

We performed DFT calculations to gain deeper insights into sulfur deposition on the catalyst and its subsequent oxidative removal. As shown in Figure S42, due to the differences in the geometric coordination effects and electronic structure effects between Pt sites and the Pt–O–Ce interface (or CeO_2_ surface), H_2_S tends to adsorb on Pt sites rather than at the Pt–O–Ce interface or on the CeO_2_ surface, which is consistent with the experimental observations in Figure 2d. An analysis of adsorption energies reveals that H_2_S favors activation and dissociation at specific Pt sites (e.g., HCP: −1.43 eV; Hollow1: −1.42 eV) to form adsorbed sulfur (S*). The deposited sulfur significantly modulates the electronic structure of Pt. Compared to the pristine catalyst (*ε*_d_ = −2.21 eV), S* at different locations induces distinct shifts in the Pt *d*‐band center, all of which attenuate Pt's capacity for adsorbing and activating reactants. Specifically, sulfur at the interface site shifts *ε*_d_ to −2.44 eV, while sulfur at the top site shifts it further to −2.53 eV (Figure 4d).

To understand the recovery mechanism, we compared the oxidative removal of S* from the top and interface sites. The energy barrier for the reverse lattice oxygen spillover—the key step for sulfur oxidation—is lower for interface S* (1.50 eV) than for top‐site S* (1.68 eV) (Figure 4e). Furthermore, the direct combination of lattice oxygen with sulfur is highly endothermic (1.95 eV), unlike the facile oxygen spillover onto Pt (0.46 eV; Figure S43). The oxidation of atomic sulfur proceeds via two steps. For interface S* on Pt/CeO_2_, the first step (S* + O* → SO*) is nearly thermoneutral (Δ*G* = –0.07 eV), and the second step (SO* + O* → SO_2_*) is slightly endothermic (Δ*G* = +0.08 eV). In contrast, for top‑site S* on Pt/CeO_2_, the first step is endothermic (Δ*G* = +0.24 eV), while the second step is nearly thermoneutral (Δ*G* = +0.03 eV). These results indicate that sulfur at the active interface is thermodynamically more facile to oxidize, underpinning the catalyst's self‑recovery capability. For Ni/CeO_2_, the surface stability after reverse spillover of lattice oxygen is significantly lower than that of Pt/CeO_2_ (Figures S44 and S45). The corresponding Gibbs free energy changes for the two sulfur oxidation steps (S* + O* → SO* and SO* + O* → SO_2_*) are +1.63 eV and +0.99 eV, respectively (Figure 4f). The above results indicate that sulfur deposited from the dissociation of strongly adsorbed H_2_S on the Pt/CeO_2_ catalyst is oxidized to weakly adsorbed SO_2_ (Figure S46), thereby enabling continuous sulfur removal and the restoration of DRM activity. In addition, we examined the intrinsic differences in sulfur oxidation kinetics on Pt(111) versus Ni(111) surfaces. The energy barrier for the first oxidation step (S* → SO*) is 0.64 eV on Pt(111) but rises to 1.61 eV on Ni(111). The subsequent step (SO* → SO_2_*) also faces a higher barrier on Ni (0.84 eV) than that on Pt (0.60 eV) (Figure 4g). Specific energy changes are shown in Figure S47. The consistently lower activation barriers on Pt explain the efficient, reversible sulfur removal on Pt/CeO_2_, in stark contrast to the kinetically hindered and thus irreversible sulfur poisoning on Ni/CeO_2_. Specifically, the RLOS at the Pt–O–Ce interface occurs quickly on the Pt/CeO_2_ catalyst, which provides an oxygen‐rich environment for sulfur oxidation. Subsequently, the sulfur‐oxygen bonding reaction is carried out on Pt, which achieves the sulfur removal goal, prompting the catalyst's self‐recovery.

Building on the previous discussion, we have elucidated the underlying reasons for the self‐recovery observed in Pt/CeO_2_ catalysts. Modulating the reaction atmosphere and temperature, without altering the catalyst's composition, proved effective in facilitating the RLOS‐driven regeneration process. The Pt/CeO_2_ catalyst shows incomplete recovery from H_2_S poisoning at 600°C, with removal of residual S occurring at higher temperatures. The temperature was elevated to the optimal range of 700°C–800°C for the DRM reaction with the aim of enhancing the efficiency of the sulfur removal process. The findings were illustrated in Figure S48, where the DRM process exhibited a significant heat absorption reaction. Upon reaching a temperature of 800°C, the Pt/CeO_2_ catalyst demonstrated an enhanced conversion rate of CH_4_ to 87% and CO_2_ to 92%. Furthermore, the catalytic activity remained stable for 100 h during the reaction, as depicted in Figure S49. The coke‐free at 800°C was also verified by in situ Raman (Figure S50). This long‐term durability highlights that sulfur oxidation via RLOS continues efficiently over extended operation. Notably, even after being exposed to H_2_S poisoning for a duration of 100 h, the catalyst still maintained a significant level of CH_4_ and CO_2_ conversion, with 34% and 52% respectively. When the concentration of H_2_S was increased to 100 ppm, the Pt/CeO_2_ catalyst continued to exhibit an equilibrium step, as depicted in Figure S51. This indicated that the system was in equilibrium after balancing the relationship between sulfur adsorption and sulfur desorption, and a relatively stable performance ensued. As a result, the Pt/CeO_2_ catalyst showed excellent resistance to H_2_S poisoning, far exceeding the latest reports to date (Table S8).

**FIGURE 5:**
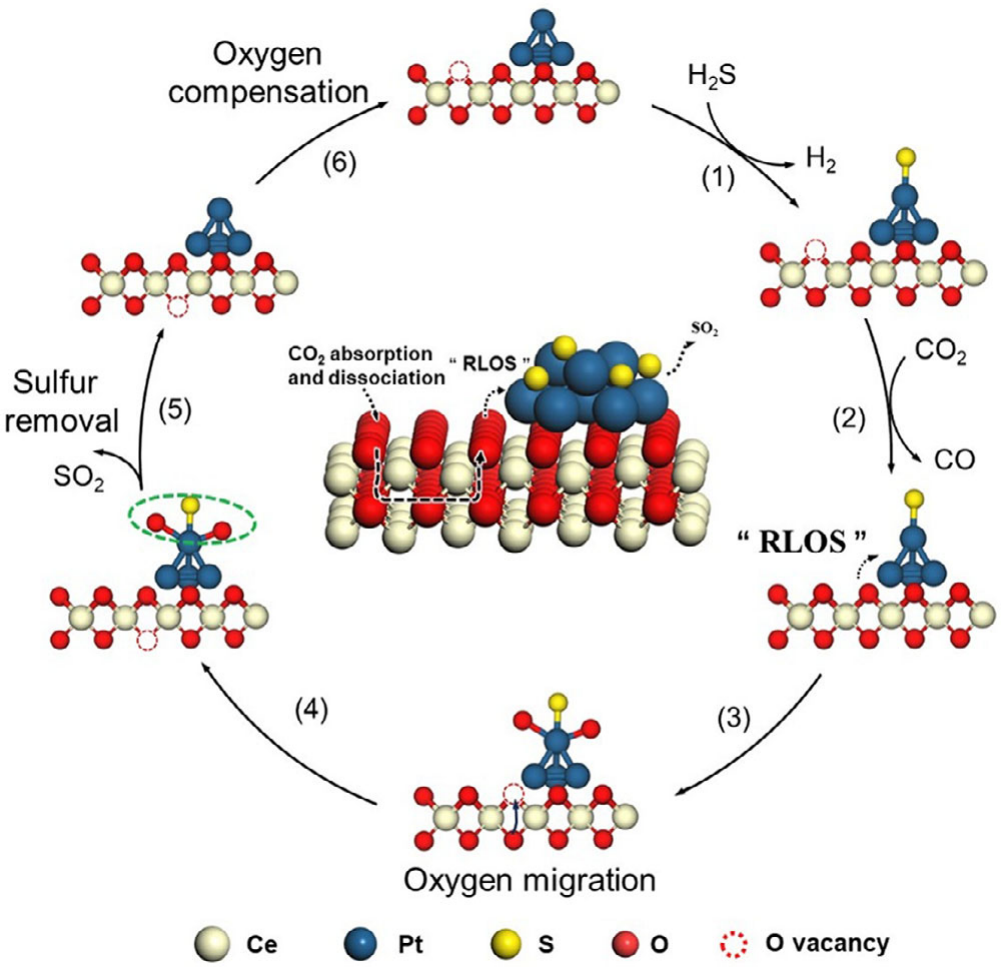
CO_2_‐induced RLOS mechanism for self‐recovery of the H_2_S poisoned Pt/CeO_2_ catalyst.

Based on the experimental results and theoretical calculations, the self‐recovery mechanism of the poisoned Pt/CeO_2_ catalyst was depicted in Figure 5: (1) adsorption and dissociation of H_2_S on Pt NPs lead to catalyst deactivation; (2) CO_2_ is activated by oxygen vacancies in CeO_2_, filling surface vacancies; (3), (4), and (5) lattice oxygen undergoes reverse spillover to Pt, where interactions of S* and O* on Pt reopen the surface active sites; (6) these steps represent the exchange process of oxygen in CeO_2_, which is favorable for the long‐term renewal of the Pt/CeO_2_ catalyst.

## 3 Conclusions

In summary, CO_2_‐induced reverse lattice oxygen spillover from CeO_2_ to Pt significantly accelerates oxidation and subsequent removal of deposited sulfur species. Isotope‐TPSR and DFT calculations demonstrated that RLOS from Pt–O–Ce to Pt experienced a low energy barrier and that Pt effectively oxidizes S to SO_2_. Moreover, abundant Pt–O–Ce interfaces are formed in situ during the DRM process when metal‐dispersed Pt/CeO_2_ is employed as a pre‐catalyst. This interfacial structure amplifies the RLOS effect and sustains DRM activity for 100 h at 800°C in H_2_S‐containing feed. This work provides a clear pathway for designing durable, sulfur‐tolerant catalysts for a broad range of heterogeneous reactions.

## Author Contributions

**Jun Liu**: investigation, writing – original draft, methodology, visualization, formal analysis. **Jiang Deng**: investigation, writing – review and editing, validation, formal analysis, resources, data curation, supervision. **Jiajia Zheng**: investigation, methodology, validation, formal analysis. **Mohsen Beladi Mousavi**: writing – original draft, formal analysis, data curation. **Chunning Sun**: investigation, methodology, formal analysis. **Jin Li**: investigation, methodology, visualization, formal analysis. **Xin Chen**: investigation, methodology, formal analysis. **Yongjie Shen**: investigation, writing – review and editing, validation, formal analysis, data curation. **Haotian Huang**: investigation, methodology, visualization, formal analysis. **Ming Xie**: conceptualization, writing – original draft, data curation. **Emiliano Cortés**: conceptualization, funding acquisition, writing – original draft, supervision, resources, data curation. **Dengsong Zhang**: conceptualization, funding acquisition, writing – original draft, validation, methodology, project administration, data curation, supervision, resources.

## Conflicts of Interest

The authors declare no conflicts of interest.

## Supporting information




**Supporting File 1**: The detailed experimental section and additional figures and tables are listed in the Supporting Information file. The authors have cited additional references within the Supporting Information.

**Supporting File 2**: anie72971‐sup‐0002‐MovieS1.mp4.

**Supporting File 3**: anie72971‐sup‐0003‐MovieS2.mp4.

## Data Availability

The data that support the findings of this study are available from the corresponding author upon reasonable request.
