# Supplementary material for: CO2‐Induced Reverse Lattice Oxygen Spillover on Pt/CeO2 Enables Sulfur‐Resistant Dry Reforming of Methane
Source: Angew Chem Int Ed Engl. 2026 May 31;65(31):e1664469. doi: 10.1002/anie.1664469 (PMC13411365; doi:10.1002/anie.1664469)
Supplement: Supplementary file 1 — Supporting File 1: The detailed experimental section and additional figures and tables are listed in the Supporting Information file. The authors have cited additional references within the Supporting Information. [file ANIE-65-e1664469-s003.docx]

***Supporting Information***

**CO_2_-Induced Reverse Lattice Oxygen Spillover on Pt/CeO_2_ Enables Sulfur-Resistant Dry Reforming of Methane**

Jun Liu,^[a]^ Jiang Deng,*^[a]^ Jiajia Zheng,^[a]^ Mohsen Beladi Mousavi,^[b]^ Chunning Sun,^[c]^ Jin Li,^[a]^ Xin Chen,^[a]^ Yongjie Shen,^[a]^ Haotian Huang,^[a]^ Ming Xie,^[d]^ Emiliano Cortés,*^[b]^ Dengsong Zhang*^[a]^

[a] J. Liu, Assoc.Prof. J. Deng, J. Zheng, J Li, X. Chen, Assoc. Prof Y. Shen, H, Huang, Prof. D. Zhang
International Joint Laboratory of Catalytic Chemistry, State Key Laboratory of Advanced Special Steel, Innovation Institute of Carbon Neutrality, Department of Chemistry, College of Sciences, Shanghai University, Shanghai 200444, People’s Republic of China
E-mail: [jiangdeng@shu.edu.cn](mailto:jiangdeng@shu.edu.cn) and [dszhang@shu.edu.cn](mailto:dszhang@shu.edu.cn)

[b] Dr. M. Beladi Mousavi, Prof. E. Cortés
Nanoinstitute Munich, Faculty of Physics, Ludwig-Maximilians-Universität (LMU), Munich 80539, Germany.
E-mail: [Emiliano.Cortes@lmu.de](mailto:Emiliano.Cortes@lmu.de)

[c] Dr. C. Sun
Inorganic Chemistry and Catalysis Group, Debye Institute for Nanomaterials Science & Institute for Sustainable and Circular Chemistry, Utrecht University, Utrecht 3584 CG, The Netherlands.

[d] Prof. M. Xie
Department of Chemical Engineering, University of Bath, BA27AY, Bath, UK.

**1. Materials and Reagents**

Cerium (III) nitrate hexahydrate (99.99%) was strictly maintained from the use of Shanghai DiYang Chemical Industry Co. Ltd. Tetraammineplatinum (II) nitrate, palladium nitrate solution (18% palladium basis), ruthenium nitrosyl nitrate, rhodium nitrate, γ-Al_2_O_3_, ZrO_2_, MgO, and SiO_2_ were purchased from Sigma Aldrich. Deionized water was used in all experiment steps. Argon (99.99%), methane (99.99%), carbon dioxide (99.99%), and H_2_S (250 ppm, balance Ar) were supplied by Shanghai Youjiali Speciality Gases Co. Ltd. All chemicals or gases were used directly in the received state without purification.

All of the chemicals were of analytical reagent grade and used as received without any further purification.

**2. Experimental Section**

**Synthesis of CeO_2_ nanorods**

50 ml of NaOH solution (7.2 mmol·mL^-1^) was slowly added into 10 ml of Ce (NO_3_)_3_ solution (0.4 mmol·mL^-1^) under vigorous stirring at room temperature. After 30 minutes, the precursor suspension was placed into a Teflon autoclave (100 mL) for 24 h at 100 °C. Finally, the precipitate was washed to neutrality, filtered, and then dried at 80 °C overnight for standby application.

**Synthesis of metal/CeO_2_ catalysts**

In a typical preparation, 300 mg of CeO_2_ powder was added to 10 mL of deionized water at room temperature and stirred vigorously for 1.5 h. A certain amount of tetraammineplatinum solution (20 mg·mL^-1^) was added drop by drop to the above suspension and stirred continuously for 1 h. The product was then obtained by rotary evaporation and subsequently calcined in a static air at 800 °C for 5 h with a heating rate of 5 °C·min^-1^. The final samples were denoted as Pt/CeO_2_. Consistent with the synthesis method described above, the tetraammineplatinum nitrate solution was replaced by ruthenium nitrate solution, palladium nitrate solution, rhodium nitrate solution, and nickel nitrate solution to obtain catalysts known as Ru/CeO_2_, Pd/CeO_2_, Rh/CeO_2_, and Ni/CeO_2_, respectively.

**Synthesis of Pt/oxides catalysts**

By replacing ceria powder with MgO, γ-Al_2_O_3_, ZrO_2_, and SiO_2_, catalysts which denoted as Pt/MgO, Pt/γ-Al_2_O_3_, Pt/ZrO_2_, and Pt/SiO_2_ can be obtained. It should be noted that, considering the strength of the metal-support interaction, a calcination temperature of 600 °C was used for samples Pt/γ-Al_2_O_3_ and Pt/SiO_2_ in order to prevent large-scale sintering of the active sites.

**Catalyst activity test**

The DRM reaction was carried out in a quartz fixed-bed tubular reactor (QUZHOU CITY YEODE RENAISSANCE Co. Ltd.). Catalyst in quartz tubes with an inner diameter of 8.0 mm at ambient pressure. The specific steps are as follows: 120 mg of catalyst diluted in 80 mg of 20-40 mesh quartz sand was added to quartz tube for activity and stability tests under a total gas flow rate of 100 mL·min^-1^ (CH_4_ : CO_2_ = 25 : 25, WHSV of 50,000 mL·g_cat_^-1^·h^-1^). The H_2_S poisoning experiment involved the introduction of 10 ppm or 100 ppm H_2_S for a duration of 3 hours, following the attainment of initial catalyst activity during the DRM reaction for 1 hour. When 10 ppm H_2_S was introduced, the feed was adjusted to CH_4_ ( 25 mL·min^-1^), CO_2_ ( 25 mL·min^-1^), Ar ( 46 mL·min^-1^), and 4 mL·min^-1^ of a 250 ppm H_2_S‑Ar mixture. The H_2_S‑Ar and pure Ar flowmeters were adjusted simultaneously during gas switching. Similarly, when 100 ppm H_2_S is introduced, the Ar flow rate is set to 10 mL·min^-1^, and the H_2_S-Ar mixture flow rate is 40 mL·min^-1^. Periodic shutdown of H_2_S was implemented in cyclic poisoning self-recovery experiments. The gaseous products were identified in real-time using a chromatography instrument (GC-2014C) that was outfitted with a TDX-01 column.

The conversion of CH_4_ and CO_2_ was calculated as follows:

$\text{Conversion}_{\text{CH}_{\text{4}}}\text{=}\frac{{\text{F}\text{CH}}_{\text{4, in}}\text{-}{\text{F}\text{CH}}_{\text{4, out}}}{{\text{F}\text{CH}}_{\text{4, in}}}\text{ ×}$100%

$\text{Conversion}_{\text{CO}_{\text{2}}}\text{=}\frac{{\text{F}\text{C}\text{O}}_{\text{2}\text{, in}}\text{-}{\text{F}\text{C}\text{O}}_{\text{2}\text{, }\text{out}}}{{\text{F}\text{C}\text{O}}_{\text{2}\text{, in}}}\text{ ×}$100%

Where F_in_ and F_out_ represent the flow rate of the inlet and outlet.

The H_2_/CO ratio of product was determined by:

$$\frac{\text{H}_{\text{2}}}{\text{CO}}\text{ =}\frac{\text{H}_{\text{2}}\text{ }\text{produced }\left( \text{mol} \right)}{\text{ CO produced (mol) }}$$

**Characterizations**

X-ray diffraction (XRD) experiments were carried out using a powder diffractometer (Bruker D8 Advance) with Cu-Kα1 radiation and a LynxEye_XE-T linear detector. The morphology and structure of samples and corresponding line scan and EDS mapping data were recorded on a JEOL JEM-F200 (Japan) high resolution transmission electron microscope. AC-HAADF-STEM images were performed on a Thermo Fisher Themis ETEM G3 electron microscope. In situ STEM measurements were performed with a JEM-ARM200F microscope operating at 200 kV. The catalyst was loaded onto EAT-33AA-10 that a microelectromechanical system heating chip with hole type. Thermogravimetric analysis (TGA) was performed on NETZSCH STA 449 F1. 10 mg of the sample was used in each measurement and heated to 800 °C at 10 °C·min^-1^ under air atmosphere. The analysis of the surface chemical state and composition of the samples were conducted using quasi in situ XPS (AXIS Kratos Supra+, AlKɑ source), employing contaminant carbon (C 1s = 284.8 eV) as a reference standard. In situ NAP-XPS experiments were performed with a lab-based NAP-XPS system (ESCALAB 250Xi, Al Kα X-ray source at 50 W). In situ CO-DRIFTS experiments were measured over Bruker TensorⅡinstrument with MCT detector. Quasi-in situ XAS experiments were conducted at the BM23 beamline of the European Synchrotron Radiation Facility (ESRF) during the experiment MA-6128.^[1, 2]^ In situ DRIFTS spectra was collected on Nicolet is50 FT-IR spectrometer with an MCT detector. Temperature-programmed experiments were performed on the Micromeritics AutoChem II 2920 instrument. Raman spectra of the samples were acquired by a LABRAM HR EVO spectrometer. The actual metal content of samples was determined by inductively coupled plasma optical emission spectroscopy (ICP-OES) on an ICPE-9000 (SHIMADZU, Japan). The carbon and sulfur contents in the pretreated catalysts were measured by LECO CS 600CR analyzer. The details of the experimental methods are specifically described in the supporting information.

**Characterizations methods**

**In situ DRIFTS measurements**

In situ DRIFTS spectra was collected on Nicolet is50 FT-IR spectrometer. The samples were first pretreated at 300 °C for 30 minutes in an Ar atmosphere, and then the background was collected at 600 °C. Afterwards, CH_4_ or CO_2_ gas was introduced and spectra was collected at the corresponding temperatures.

**In situ CO**-**DRIFTS measurements**

The 10 mg catalysts were filled into the Harrick Cell and were treated at 300 °C for 60 min. The catalysts were cooled to 30 °C under the atmosphere of 20 mL Ar. Then, 20 mL 2 vol% CO/Ar was introduced for 30 min. Subsequently, 20 mL Ar are purged for another 30 min. The DRIFTS are recorded during the whole process.

**H_2_**-**TPR**

H_2_-TPR experiments were performed on the Micromeritics AutoChem II 2920 instrument. The sample that had been calcined underwent pretreatment in an atmosphere of Ar at a temperature of 300 °C for a duration of 30 minutes. Subsequently, following a cooling process to 50 °C, the sample was subjected to heating up to 800 °C at a rate of 10 °C·min^-1^ for a period of 1 hour in a gas flow consisting of a 10% H_2_/Ar mixture (30 mL·min^-1^).

**CH_4_-TPSR, CO_2_-TPSR and isotope-TPSR**

All three types of TPSR experiments were performed identically. Typically, 50 mg of sample was loaded into a sample tube and pretreated at 300 °C for 0.5 h under He atmosphere, then the temperature was lowered to 50 °C and the gas was switched to CH_4_ or CO_2_. Then, temperature raised to 800 °C at a heat rate of 10 °C·min^-1^, during which the tail gas was connected to the mass spectrum (MS, Pfeiffer Omnistar). When C^18^O_2_ (1%, balance Ar) gas is injected, we got the isotope-TPSR spectra of C^18^O_2_.

**Multi-pulse coupled online Mass spectra experiments**

The multi-pulse coupled online mass spectra experiments were carried out at 600 °C on Pt/CeO_2_ catalysts. 80 mg of catalyst diluted with quartz sand was loaded into the reaction tube. After warming up to 600 °C, the reaction was carried out in a DRM atmosphere for 3 h before the start of the pulse experiment. For the methane pulse experiment, A cycle consists of a 5-minute Ar (95 mL·min^-1^) purge and a 2-minute CH_4_ (5 mL·min^-1^) dissociation. Three such cycles were defined as one step. By the difference between CO_2_ (5 mL·min^-1^) and H_2_S (10 ppm) at the step position, we obtained two sets of comparison experiments. For the carbon dioxide pulse experiments, passing H_2_S (10 ppm) in the middle of the two steps gave us the expected experimental results.

**In situ Raman spectra**

In situ Raman spectra of the samples were acquired by a LABRAM HR EVO spectrometer. The catalysts were excited with a 532 nm laser in the range of 50-2000 cm^-1^. 20 mg of catalyst was loaded into an in-situ cell (Linkam CCR1000) and pretreated with an N_2_ atmosphere at 300 °C for 0.5 h. Then, temperature was raised from 300 °C to 800 °C with a heating rate of 10 °C·min^-1^. Immediately after that, Spectra were collected every 10 min under the target gas stream.

**Carbon and sulfur contents analyzer**

The carbon and sulfur contents in the pretreated catalysts were measured by LECO CS 600CR analyzer. The samples were burned in pure oxygen to obtain CO_2_ and SO_2_, and the carbon and sulfur contents of the catalyst can be obtained by determining the CO_2_ and SO_2_ concentration by infrared, respectively.

**Quasi-in situ X-ray absorption spectroscopy (XAS) experiments.**

Quasi-in situ XAS experiments were conducted at the BM23 beamline of the European Synchrotron Radiation Facility (ESRF) during the experiment MA-6128. The X-ray beam, generated by a two-pole wiggler, was monochromatized using a Si (111) Double Crystal Monochromator. A double mirror with Rh coating was set at 2.2 mrad for harmonic rejection. Three ion chambers I_0_, I_1_ and I_2_ were employed to measure incident beam and signal transmitted by the sample and by the reference, respectively. I_0_ was filled with 80 mbar of Kr while I_1_ and I_2_ with 380 mbar of Kr and the three chambers pressure was top up to 2 bar in He. Pt L3-edge spectra of the sample were measured in transmission mode while the spectrum of Pt foil was simultaneously measured on I_2_ for energy calibration and alignment. For the XAS measurements, fresh sieved (HERE REPORT THE SIEVE FRACTION) catalysts (~75 µm, ~20 mg) were packed into a quartz capillary with an inner diameter of 2 mm and secured in place with quartz wool. The tube was 0.1 mm thick. The sample was ground and mixed with boron nitride and was pressed into a pellet with a diameter of 4 mm. XAS data were energy calibrated and aligned, background subtracted, and normalized to edge jump with the Athena software from the Demeter package.

**In situ TEM experiments.**

In situ TEM experiments were conducted at JEM-ARM200F with an in situ TEM gas-phase holder (SHW-MSK-PKG). Prior to experiments, the sample was encapsulated into a chip (EAT-33AA-10). In situ treatment was performed at 400 °C in 51 Torr N_2_ or CO_2_.

**In situ NAP-XPS experiments.**

In situ NAP-XPS experiments were performed with a lab-based NAP-XPS system (ESCALAB 250Xi, Al Kα X-ray source at 50 W). The powder sample was pressed into a smooth sheet and was fixed on a special sample table that can be heated to 600 °C during reaction. First, evacuate the reaction chamber to 1×10^-6^ mbar. Subsequently, introduce an atmosphere of N_2_ or CO_2_/N_2_ (1:5) at a total flow rate of 60 mL·min^-1^.

**Density functional theory (DFT) calculations**

The Vienna ab initio Simulation Package (VASP) was utilized to conduct first principles calculations within the framework of density functional theory (DFT).^[3, 4]^ The interaction between ions and electrons was characterized using the projector-augmented wave (PAW) method.^[5]^ The Perdew-Burke-Ernzerhof (PBE) functional, a generalized gradient approximation (GGA), was employed to account for exchange-correlation effects of electrons.^[6]^ Additionally, the DFT-D3 method, proposed by Grimme et al, was utilized to correct for van der Waals interactions.^[7]^ The climbing image nudged elastic band (Cl-NEB) method was used to search for reactive transition states.^[8]^ All files were visualized by VESTA program.^[9]^ Specifically, The CeO_2_ (111) surfaces were simulated using a three-layer slab repeated in a 4 × 4 surface unit cell, with a total of 48 cerium atoms and the corresponding stoichiometric ratio of oxygen atoms. The bottom layer was fixed, while the top two layers and adsorbent were allowed to relax. To prevent periodic disruptions, a vacuum layer thickness of 25 Å was set for each slab. A uniform energy cutoff of 400 eV was selected for all calculations. The convergence criterion for energy and force were set at 10^-5^ eV and 0.03 eV/Å, respectively. It was important to note that all calculations considered the spin polarization case. Due to the localized nature of the 4f electrons at Ce^3+^, calculations were carried out using the DFT + U method, where U representing the Hubbard parameter that accounts for double occupation of the 4f orbital.^[10]^ The optimal Hubbard parameter values determined by the DFT + U calculations were U_eff_ = 5 eV.^[11]^ A 2 × 2 × 1 Monkhorst-Pack k-point mesh was utilized for Brillouin zone integration. The adsorption energy (E_ads_) was defined by the following equation:

$$\text{E}_{\text{ads}}\text{ = }\text{E}_{\text{slab+g}}\text{ - }\text{E}_{\text{slab}}\text{ - }\text{E}_{\text{g}}$$

Where E_salb+g_ represents the total energy of the adsorption model, E_slab_ and E_g_ represent the total energies of corresponding slab and adsorbed species, respectively. The convergence criterion for energy and force was set at 10^-7^ eV and 0.03 eV/Å, respectively.

The Gibbs free energy change (ΔG) for each reaction step was determined by:

Δ*G*=Δ*E*+Δ*ZPE*-*T*Δ*S*

where E is the energy of the overall structure obtained using DFT calculations, ZPE is the zero−point energy of the substance, and S is the vibrational entropy of the substance, and T is the temperature (873.15K).

3. Figures and Tables


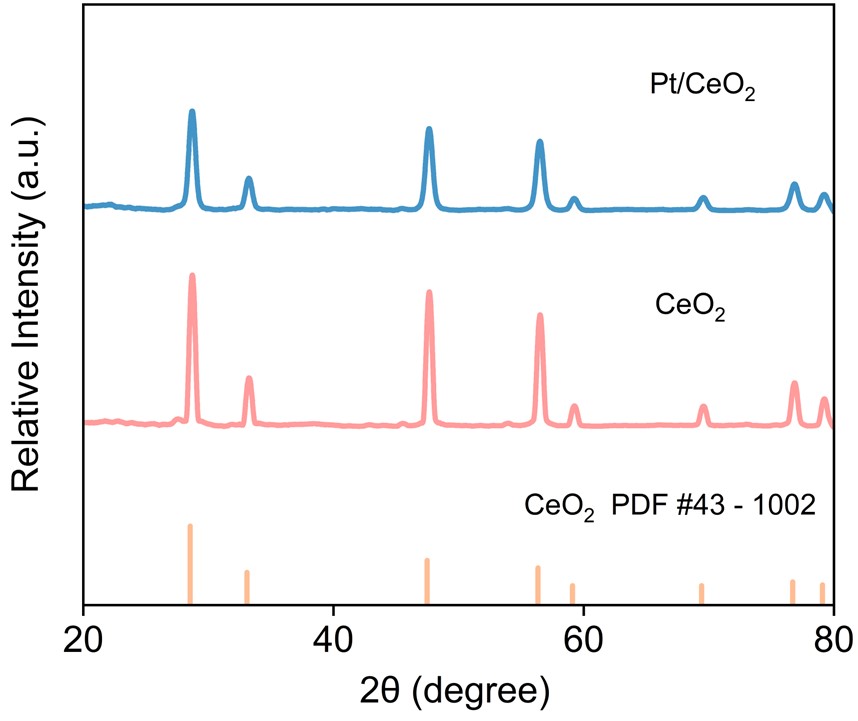


Figure S1. The X-ray diffraction patterns of the newly prepared catalysts.


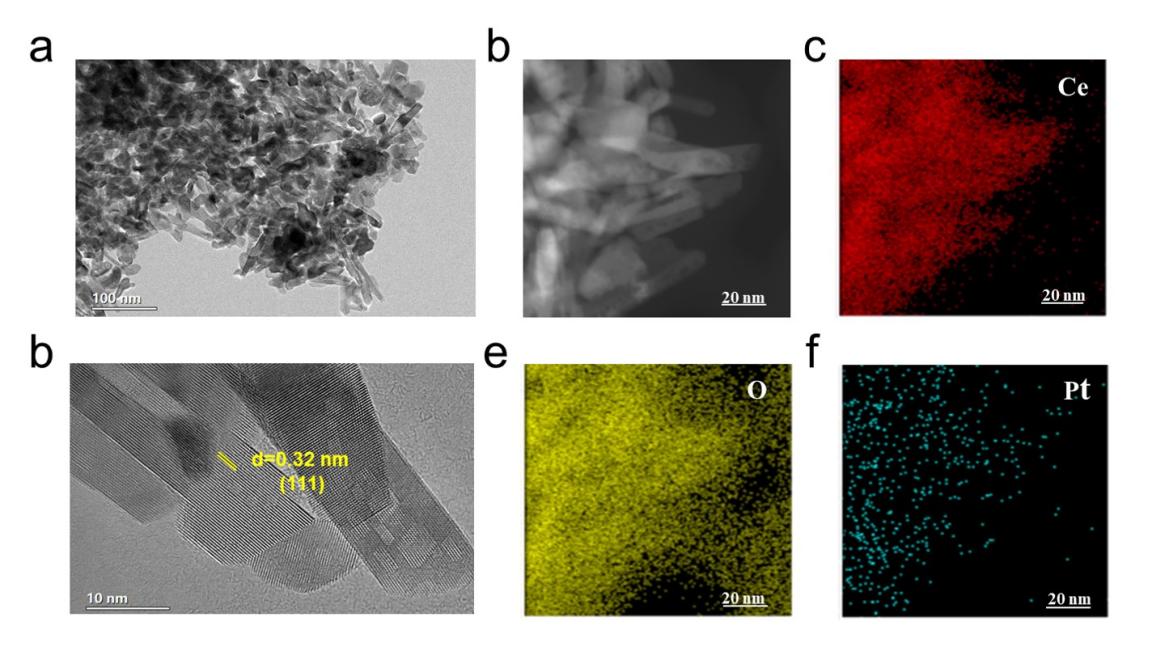


Figure S2. HR-TEM images of calcined Pt/CeO_2_ catalyst.


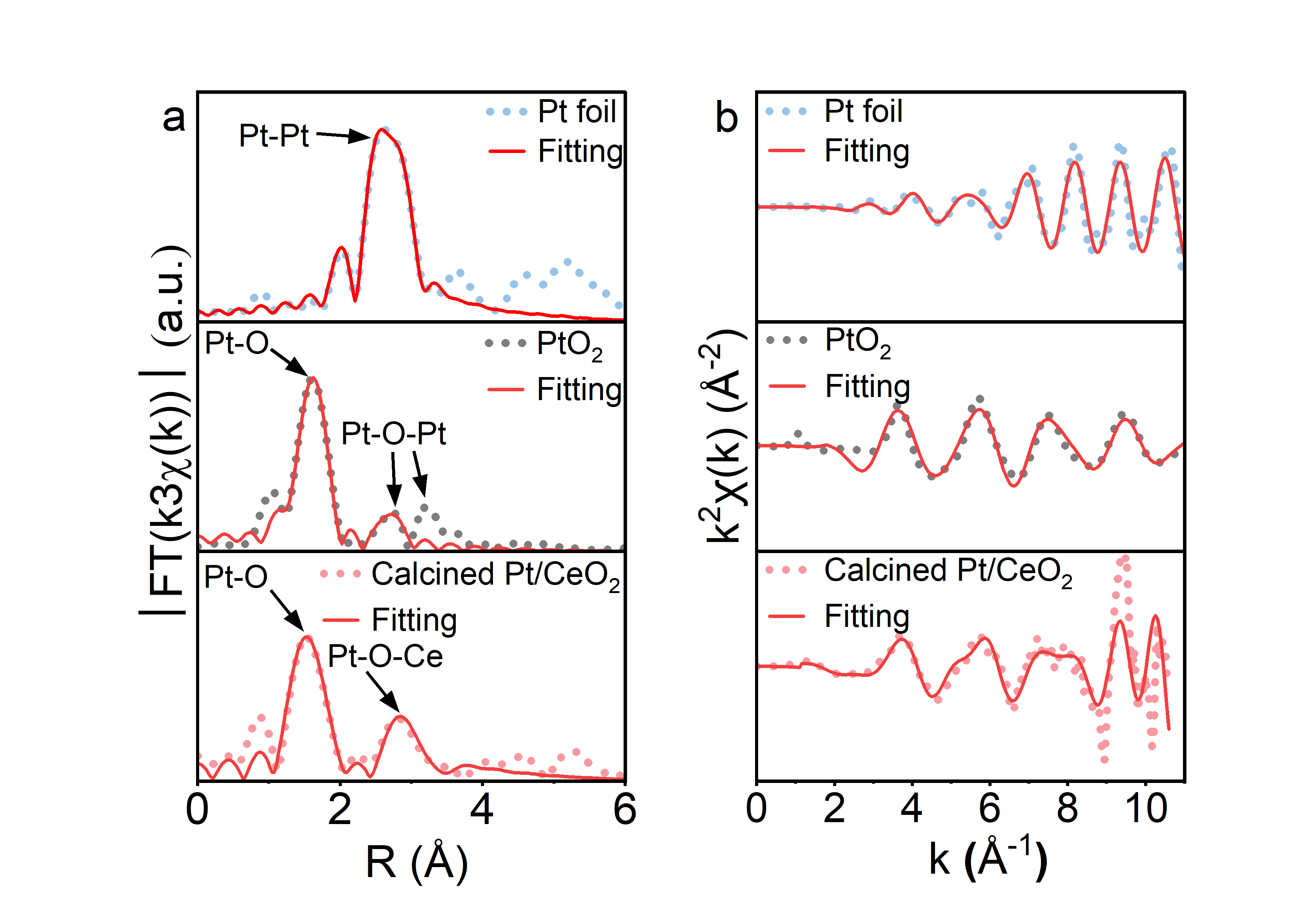


Figure S3. EXAFS and fitting curve in (a) R-space and (b) k-space of the Pt L_3_-edge of calcined Pt/CeO_2_, Pt foil, and PtO_2_ references.


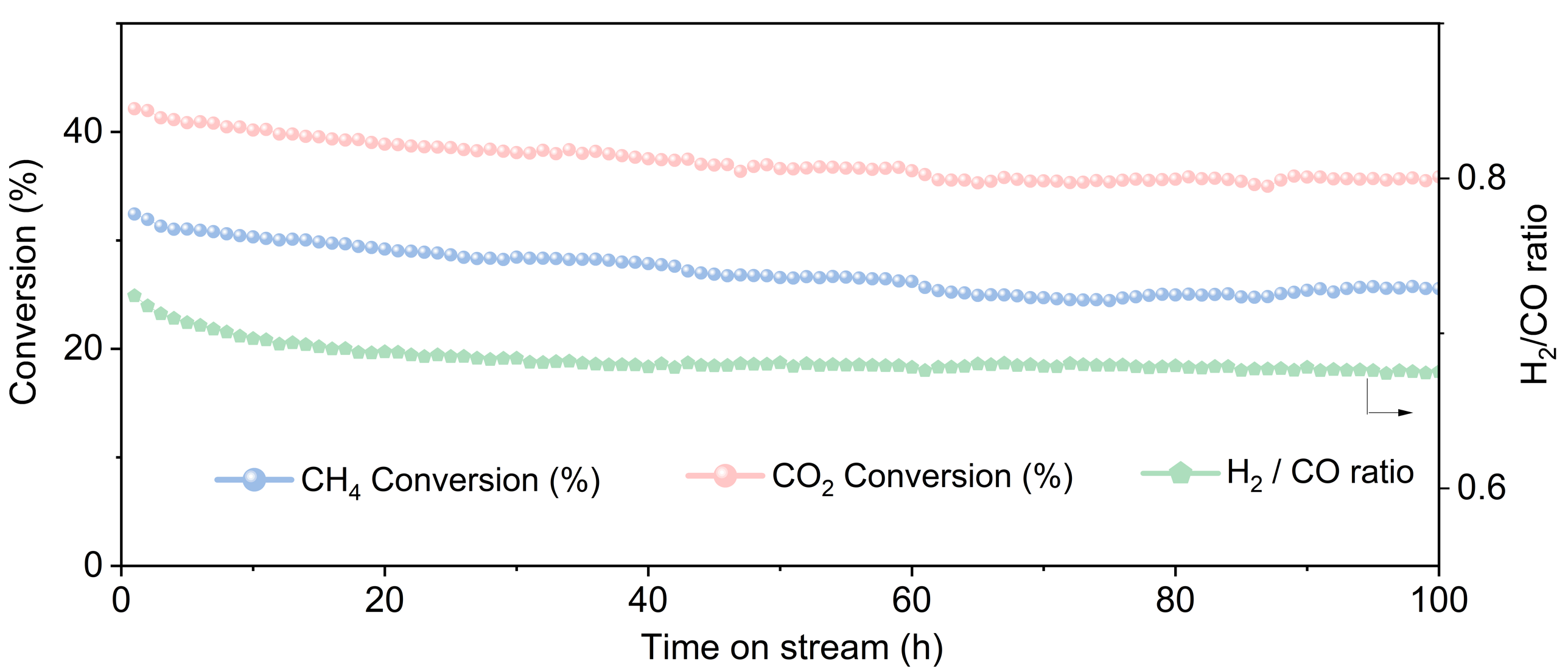


Figure S4. Conversion and H_2_/CO ratio of Pt/CeO_2_ catalyst with time on stream of 100 h. The DRM test was carried out at 600 °C with WHSV of 50,000 mL·g_cat_^–1^·h^–1^.


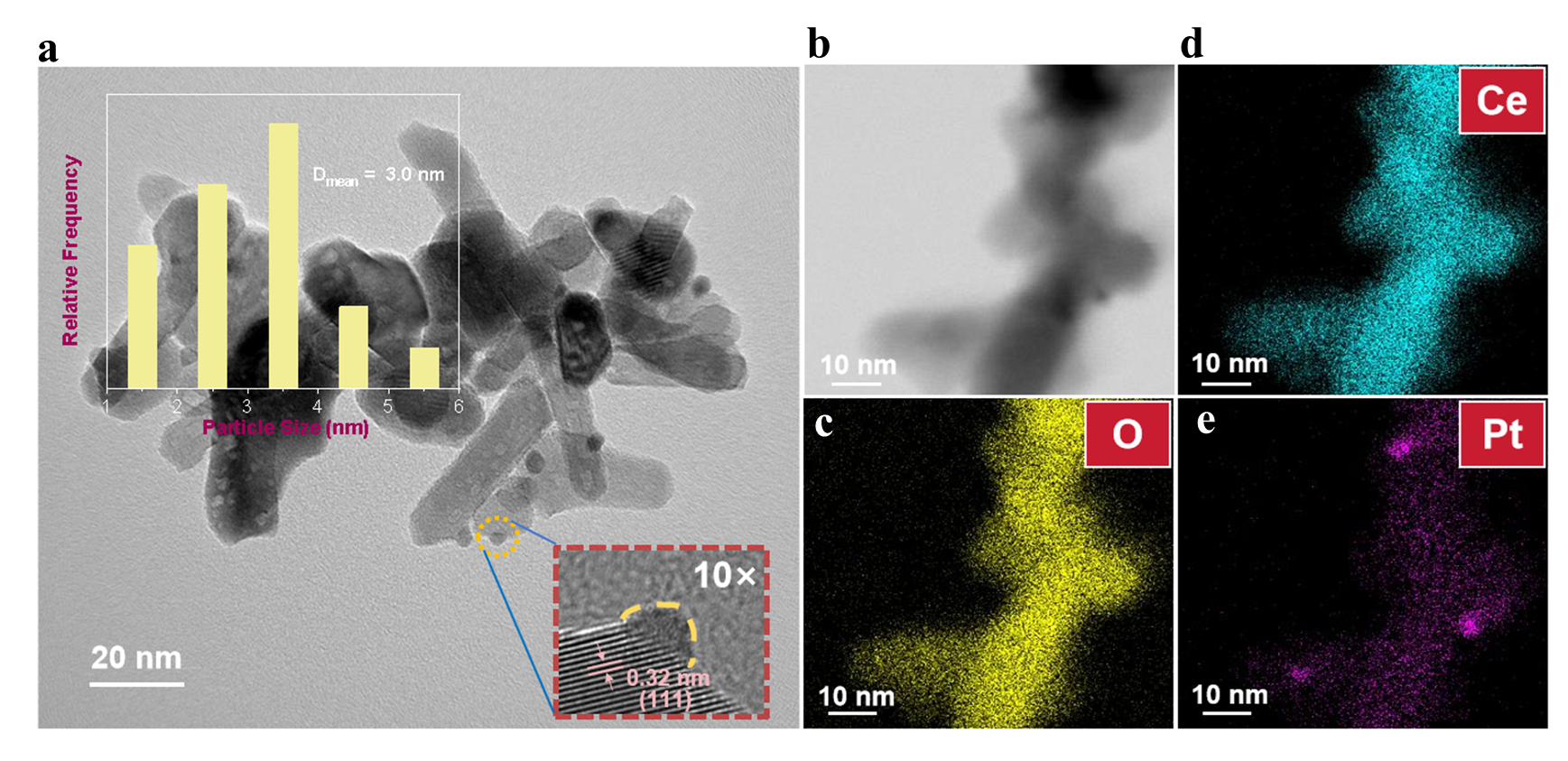


Figure S5. (a-b) HR-TEM images, the inset on the left shows the particle size distribution (Number of particles: 75) and the inset on the right shows the zoomed-in labelled section, (b) HAADF-STEM images, (c-e) Elemental mapping of Pt/CeO_2_ catalyst after 20 h DRM reaction which was carried out at 600 °C with GHSV of 50,000 mL·g_cat_^–1^·h^–1^.


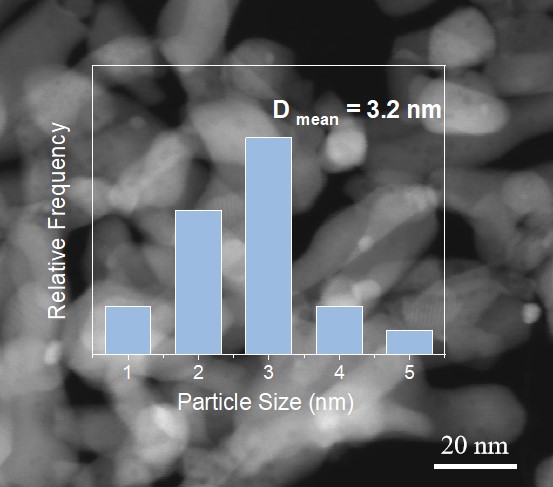


Figure S6. HAADF-TEM images (inset: particle size distribution (Number of particles: 75)) of Pt/CeO_2_ catalyst after 100 h DRM reaction which was carried out at 600 °C with GHSV of 50,000 mL·g_cat_^–1^·h^–1^.


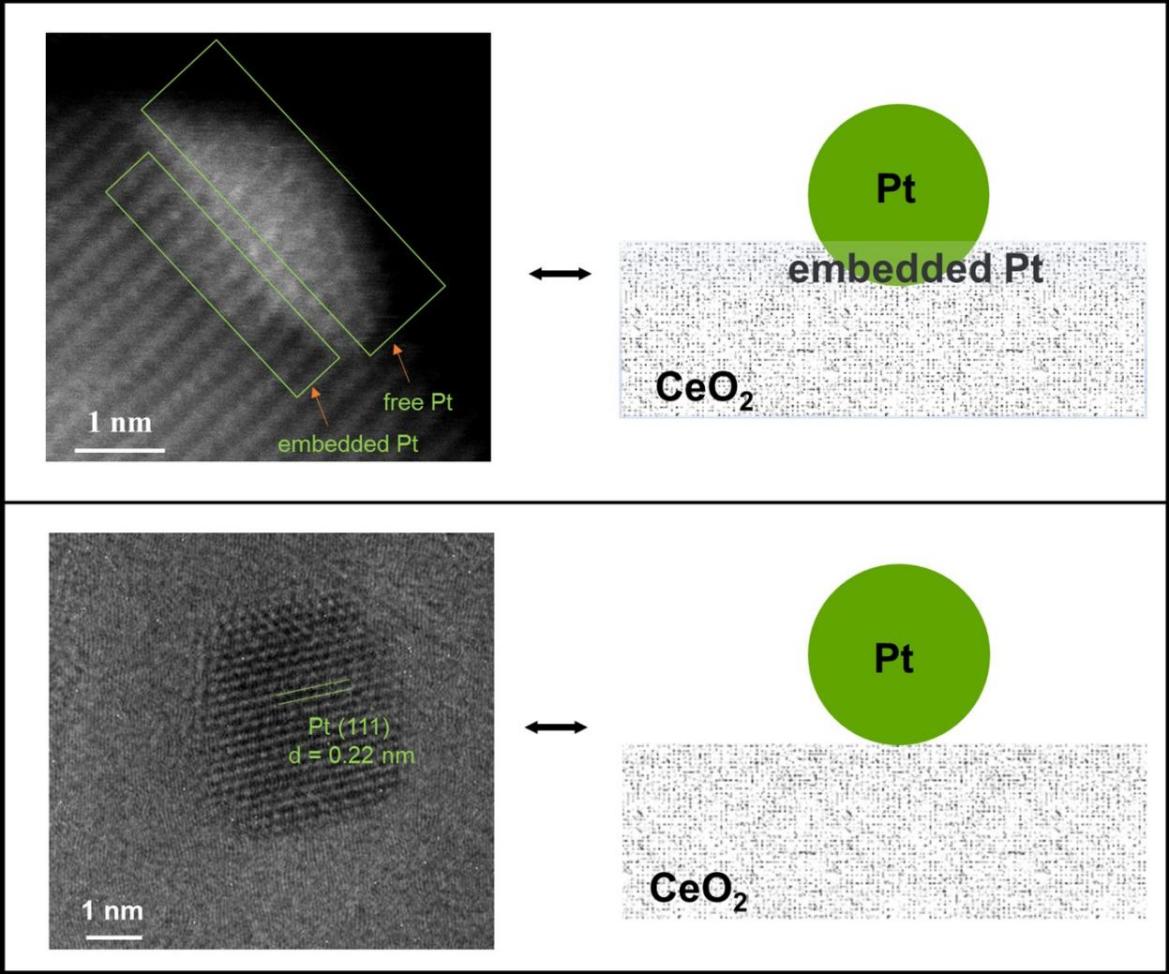


Figure S7. AC-HAADF-STEM image of Pt/CeO_2_ catalyst after 3h DRM reaction (up) and HR-TEM image of Pt/CeO_2_-r catalyst (down).


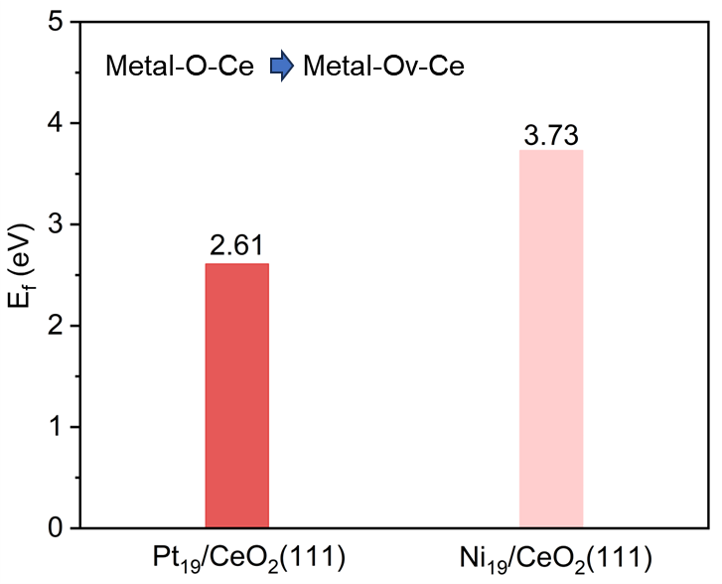


Figure S8. Calculated DFT simulations of the vacancy formation energy (E_f_) of Pt_19_/CeO_2_(111) and Ni_19_/CeO_2_(111).


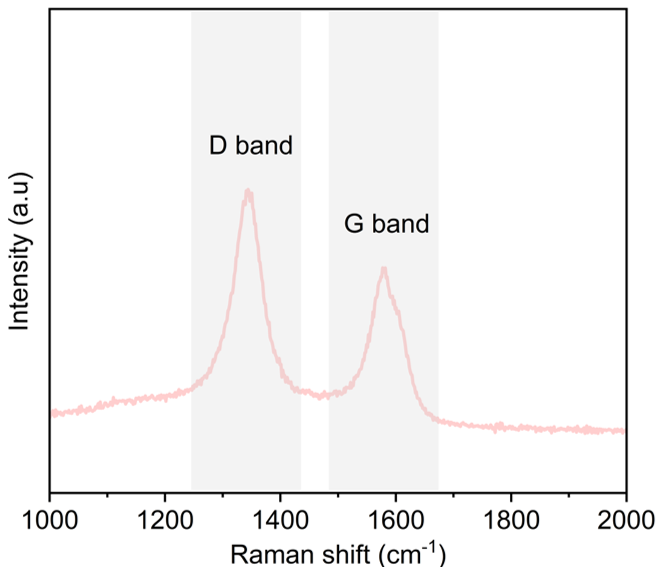


Figure S9. Raman profiles of spent Ni/CeO_2_ catalyst after 10 h reaction without H_2_S.


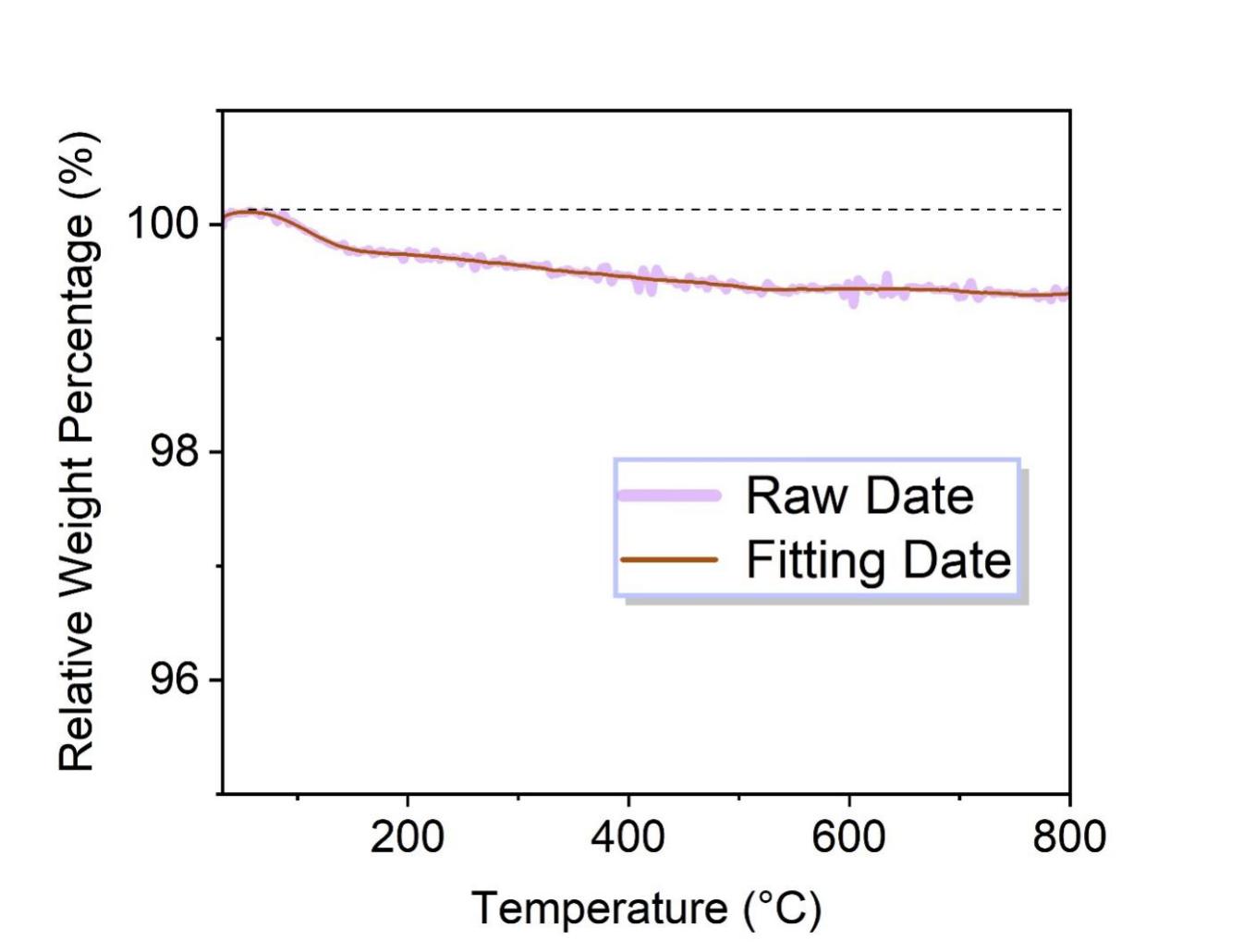


Figure S10. TG profiles of spent Pt/CeO_2_ catalyst after 100 h reaction.


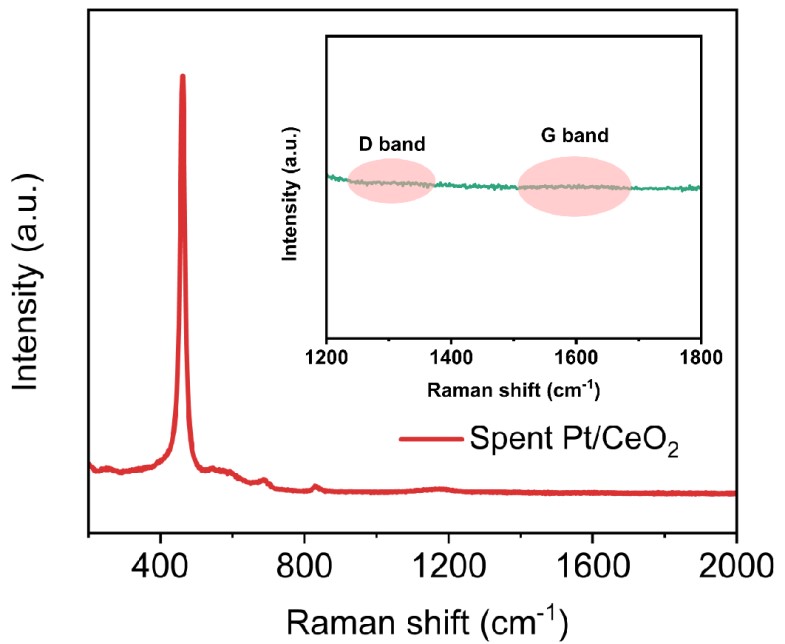


Figure S11. Raman profiles of spent Pt/CeO_2_ catalyst after 100 h reaction (inset plot shows the signal within the characteristic peak range of carbon).


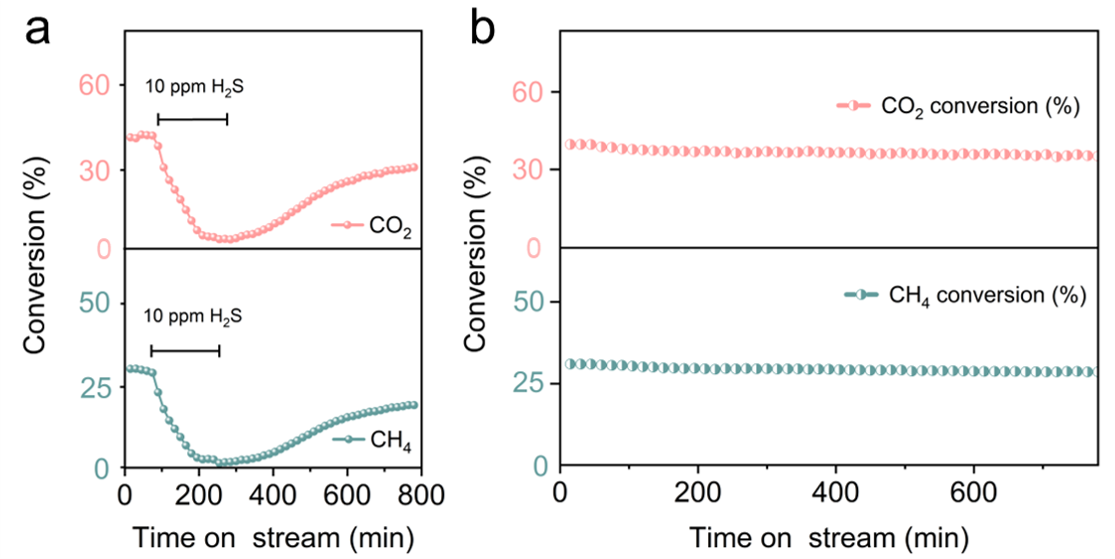


Figure S12. CH_4_ and CO_2_ conversion of Pt/CeO_2_ under (a) H_2_S poisoning for 3 hours and H_2_S shutdown for 9 hours, (b) Stability testing of DRM without H_2_S was conducted for 13 hours. The reaction was carried out at 600 °C with WHSV of 50,000 mL·g_cat_^–1^·h^–1^.


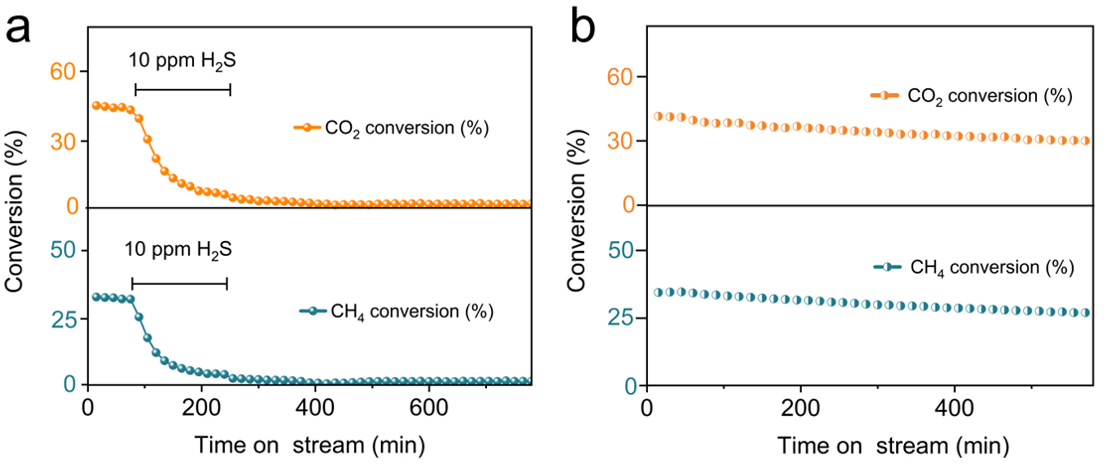


Figure S13. CH_4_ and CO_2_ conversion of Ni/CeO_2_ under (a) H_2_S poisoning for 3 hours and H_2_S shutdown for 9 hours, (b) Stability testing of DRM without H_2_S was conducted for 10 hours. The reaction was carried out at 600 °C with WHSV of 50,000 mL·g_cat_^–1^·h^–1^.


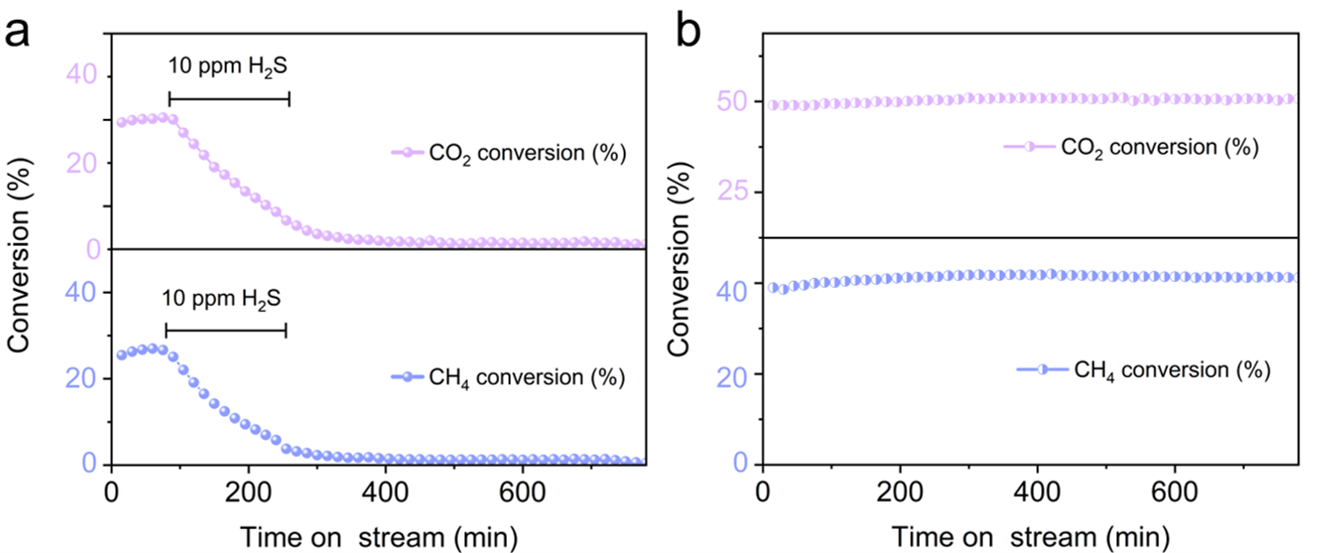


Figure S14. CH_4_ and CO_2_ conversion of Rh/CeO_2_ under (a) H_2_S poisoning for 3 hours and H_2_S shutdown for 9 hours, (b) Stability testing of DRM without H_2_S was conducted for 13 hours. The reaction was carried out at 600 °C with WHSV of 50,000 mL·g_cat_^–1^·h^–1^.


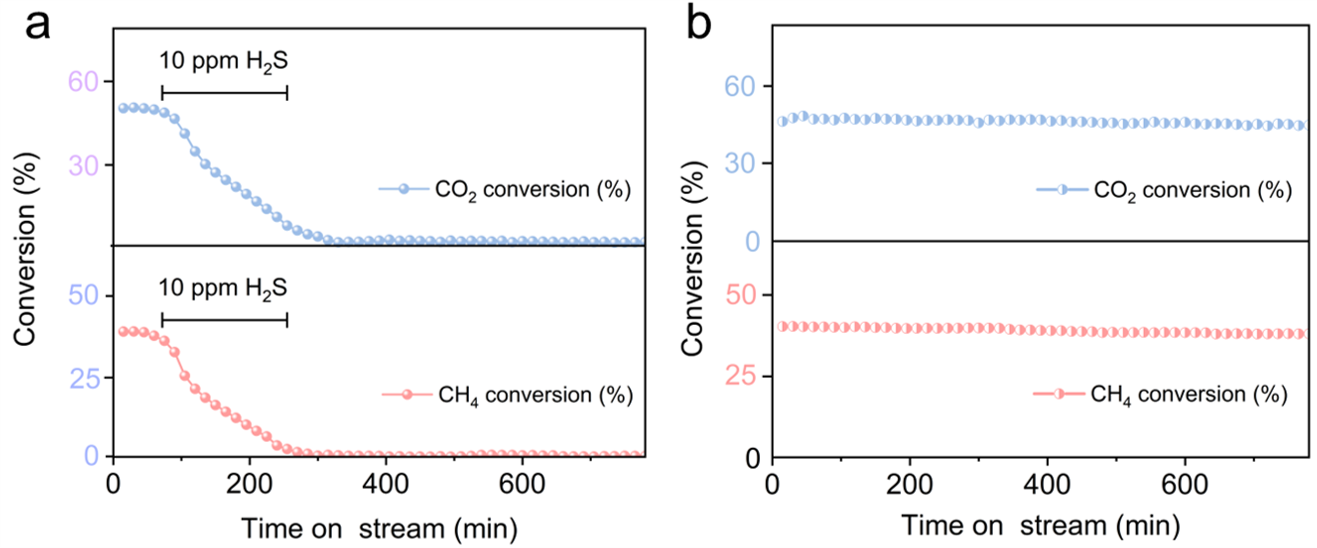


Figure S15. CH_4_ and CO_2_ conversion of Ru/CeO_2_ under (a) H_2_S poisoning for 3 hours and H_2_S shutdown for 9 hours, (b) Stability testing of DRM without H_2_S was conducted for 13 hours. The reaction was carried out at 600 °C with WHSV of 50,000 mL·g_cat_^–1^·h^–1^.


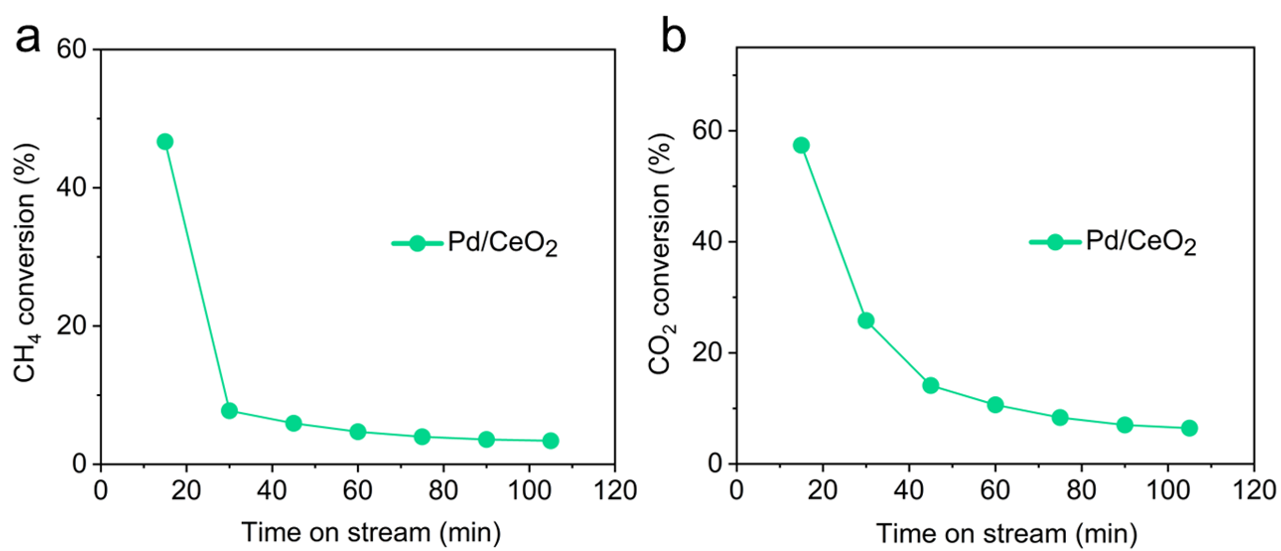


Figure S16. Conversion of (a) CH_4_ and (b) CO_2_ on Pd/CeO_2_ at 600 °C for DRM. Evaluated conditions: CH_4_/CO_2_/Ar=25/25/50 mL·min^–1^, WHSV=50,000 mL·g_cat_^–1^·h^–1^.

As for the Pd/CeO_2_ catalyst, we did not conduct hydrogen sulfide poisoning experiments on this catalyst due to the decrease of methane conversion from 56% to 3.4% in the DRM reaction in 2 h. The precise rationale behind this matter will not be addressed in this research as it falls outside the scope of our investigation.


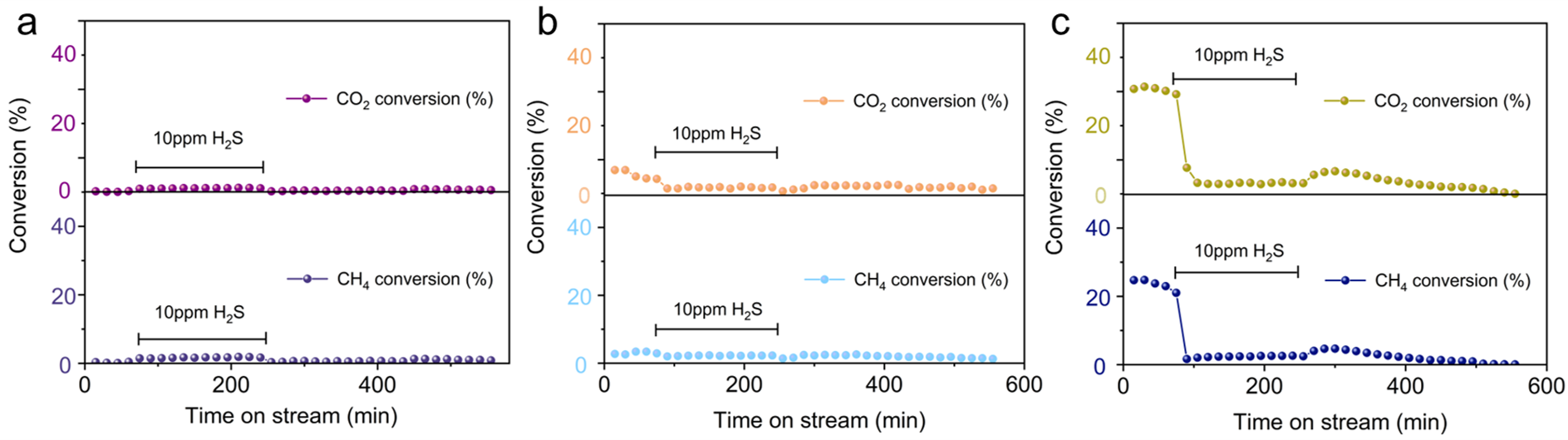


Figure S17. CH_4_ and CO_2_ conversion of (a) Pt/SiO_2_. (b) Pt/MgO. (c) Pt/γ-Al_2_O_3_ for H_2_S poisoning for 3 h and H_2_S shutdown for 3 h. The reaction was carried out at 600 °C with WHSV of 50,000 mL·g_cat_^–1^·h^–1^.


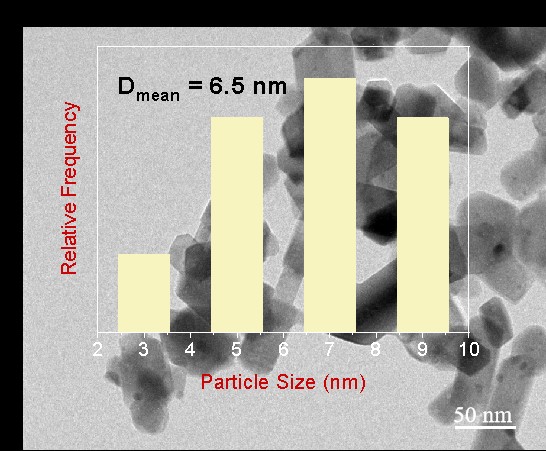


Figure S18. High-resolution TEM image of the fresh Pt/CeO_2_ catalyst after reduction. The inset shows the corresponding particle size distribution (number of particles counted: 105).

**
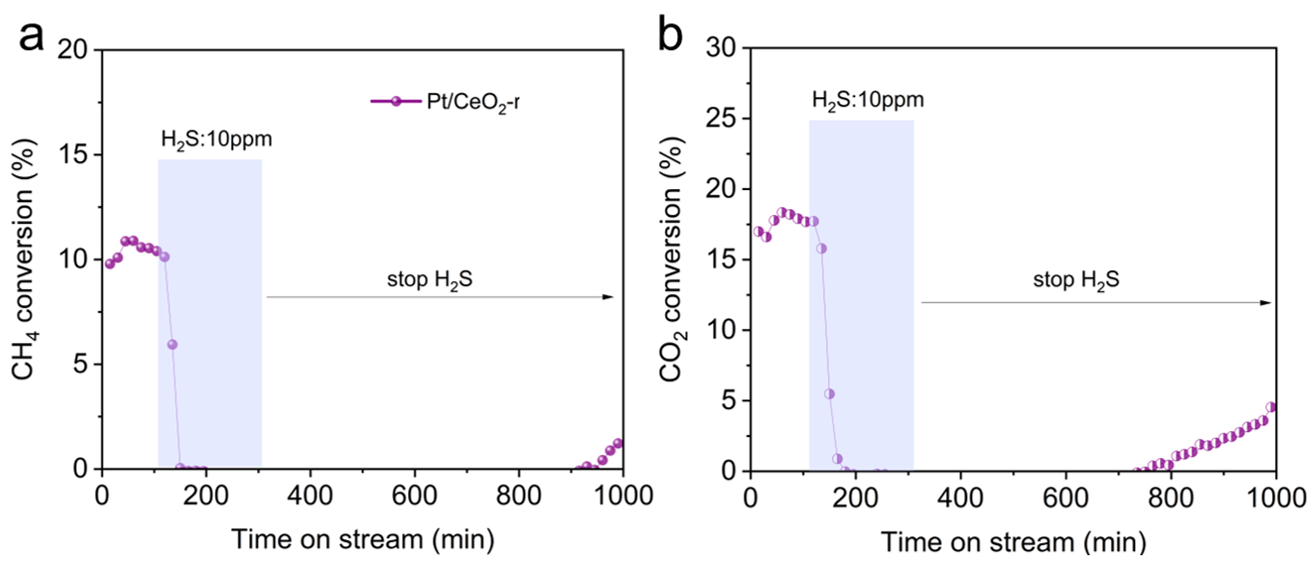
**

Figure S19. (a) CH_4_ and (b) CO_2_ conversion of reduced Pt/CeO_2_ after H_2_S poisoning for 3 h and H_2_S shutdown for 13 h. The reaction was carried out at 600 °C with WHSV of 50,000 mL·g_cat_^-1^·h^-1^. Data points with gaps signify unsuccessful conversions that did not result in any reaction products.


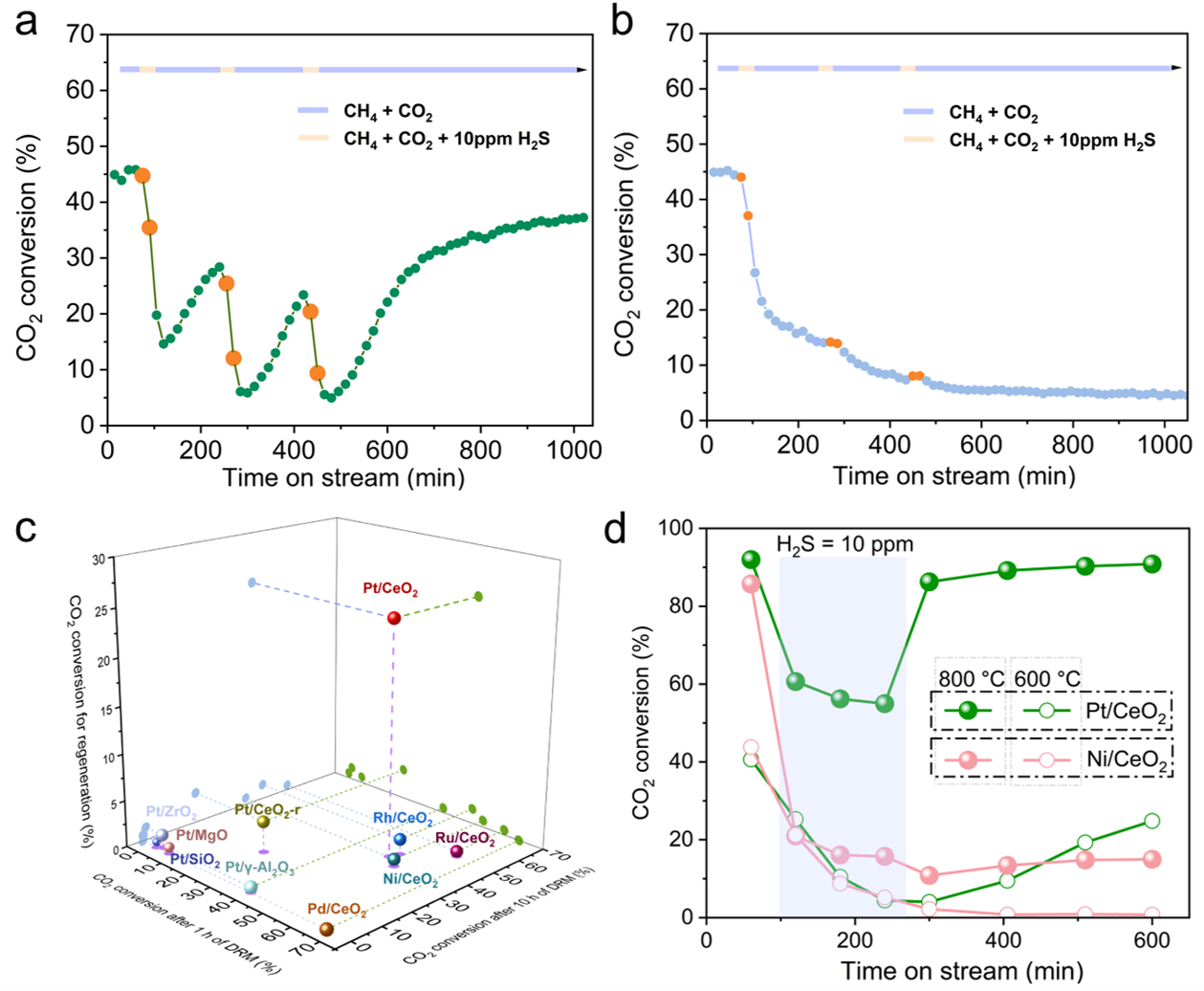


Figure S20. CO_2_ conversion with sequence poisoning of H_2_S over (a) Pt/CeO_2_ and (b) Ni/CeO_2_ catalysts at 600 °C. (c) Three-dimensional scatter image depicting the conversion of CO_2_ for various catalysts (X-axis: initial DRM activity within one hour, Y-axis: 10-hours DRM activity, and Z-axis: DRM activity after 6 hours of the self-recovery after H_2_S-poisoning). The DRM reaction was carried out at 600 °C with WHSV of 50,000 mL·g_cat_^–1^·h^–1^. The H_2_S concentration is 10 ppm in case of poisoning. (d) CO_2_ conversion of Pt/CeO_2_ and Ni/CeO_2_ catalysts in the presence or absence of H_2_S at 600 °C and 800 °C, respectively.


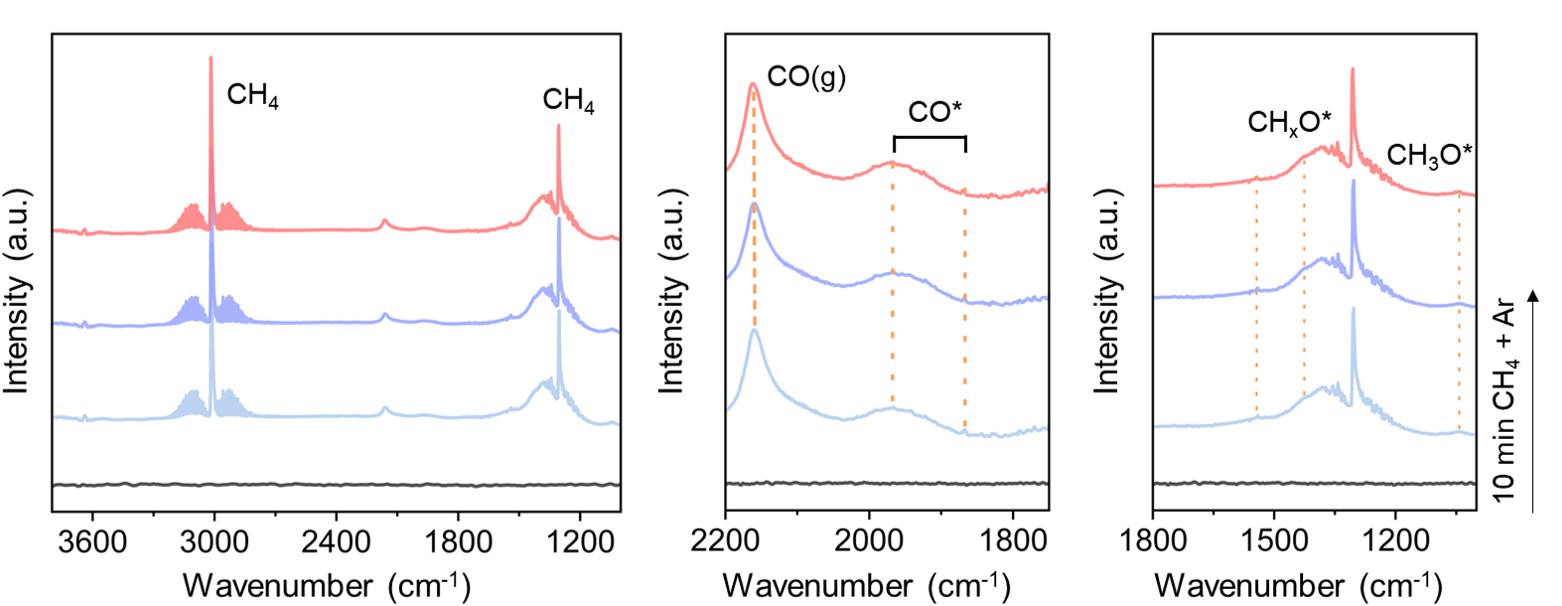


Figure S21. In situ DRIFTS spectra analysis of DRM mechanism on Pt/CeO_2_ after 20 h DRM reaction. CH_4_ was adsorbed at 600 °C for 10 min under 25% CH_4_/Ar flow after in-situ pretreatment and Ar purging, respectively.

In order to examine the active sites of methane and carbon dioxide on the surface of Pt/CeO_2_ catalysts, we have obtained corresponding results via Diffuse Reflectance Infrared Fourier Transform Spectroscopy (DRIFTS) and Density Functional Theory (DFT) simulations. Regarding the CH_4_ adsorption spectra, the triply degenerated antisymmetric stretching mode v_3_ and the 2-fold degenerated H-C-H deformation mode v_4_ signals of gas-phase CH_4_ were observed at 3018 cm^-1^ and 1305 cm^-1^, respectively.^[12]^ Upon activation of CH_4_ on the catalyst surface, CO (1800 to 2200 cm^-1^), CH_x_O* (1390 cm^-1^), CH_3_O* (1048 cm^-1^). As for the CO_2_ adsorption spectra, the peak signals of gas-phase CO_2_ were observed in the range of approximately 2230 to 2250 cm^-1^. When CO_2_ was chemisorbed on the catalyst surface, the signal peaks we detected for carbonate. From the figure shown, after activation of CH_4_ on the surface of the Pt/CeO_2_ catalyst it was observed that the signals generated by bridge-bonded CO on the Pt nanoparticles were located at 1968 cm^-1^ and 1868 cm^-1^.^[13, 14]^


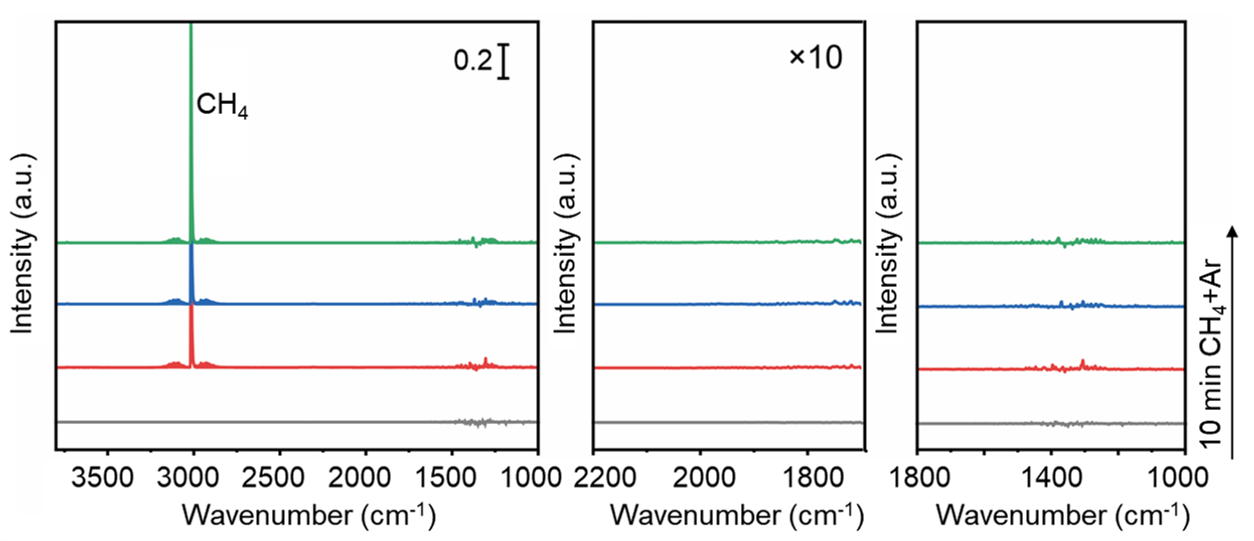


Figure S22. In situ DRIFTS spectra analysis of DRM mechanism on Pt/SiO_2_. CH_4_ was adsorbed at 600 °C for 10 min under 25% CH_4_/Ar flow after in-situ pretreatment and Ar purging, respectively.


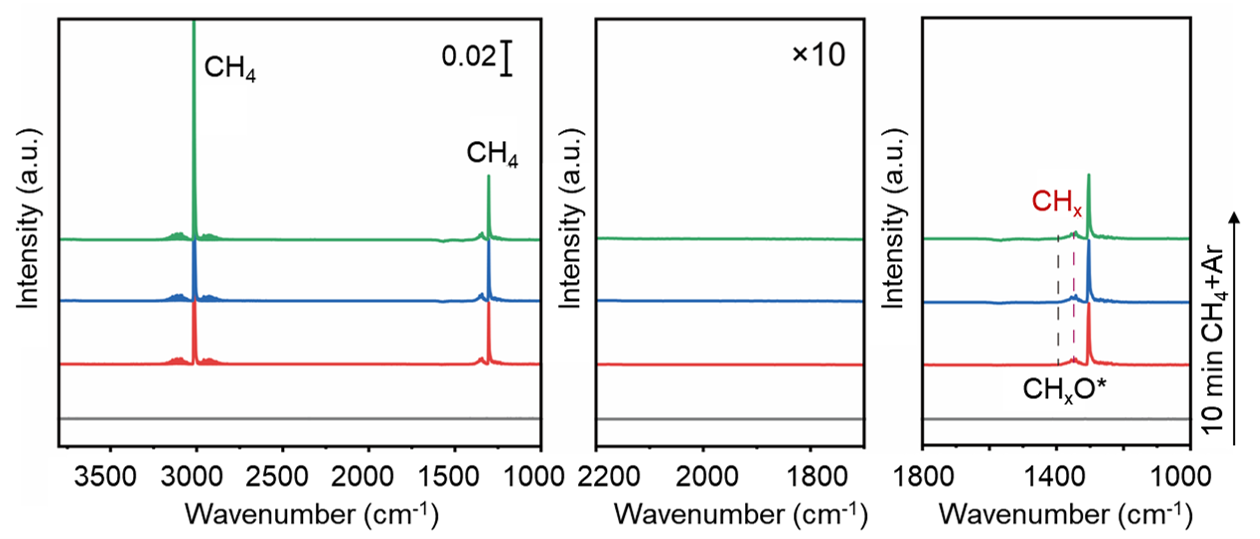


Figure S23. In situ DRIFTS spectra analysis of DRM mechanism on Pt/γ-Al_2_O_3_. CH_4_ was adsorbed at 600 °C for 10 min under 25% CH_4_/Ar flow after in-situ pretreatment and Ar purging, respectively.


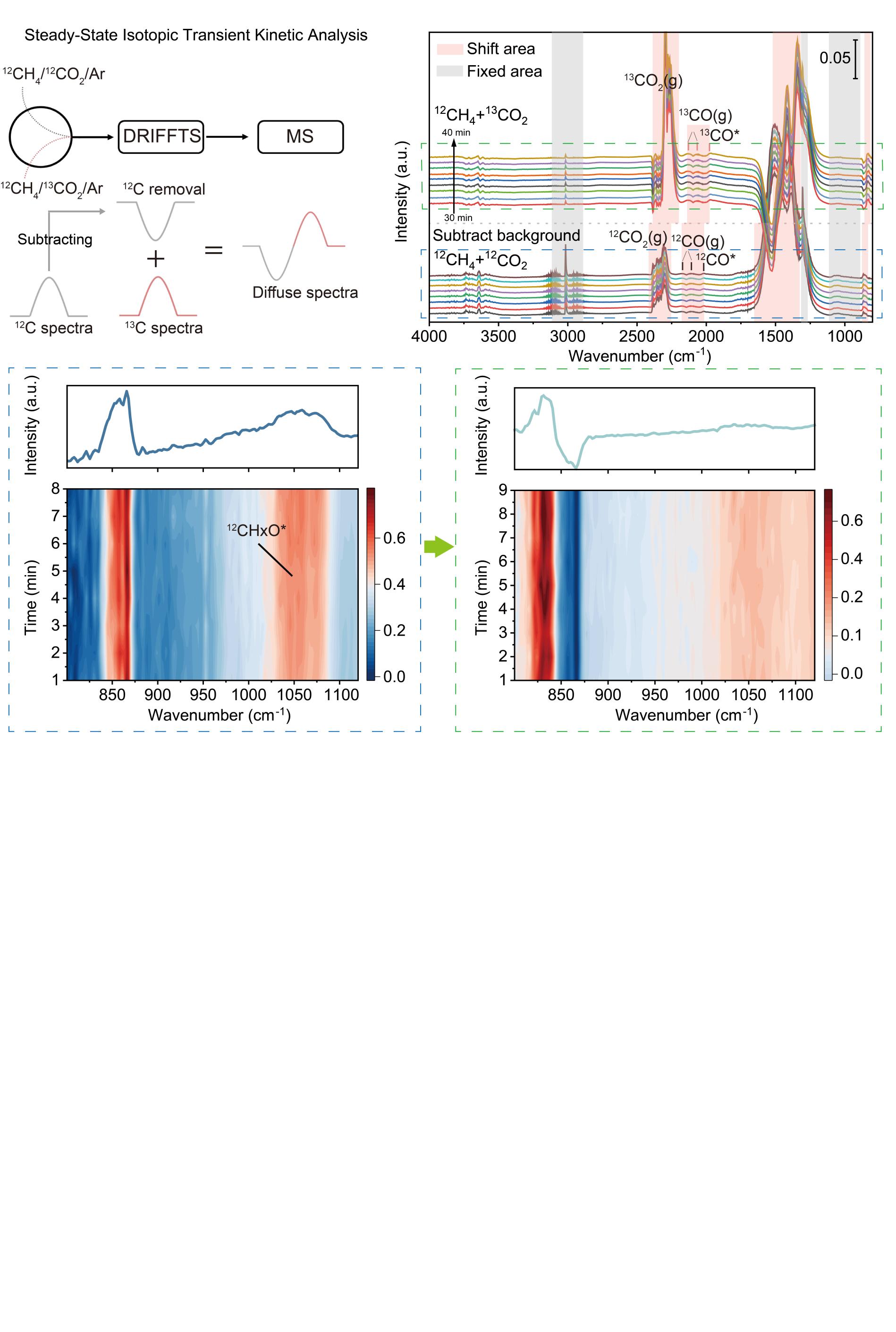


Figure S24. The operando DRIFTS-SSITKA spectra of DRM process over Pt/CeO_2_ after 20 h DRM reaction. The catalyst was introducing ^12^CH_4_/^12^CO_2_/Ar and shifted to ^12^CH_4_/^13^CO_2_/Ar at 600°C (flow rate = 20 ml min^-1^).

In the operando DRIFTS-SSITKA experiment of Pt/CeO_2_, after the catalyst surface reached a steady state under ^12^CH_4_/^12^CO_2_/Ar flow, the gas was switched to ^12^CH_4_/^13^CO_2_/Ar. Throughout this process, isotopic re-equilibration was observed only in the spectral regions associated with CO_2_ (marked as the shift area). In contrast, the region corresponding to CH_x_O* species showed no significant isotopic effect upon switching from ^12^CO_2_ to ^13^CO_2_ (marked as the fixed area). This indicates that methane undergoes an oxygen‑assisted pathway (CHₓ* + O* → CHₓO*) on the Pt/CeO_2_ surface and that CHₓO* is not produced via CO_2_ hydrogenation (CO* + xH* → CHₓO*).


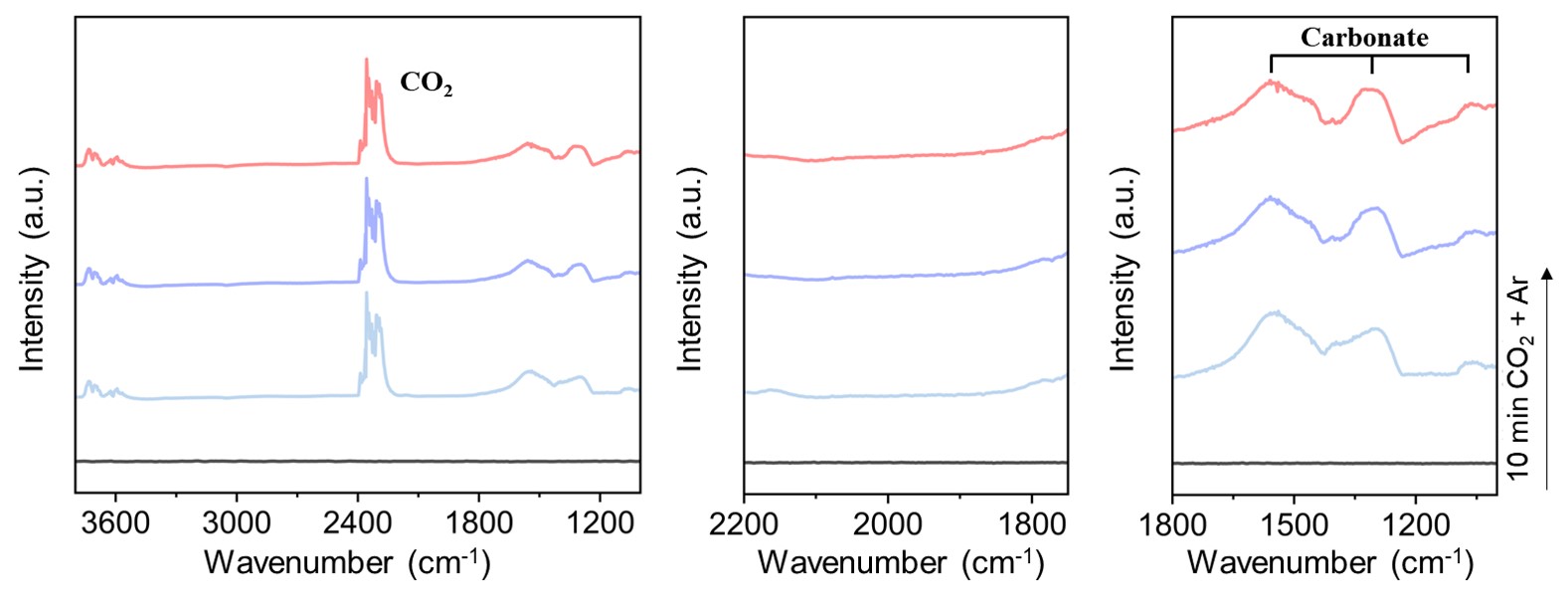


Figure S25. In situ DRIFTS spectra analysis of DRM mechanism on Pt/CeO_2_ after 20 h DRM reaction. CO_2_ was adsorbed at 600 °C for 10 min under 25% CO_2_/Ar flow after in-situ pretreatment and Ar purging, respectively.

In contrast, the adsorption of CO_2_ did not have signals from the adsorbed state of CO in addition to those from carbonates. This indicated that CH_4_ and CO_2_ were activated on Pt nanoparticles and CeO_2_, respectively, in the Pt/CeO_2_ catalyst.


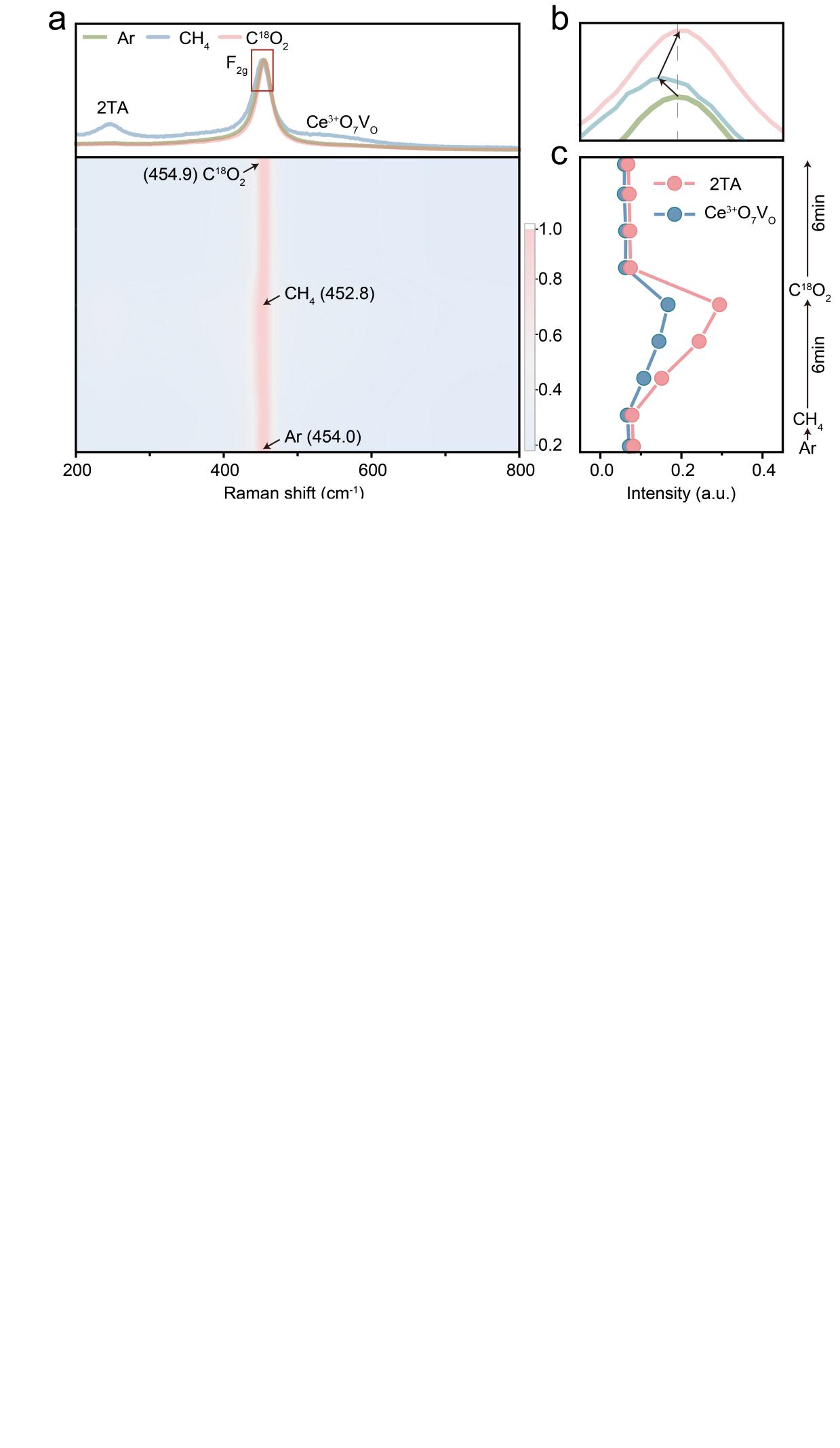


Figure S26. In situ Raman spectra analysis of DRM process over CeO_2_ at 600 °C. 25% CH_4_/Ar and 25% C^18^O_2_/Ar flow was introduced after in-situ pretreatment and Ar purging, sequentially.

To further verify this interpretation, isotope-Raman measurements were performed to clarify the correlation between surface species evolution and structural changes. The shift of the Ce–O vibrational signal (F2g mode) provides insights into the structural evolution of the catalyst surface. As shown on Figure S23, during reduction (dominated by CH_4_ activation), electron localization around Ce^3+^ and oxygen vacancies weakens the Ce–O bonds, leading to a red shift of the F2g mode. Conversely, the re-oxidation or oxygen vacancy healing process (dominated by C^18^O_2_ activation) results in a blue shift.


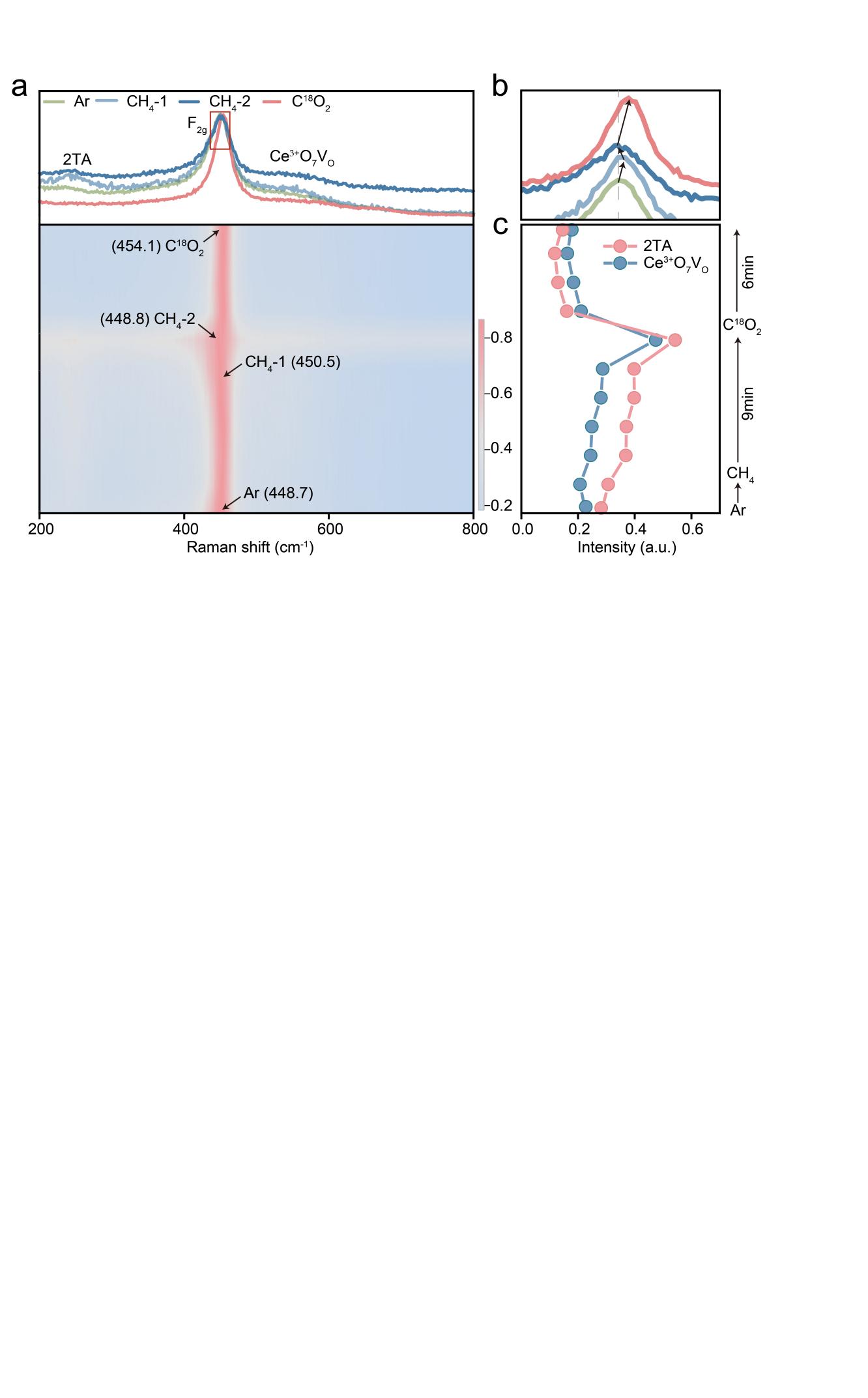


Figure S27. In situ Raman spectra analysis of DRM process over Pt/CeO_2_ at 600 °C. 25% CH_4_/Ar and 25% C^18^O_2_/Ar flow was introduced after 20h DRM reaction and Ar purging, sequentially.

For the Pt/CeO_2_ catalyst, the F2g mode initially exhibited a blue shift during the early stage of CH_4_ activation and returned to its original position after 6 minutes. When the atmosphere was switched to C^18^O_2_, a more pronounced red shift was observed compared with bare CeO_2_, suggesting that CH_4_ and CO_2_ are activated at separated sites—Pt-O-Ce and CeO_2_, respectively—which is consistent with the DRIFTS-SSITKA results.


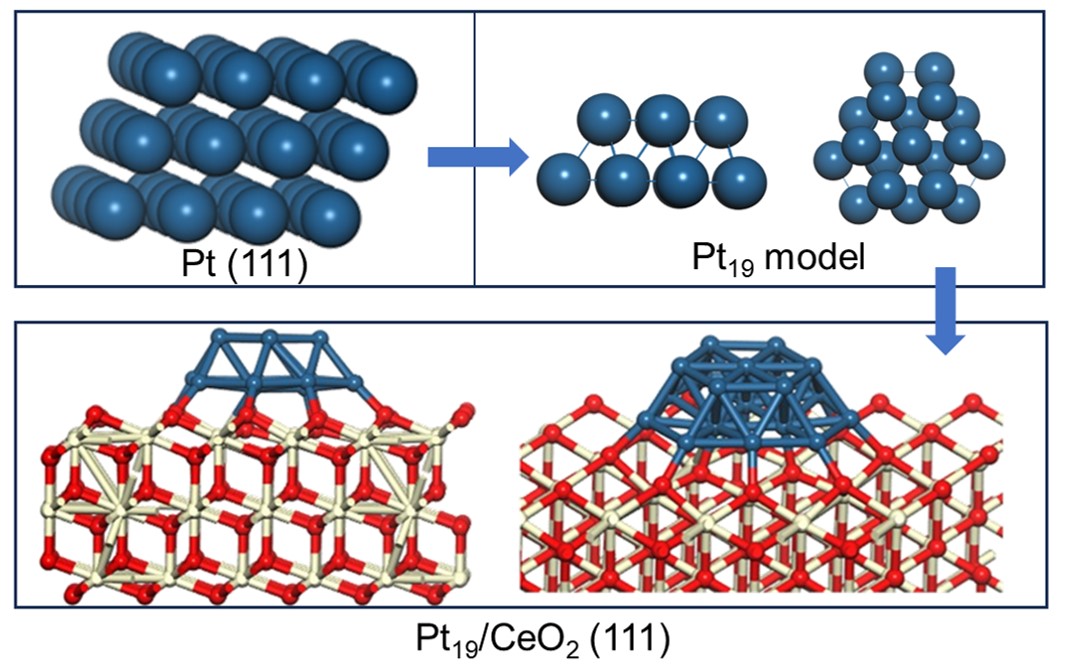


Figure S28. Schematic diagram of Pt_19_/CeO_2_(111). Pt, navy; Ce, beige; O, red.


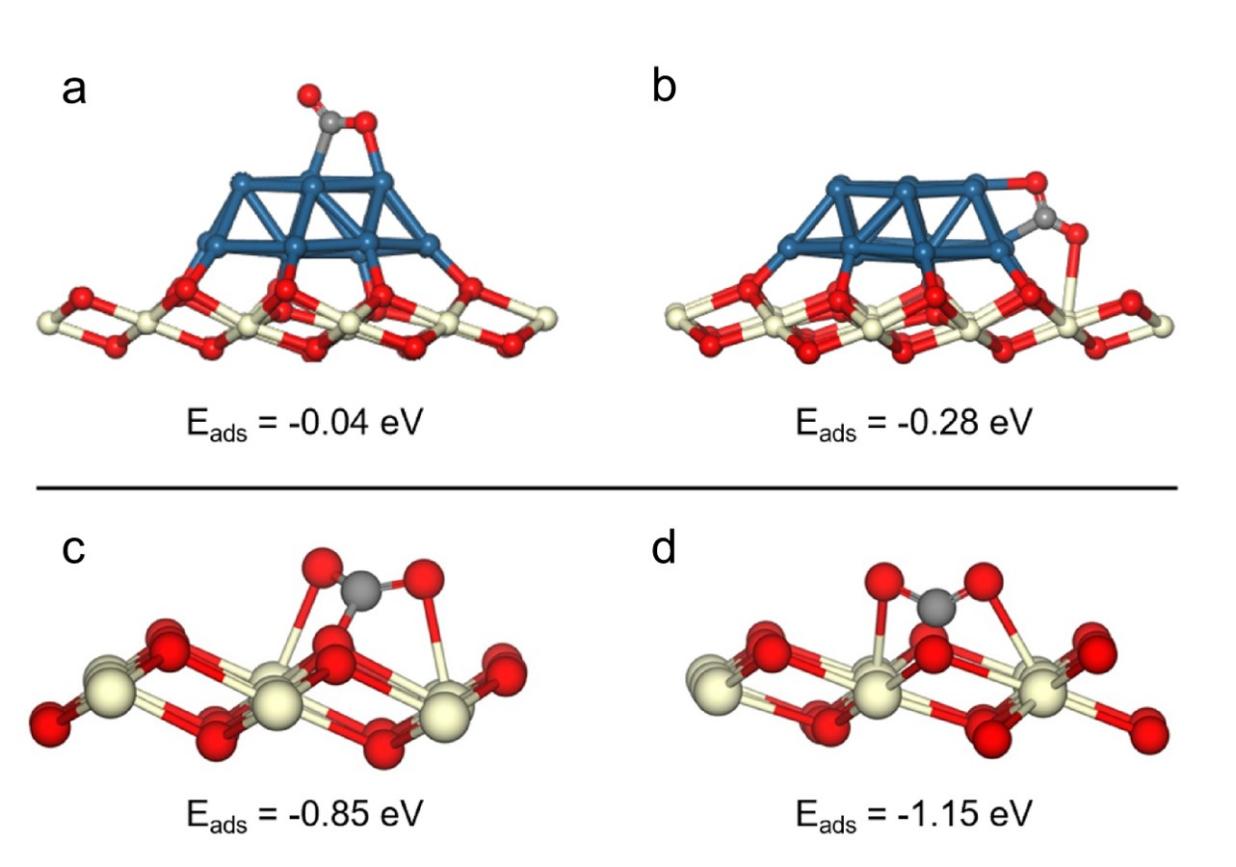


Figure S29. Optimized CO_2_ adsorption model image. CO_2_ molecules adsorbed at (a) Pt_bottom_ site, (b) Pt_top_ site on the Pt/CeO_2_. CO_2_ molecules adsorbed at (c) clean cerium oxide and (d) cerium oxide surface oxygen vacancies. E_ads_ refers to the estimated CO_2_ adsorption energy. Pt, navy; Ce, beige; O, red; C, gray.


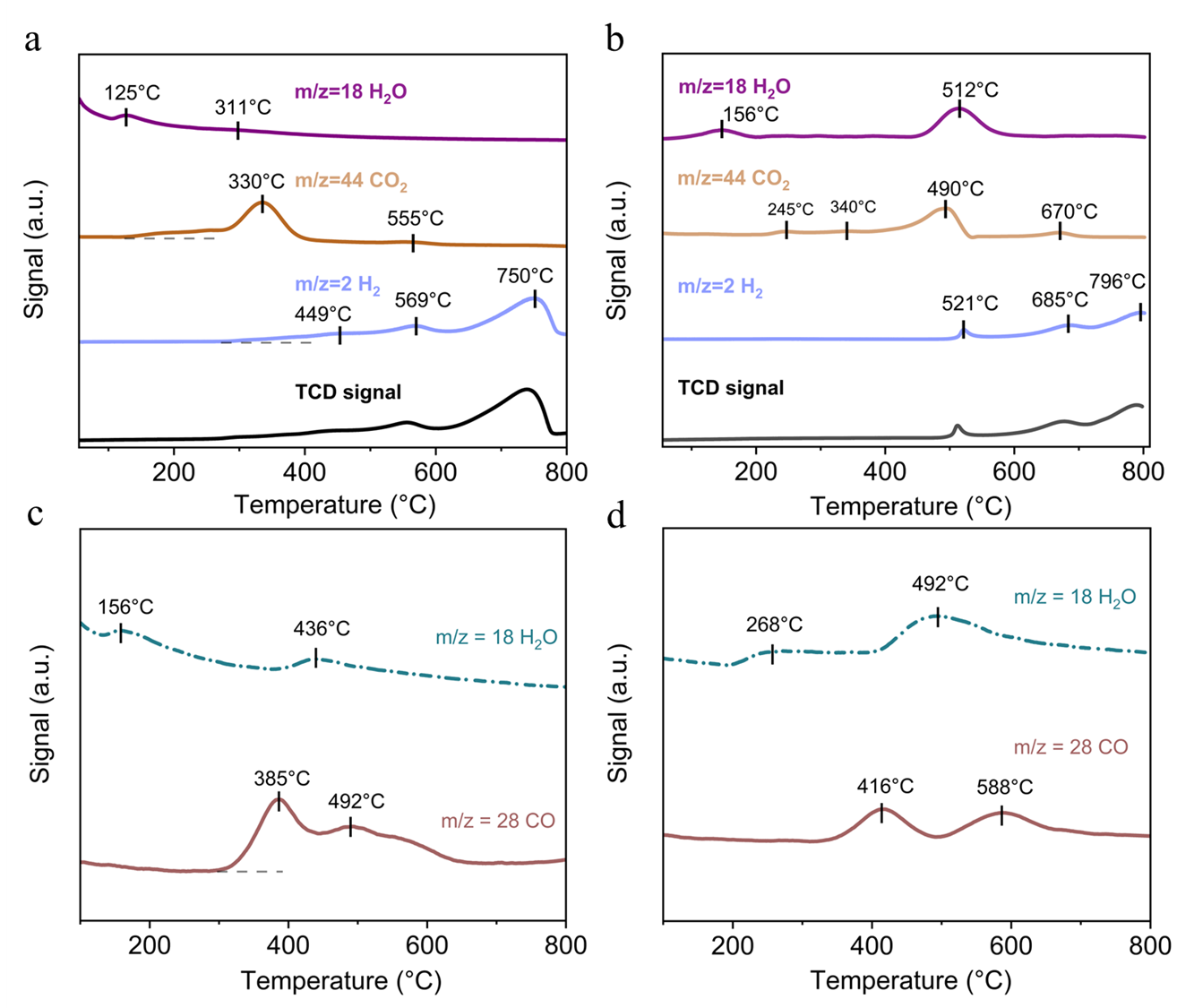


Figure S30. CH_4_-TPSR-MS profiles of (a) Pt/CeO_2_ fresh catalyst, (b) Pt/CeO_2_ catalyst after 3 h H_2_S treatment, CO_2_-TPSR-MS profiles of (c) Pt/CeO_2_ fresh catalyst, (d) Pt/CeO_2_ catalyst after 3 h H_2_S treatment.

Upon introduction of H_2_S, the TPSR results indicate that the activation of both CH_4_ and CO_2_ is affected. The activation temperature of CH_4_ over the Pt/CeO_2_ catalyst increased from 449 to 521 °C after H_2_S poisoning, while the activation temperature of CO_2_ increased from 385 to 416 °C. This shift reflects site blockage and suppressed oxygen exchange at the Pt–O–Ce interface caused by sulfur adsorption. Consequently, the presence of H_2_S leads to reduced Pt/CeO_2_ catalyst activity. Given the negligible DRM properties of pure CeO_2_ (Figure S28), complete deactivation of the catalysts in the presence of H_2_S may result from the competitive adsorption between the reactants and H_2_S or the continuous accumulation of S, which fully covers the Pt sites.


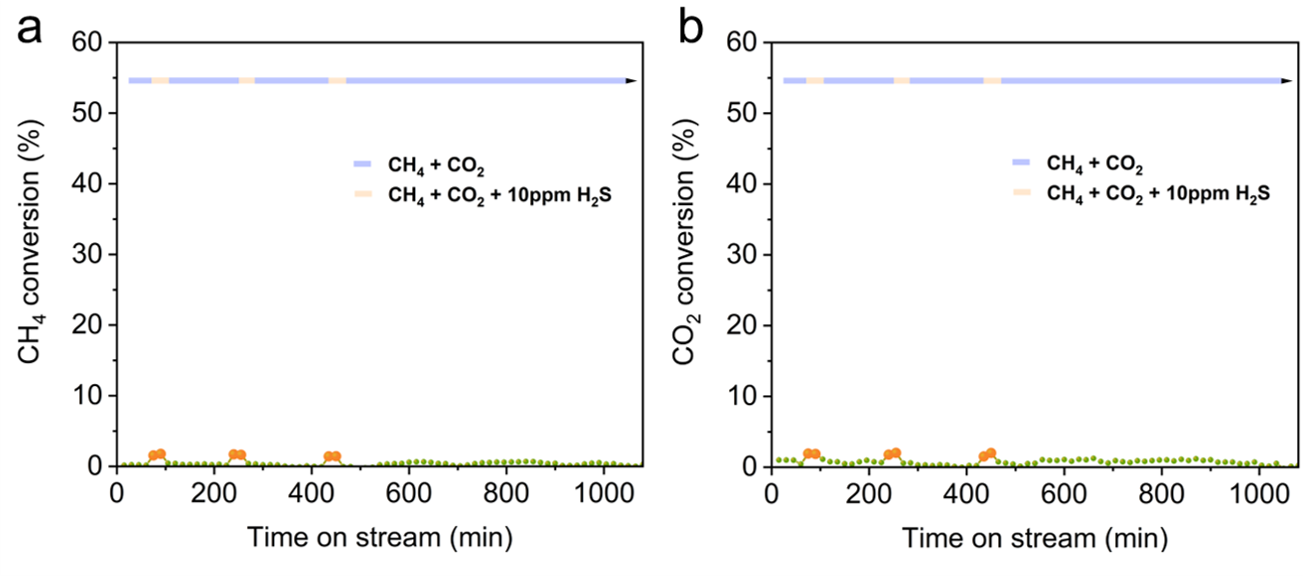


Figure S31. Cyclic poisoning self-recovery profiles of (a) CH_4_ conversion and (b) CO_2_ conversion of CeO_2_ under 600 °C with WHSV of 50,000 mL·g_cat_^–1^·h^–1^.


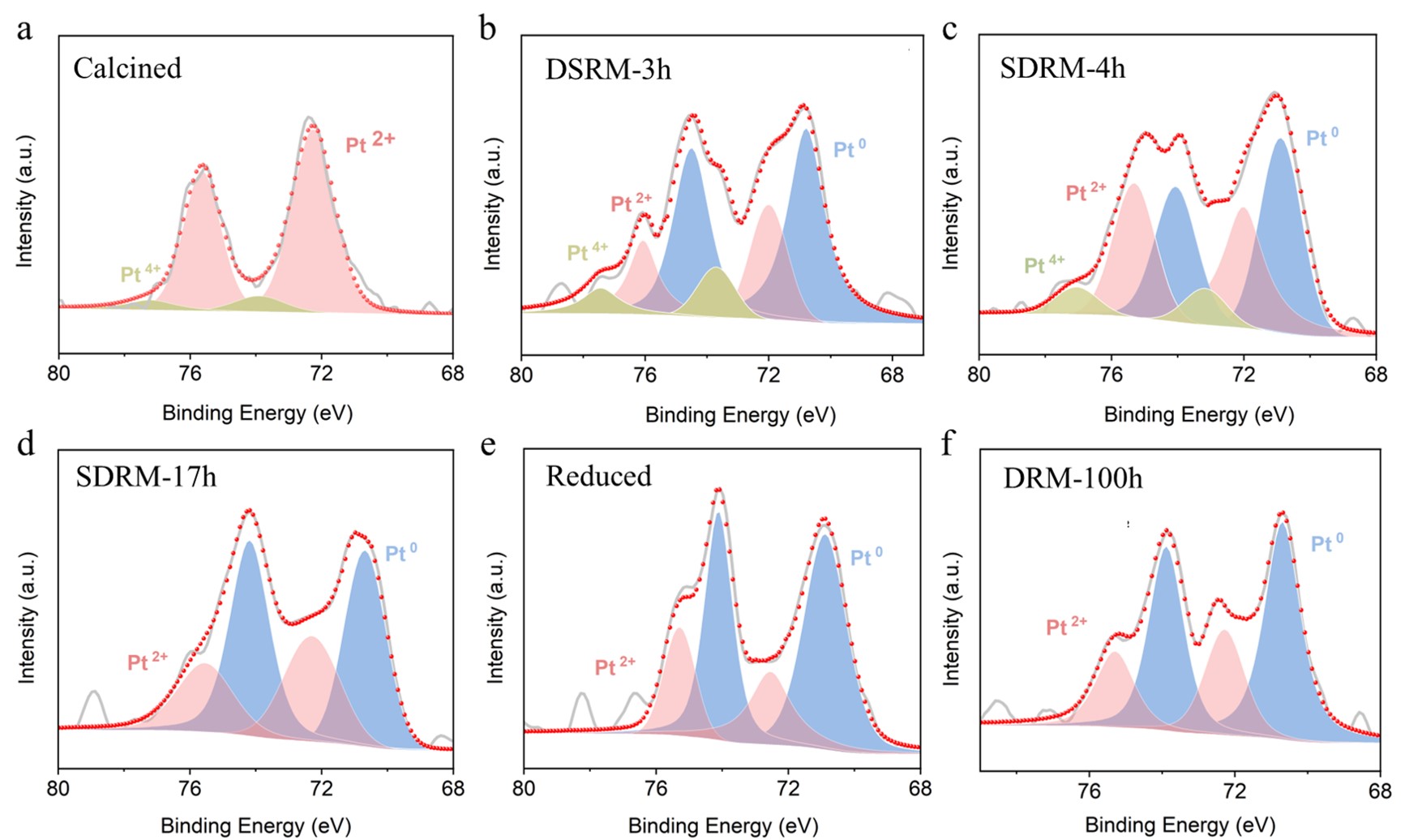


Figure S32. XPS Pt 4f spectra of Pt/CeO_2_ catalyst in (a) Calcined, (b) H_2_S (10 ppm) treatment for 3 h, (c) self-recovery for 4 h, (d) self-recovery for 17 h, (e) Reduced, (f) DRM-100h conditions. The grey lines are the raw data and the red points are the fitted results.

For the Pt 4f spectra, the 71.9 eV (4f 7/2) peak corresponds to the Pt^0^ species, and 72.0 eV (4f 7/2) and 74.0 eV (4f 7/2) correspond to the Pt^2+^ species and the Pt^4+^ species, respectively.^[15, 16]^ It can be seen that even after 800 °C of reduction, there were still Pt^ɑ+^ species present, which is attributed to the SMSI between Pt and CeO_2_.


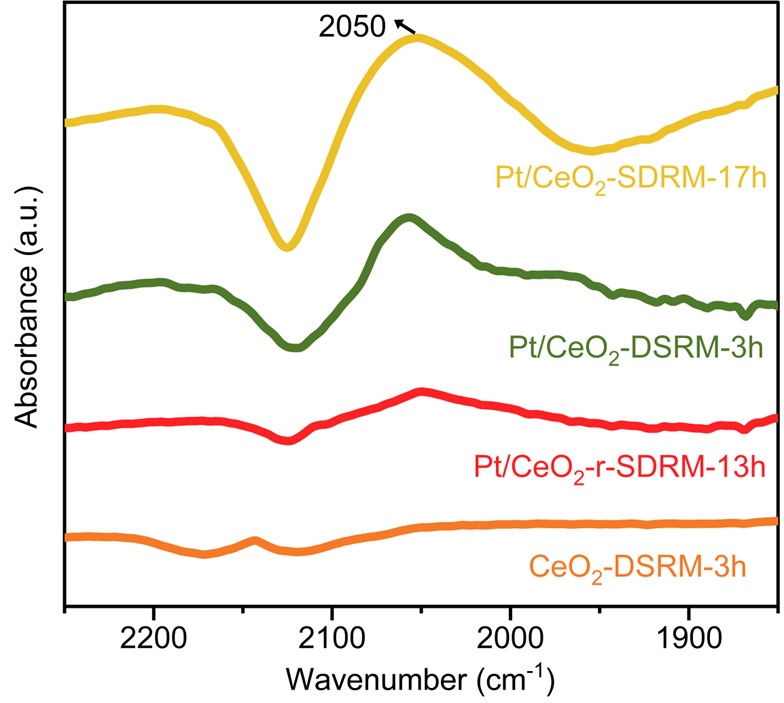


Figure S33. In situ CO-DRIFTS spectra on the surface over various catalysts.


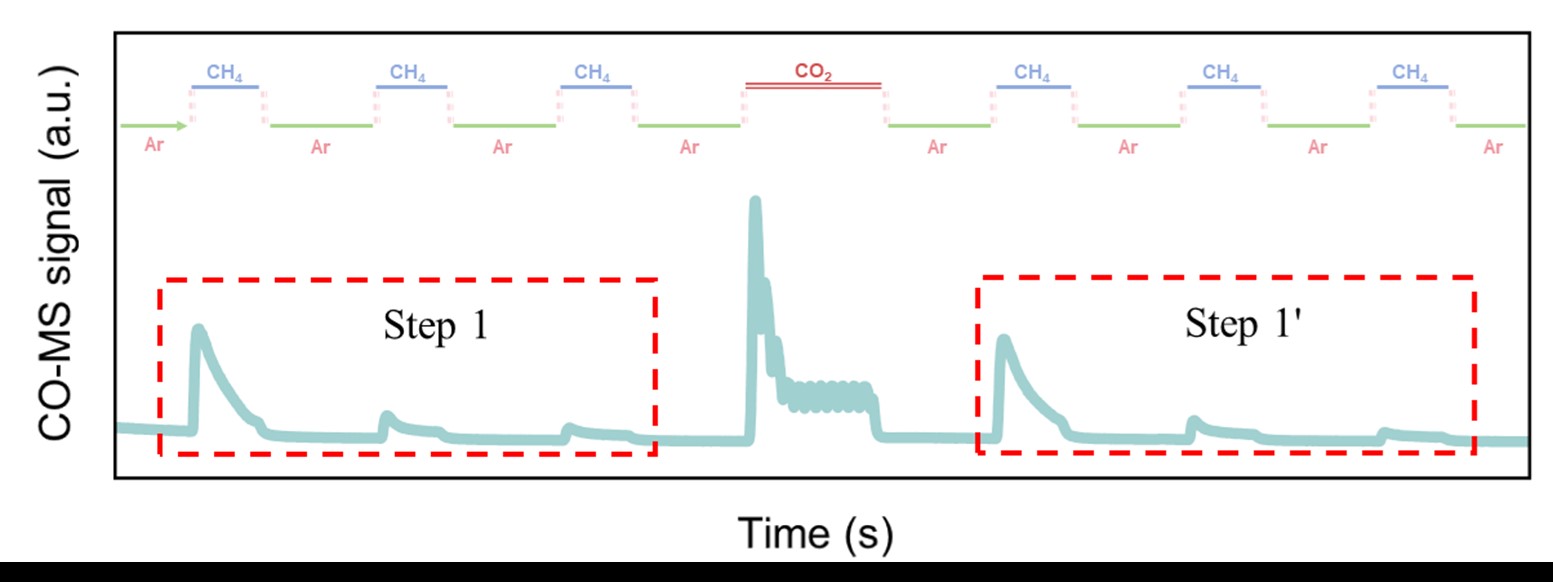


Figure S34. The Mutipulse coupling online MS experiments were conducted at 600 °C of Pt/CeO_2_ catalysts. CH_4_ (mL·min^-1^) was injected for 2 min alternately with the Ar purged for 5 min. Three injections of methane followed by 5 min of CO_2_.


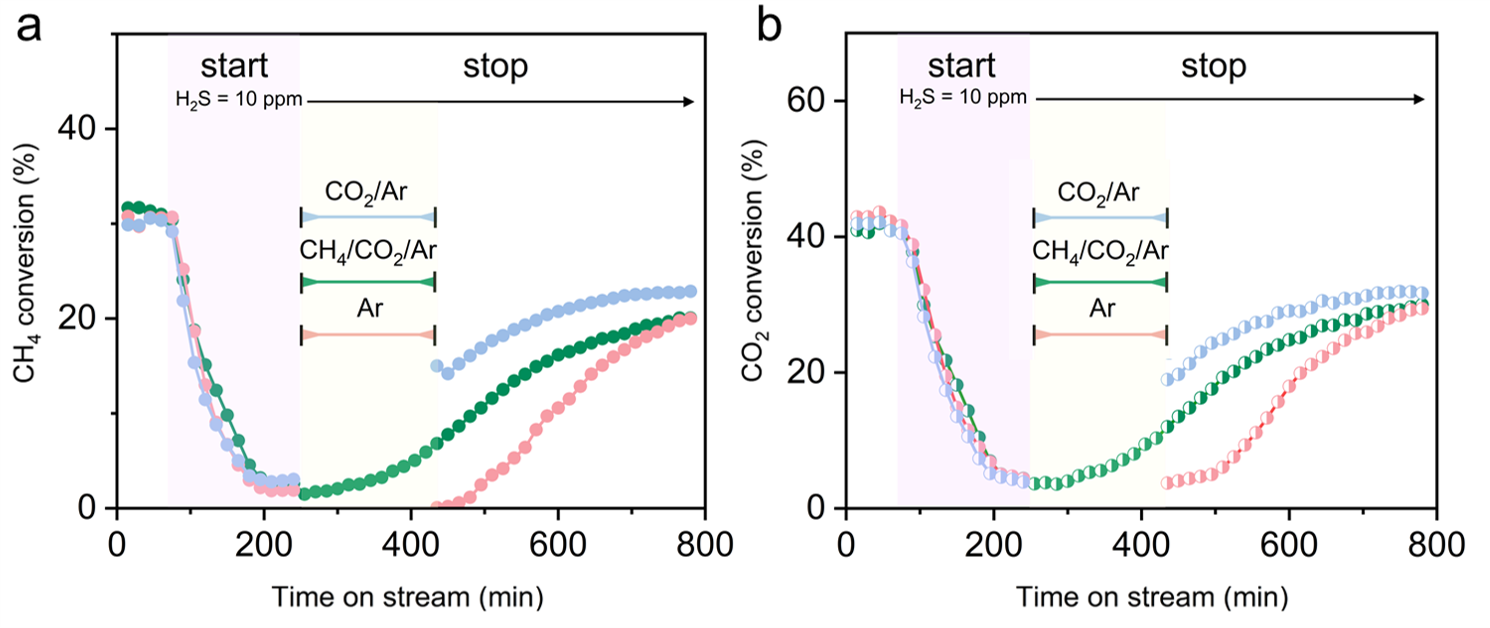


Figure S35. (a) CH_4_ conversion and (b) CO_2_ conversion following a 3 h self-recovery period at different atmospheres. After exposure to 10 ppm H_2_S, CO_2_/Ar and Ar environments were created by stopping the flow of CH_4_ or both CH_4_ and CO_2_, respectively.


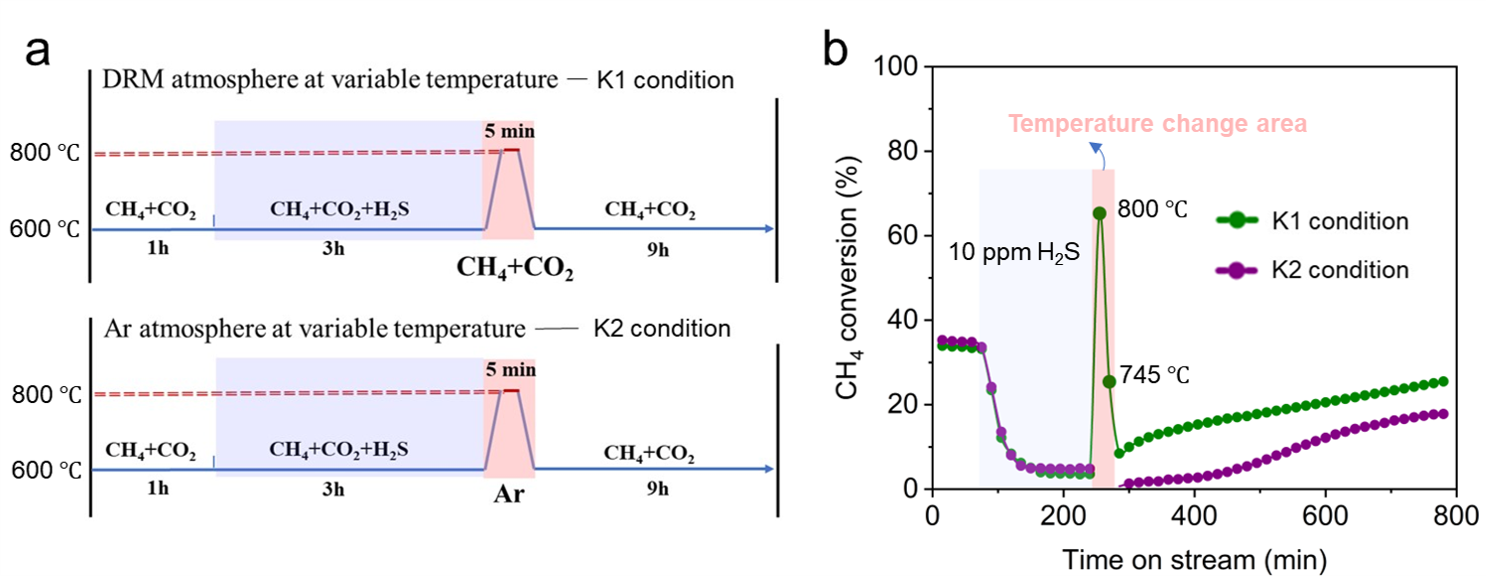


Figure S36. (a) Diagrams of the reaction processes. (b) The CH_4_ conversion image of Pt/CeO_2_ catalyst with variable condition.

Controlled-variable experiment: After a 3 h poisoning experiment, the H_2_S was stopped and the catalyst self-recovery was compared between the DRM atmosphere and the Ar atmosphere by raising the temperature to 800 °C and holding it for 5 min. As shown in the figure above, it was found that the catalyst CH_4_ conversion could not be recovered even when the temperature was raised to 800 °C in an inert atmosphere.


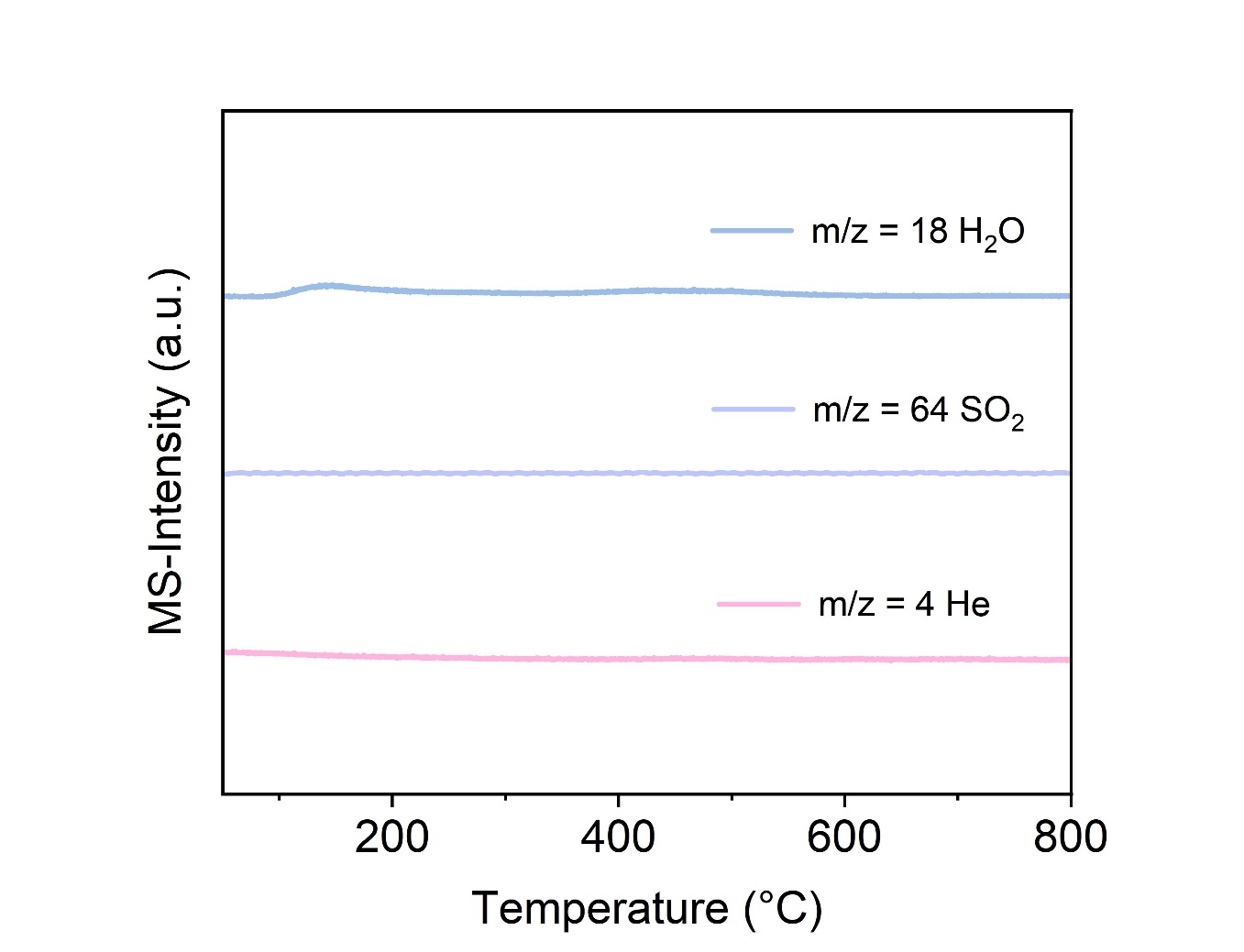


Figure S37. He-TPDC of Pt/CeO_2_ catalyst after H_2_S poisoning 3 h.


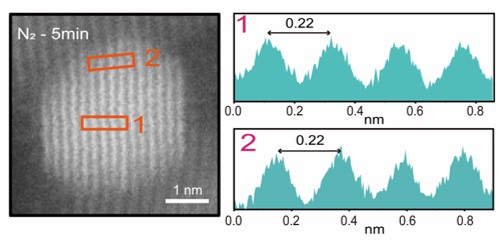


Figure S38. High-resolution STEM image of the Pt/CeO_2_ catalyst following 5 min of N_2_ treatment, along with the line profiles extracted from the region marked by the orange box.


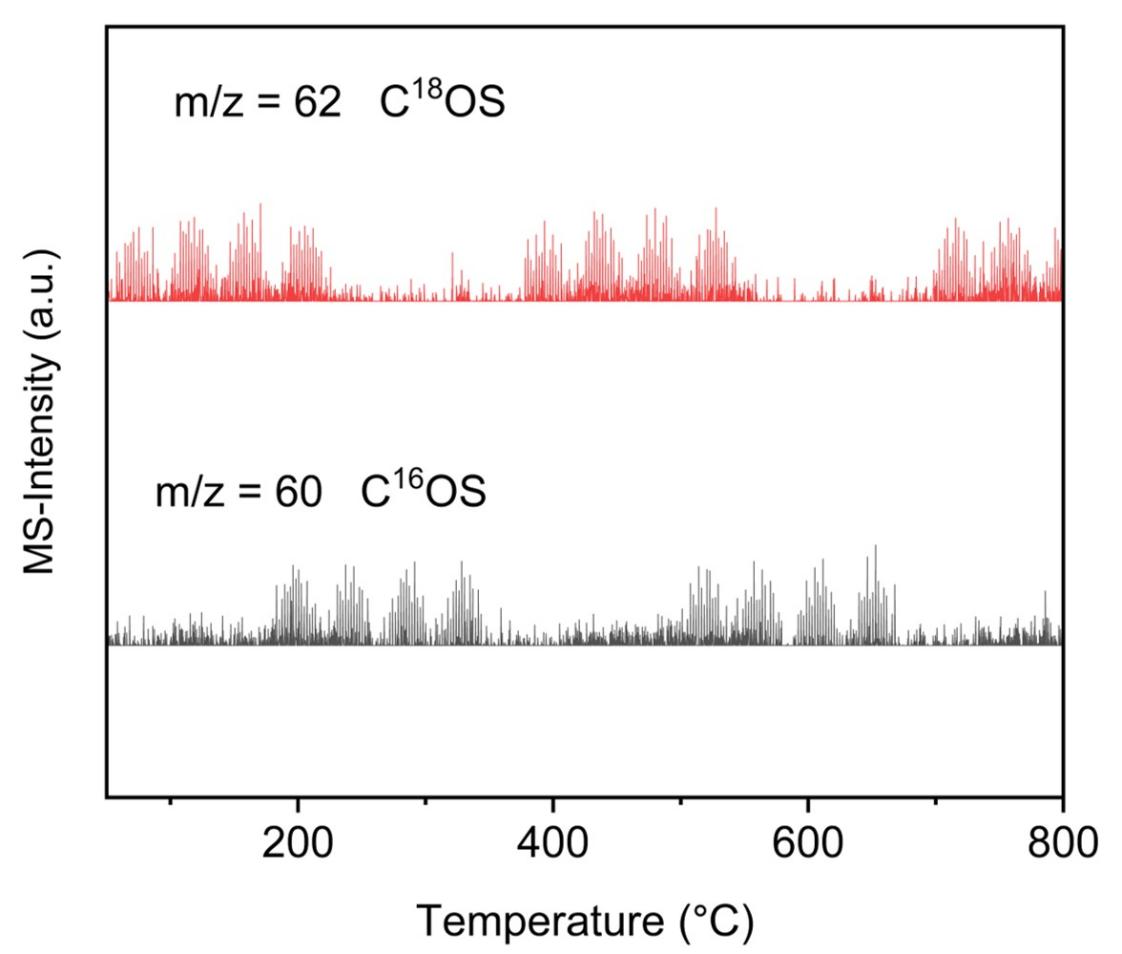


Figure S39. Mass spectrometry images of C^18^OS and C^16^OS signals during C^18^O_2_-TPSR experiments over sulfur-poisoned Pt/CeO_2_ catalyst.


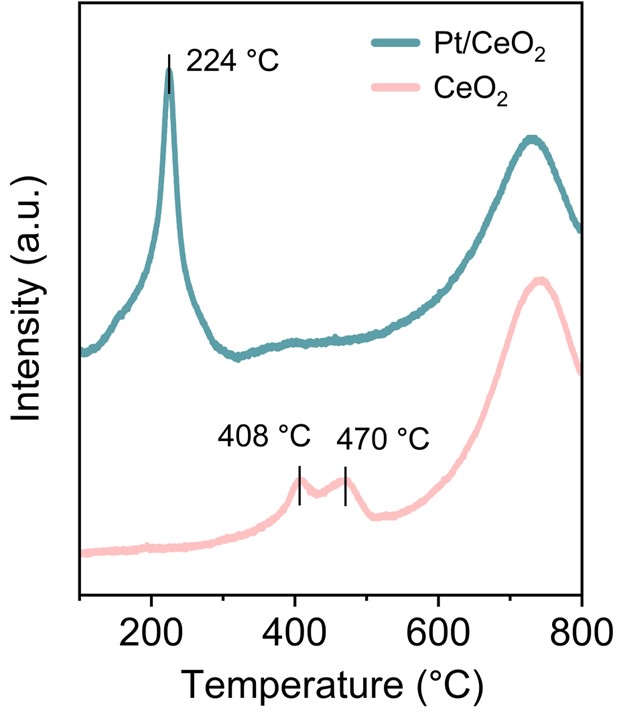


Figure S40. H_2_-TPR profiles of different Pt-loaded catalysts and CeO_2_ catalyst.

It is widely believed that platinum promotes the reduction of cerium oxide surfaces at low temperatures by overflowing hydrogen to neighboring cerium oxide surfaces. We also verified this based on our H_2_-TPR results: as shown above, when Pt is loaded onto cerium oxide, it is consumed in the low-temperature section (224 °C) and the amount of consumption (0.269 mmol/g) is much larger than the amount of hydrogen consumed by the oxygen on the surface of cerium oxide (0.109 mmol/g) and the amount of loading of Pt (0.05 mmol/g). In addition, the decrease in the hydrogen consumption of cerium oxide bulk phase oxygen in the Pt/CeO_2_ catalyst indicates that the addition of Pt mainly affects the surface region of cerium oxide. Moreover, greatly advanced surface oxygen activation temperature in Pt/CeO_2_ suggests that the incorporation of platinum effectively activates the lattice oxygen on the CeO_2_ surface, thereby enhancing its reactivity within a specific range.^[17, 18]^


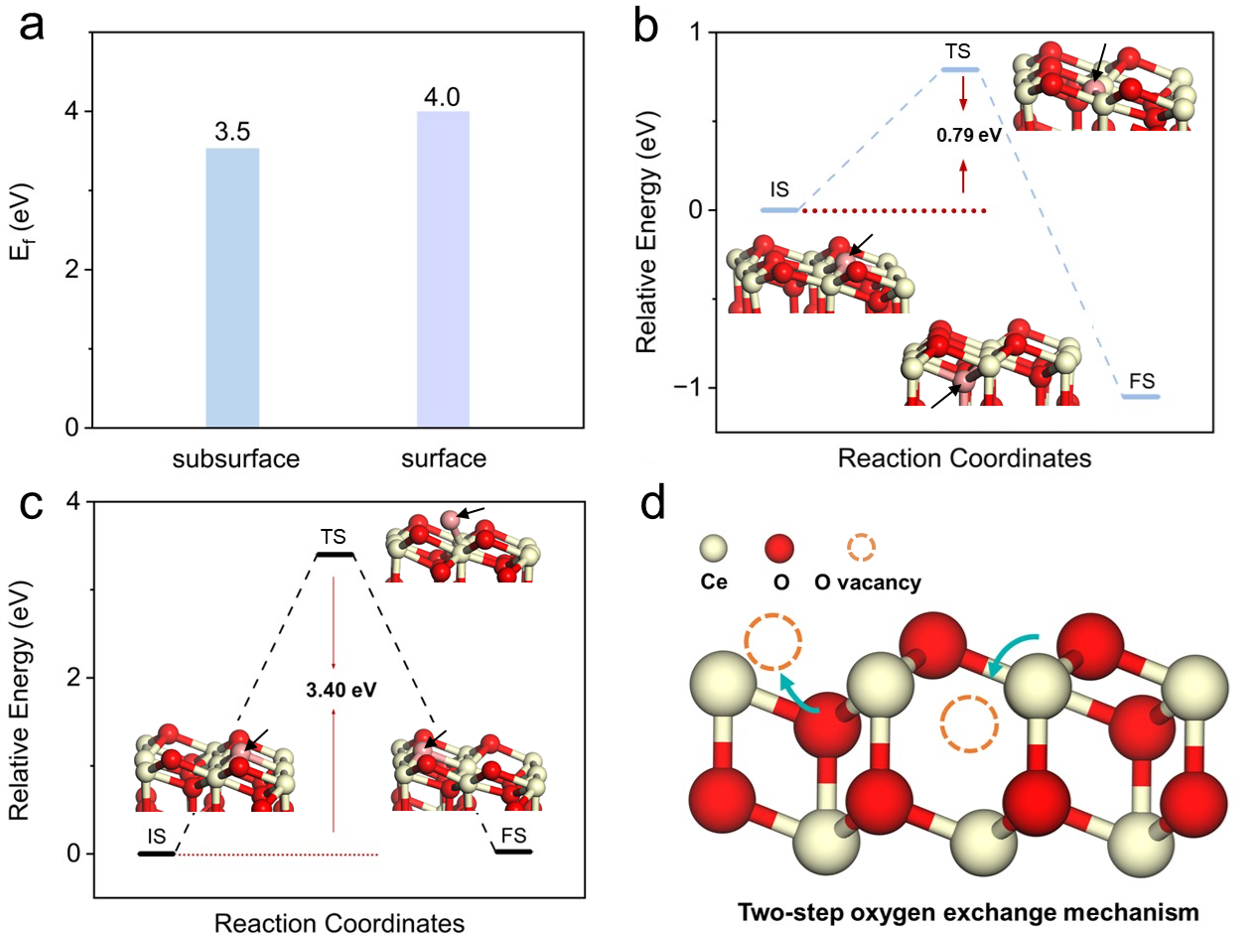


Figure S41. Calculated DFT simulations of clean cerium oxide: (a) Histogram of the vacancy formation energy (E_f_) of surface oxygen and subsurface oxygen. (b) Potential energy image of surface oxygen migration to the subsurface. (c) The potential energy image of surface oxygen migration to neighboring oxygen vacancy. IS: initial state; TS: transition state; FS: final state. (d) Oxygen migration path overview plot. Ce, beige; O, red; migrating oxygen atom, pink (marked by arrow).


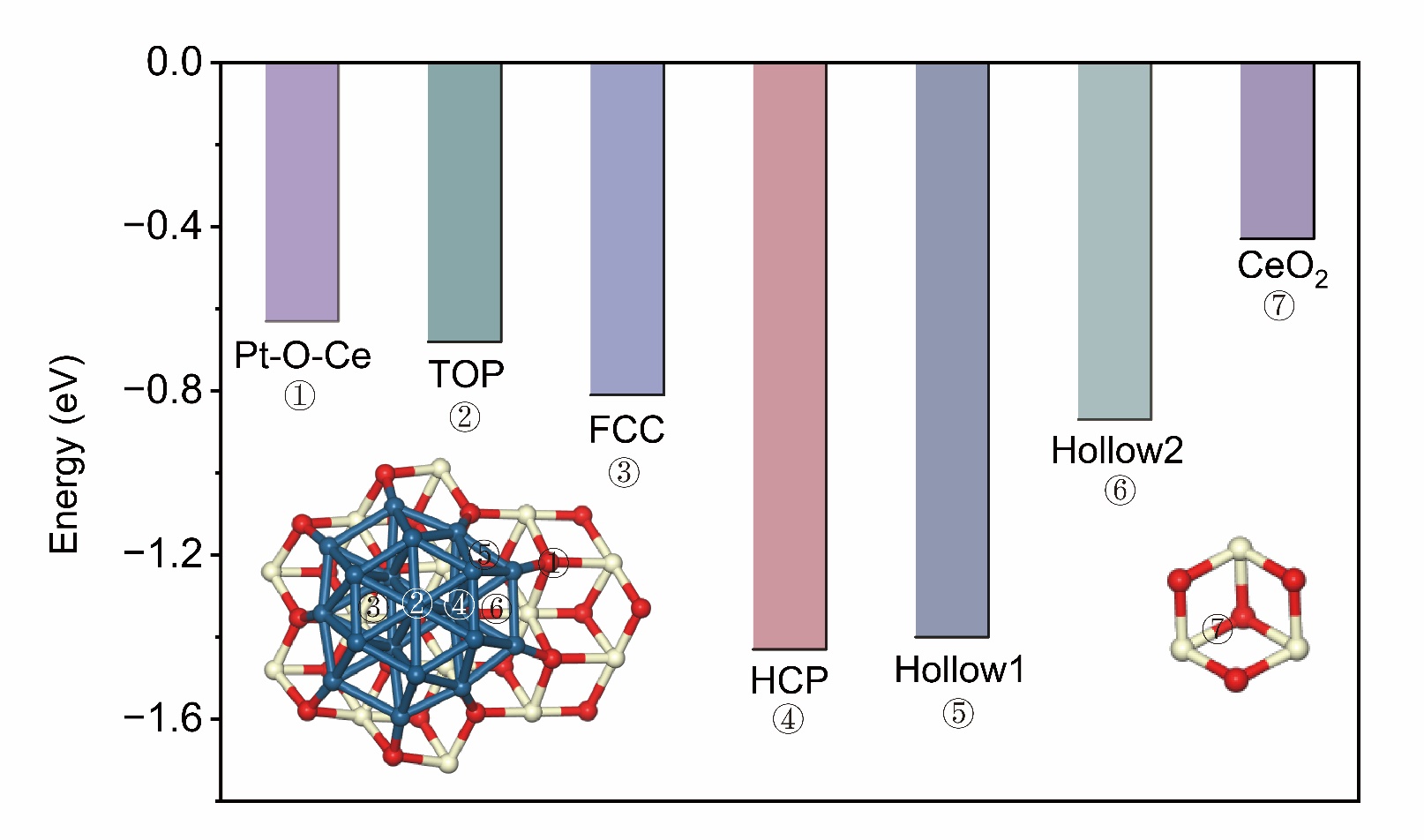


Figure S42. Adsorption Energy of H_2_S on Different Sites on Pt_19_/CeO_2_(111).


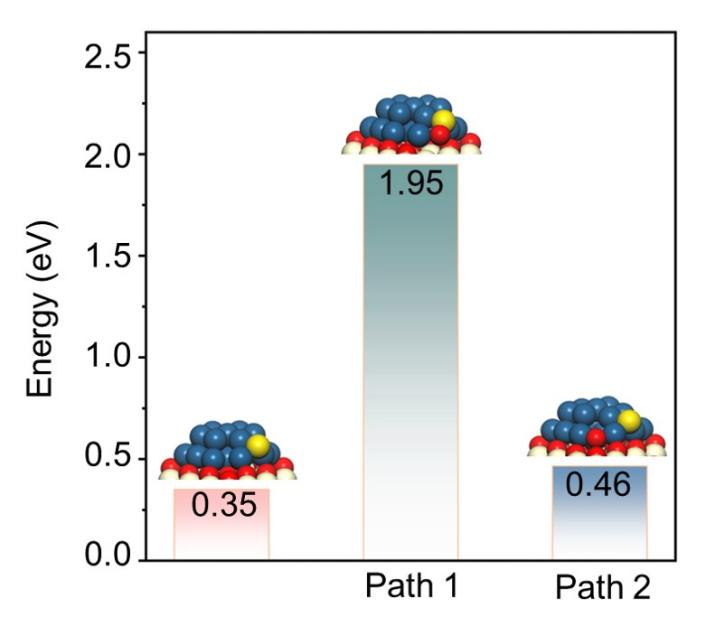


Figure S43. Schematic Energy for Direct Oxygen-Sulfur Bonding (Path 1) and RLOS (Path 2) on Pt_19_/CeO_2_(111). Pt, navy; Ni, purple; O, red; S, yellow.


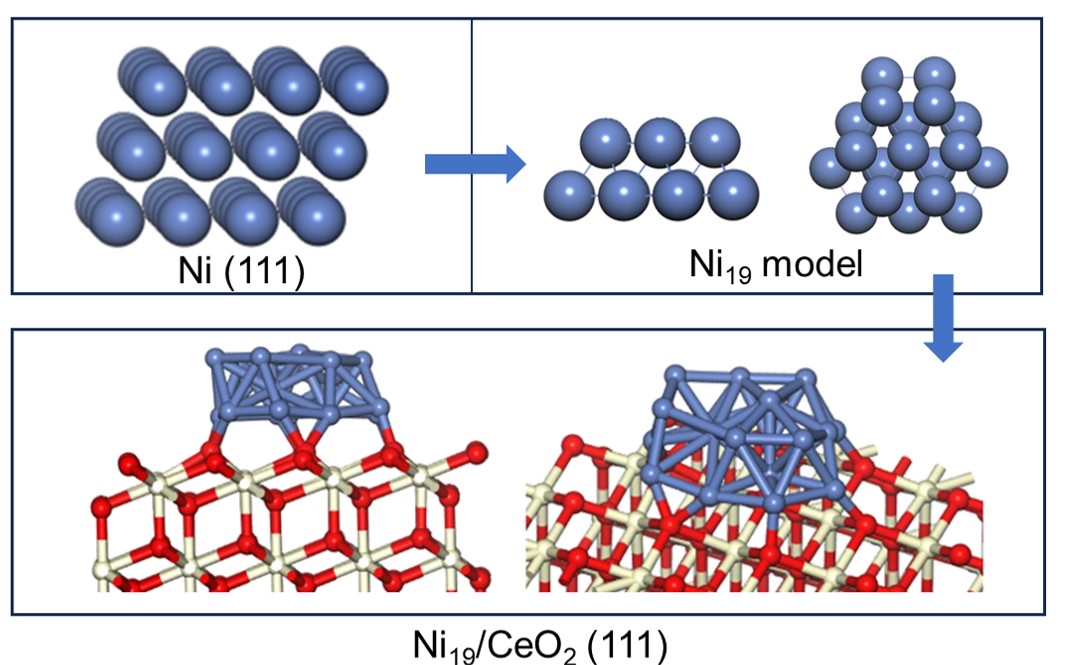


Figure S44. Schematic diagram of Ni_19_/CeO_2_(111). Ni, purple; O, red; S, yellow.


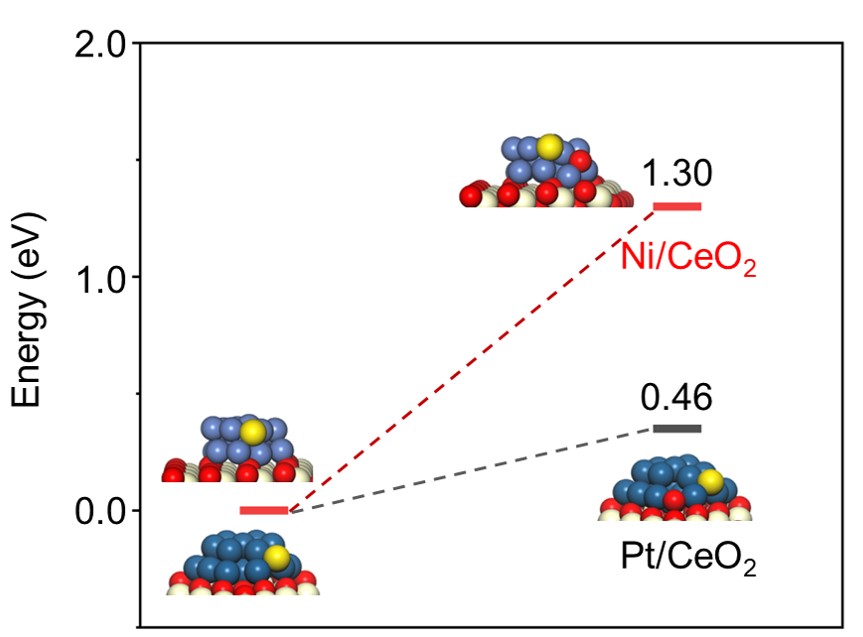


Figure S45. Energy Comparison of RLOS Processes on Pt_19_/CeO_2_(111) and Ni_19_/CeO_2_(111). Pt, navy; Ni, purple; O, red; S, yellow.


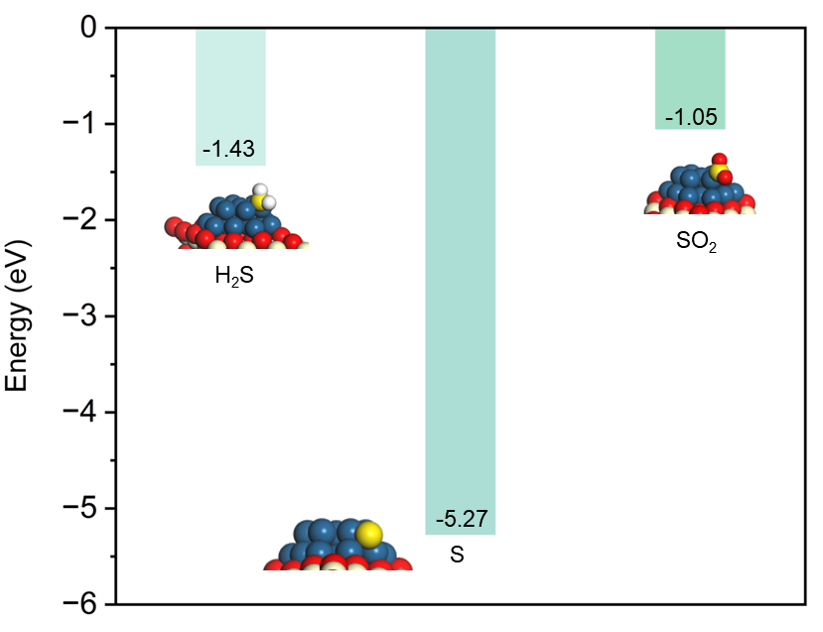


Figure S46. Adsorption energy for H_2_S, S, and SO_2_ on Pt_19_/CeO_2_ (111).


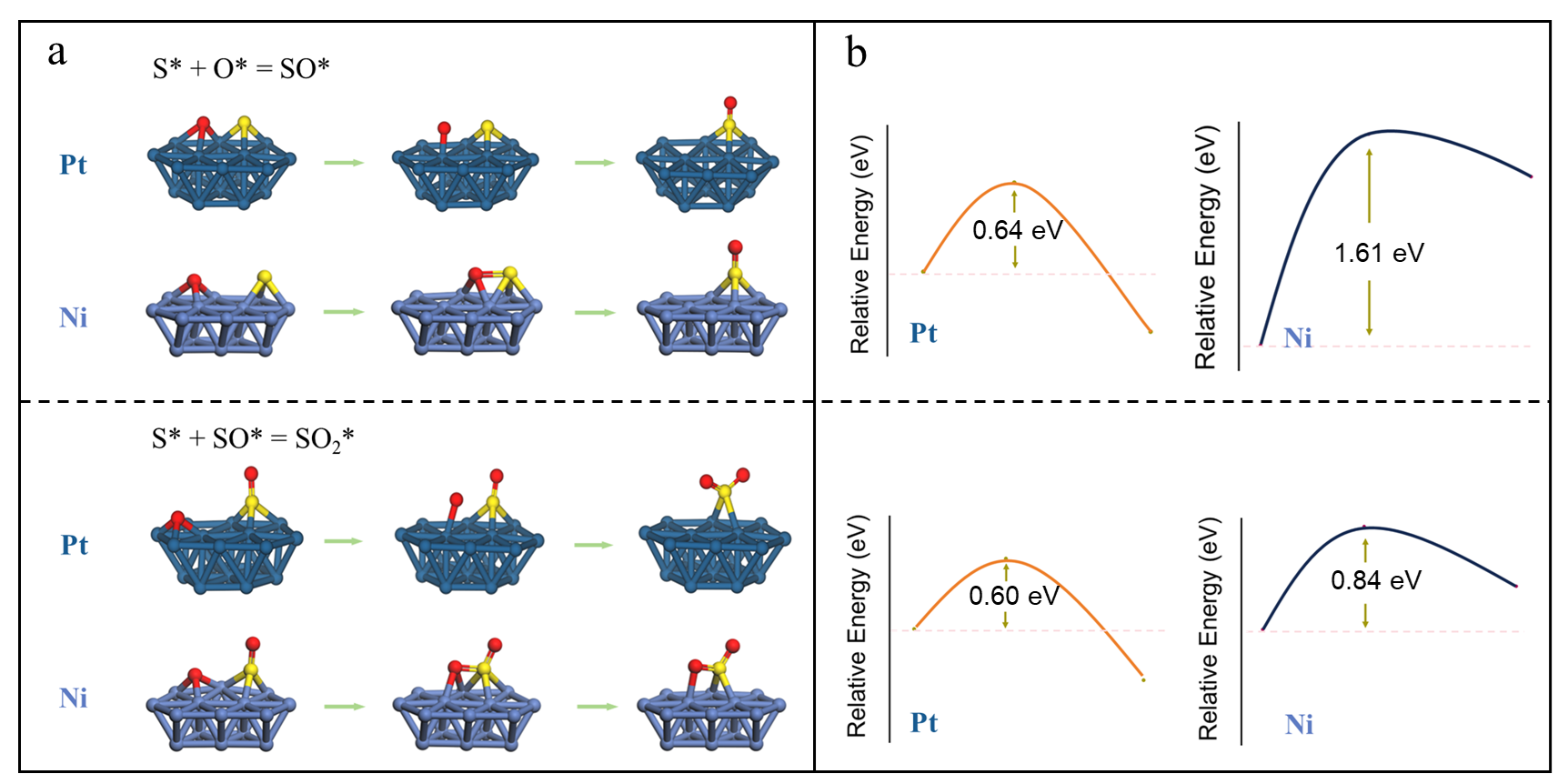


Figure S47. Sulfur-oxygenation to SO_2_ steps on Pt (111) and Ni (111). (a) Profiles of initial state, transition state, and final state, (b) Corresponding potential energy images. Pt, navy; Ni, purple; O, red; S, yellow.


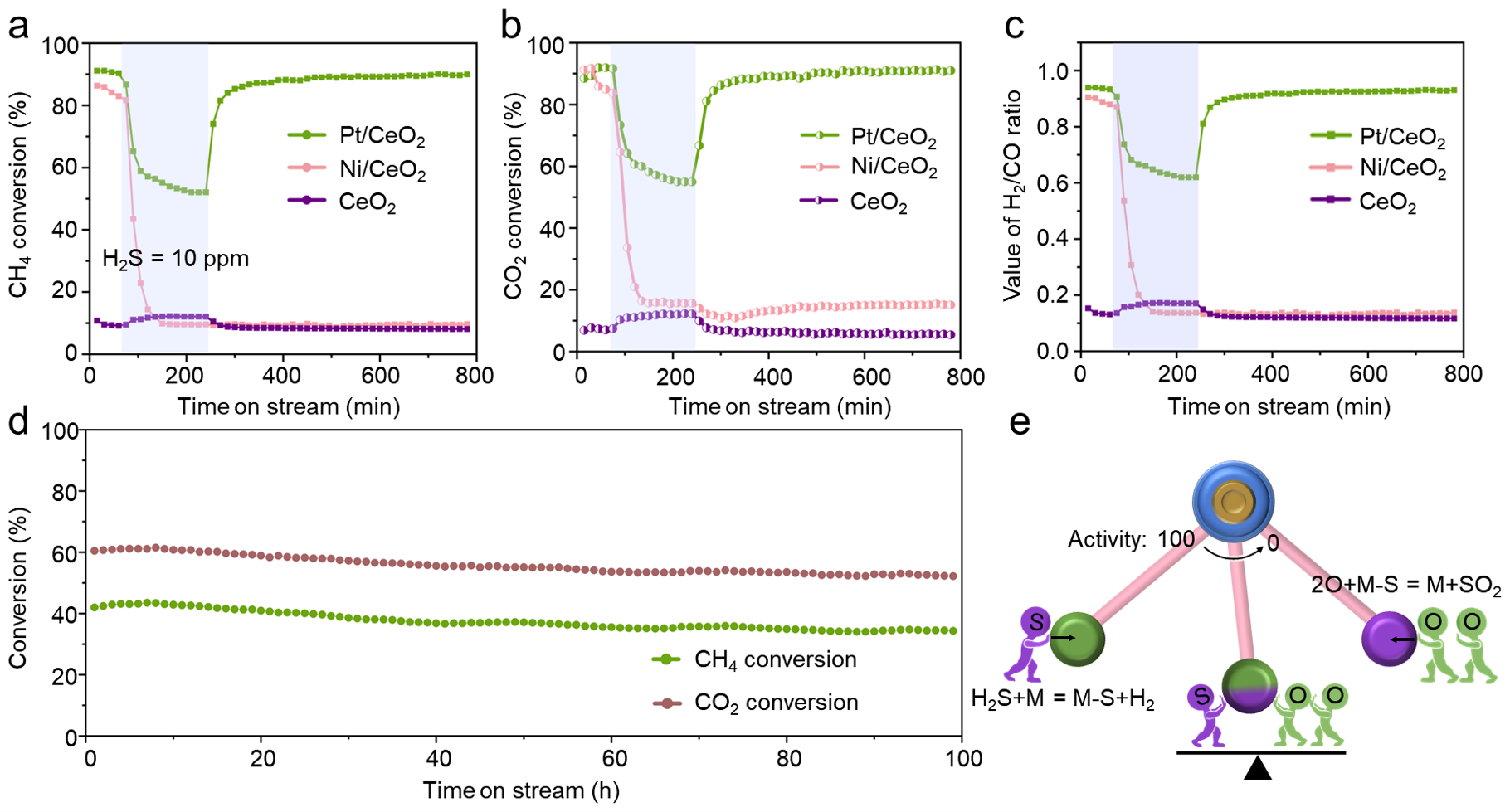


Figure S48. (a) CH_4_, (b) CO_2_ conversion, (c) H_2_/CO ratio of Pt/CeO_2_, Ni/CeO_2_, and CeO_2_ at 800 °C with or without H_2_S. (d) Stability of Pt/CeO_2_ catalysts in the presence of H_2_S for 100 h. (e) Schematic representation of the equilibrium state of Pt/CeO_2_ catalysts. The DRM reaction was carried out at 800 °C with WHSV of 50,000 mL·g_cat_^–1^·h^–1^. Control of H_2_S concentration at 10 ppm in case of poisoning.

Building on the previous discussion, we have elucidated the underlying reasons for the self-recovery observed in Pt/CeO_2_ catalysts. Modulating the reaction atmosphere and temperature, without altering the catalyst’s composition, proved effective in facilitating the RLOS-driven regeneration process. The Pt/CeO_2_ catalyst shows incomplete recovery from H_2_S poisoning at 600 °C, with removal of residual S occurring at higher temperatures. The temperature was elevated to the optimal range of 700-800 °C for the DRM reaction with the aim of enhancing the efficiency of the sulfur removal process. This strategic adjustment was based on the understanding of the interplay between temperature, lattice oxygen mobility, and the rate of RLOS in CeO_2_. Thus, higher temperature promotes faster lattice oxygen exchange between CeO_2_ and Pt, enabling more efficient oxidation and removal of surface sulfur species. It is anticipated that increasing the temperature will significantly influence the movement of lattice oxygen and the reverse spillover process through thermally activated diffusion. We aimed to accelerate the reaction rate and reflect the equilibrium relationship of the Pt/CeO_2_ catalyst reaction in the presence of H_2_S. The findings were illustrated in Figure S48, where the DRM process exhibited a significant heat absorption reaction. Upon reaching a temperature of 800 °C, the Pt/CeO_2_ catalyst demonstrated an enhanced conversion rate of CH_4_ to 87% and CO_2_ to 92%. These near-equilibrium conversions underline the strong catalytic performance even under sulfur-containing conditions. Furthermore, the catalytic activity remained stable for 100 h during the reaction, as depicted in Figure S49. The coke-free at 800 °C was also verified by in-situ Raman. Upon treatment with 10 ppm H_2_S, it was observed that the activity of the Ni/CeO_2_ catalyst continued to decline rapidly, with no signs of self-recovery in the active sites. However, the Pt/CeO_2_ catalyst exhibited a remarkable resistance to rapid deactivation upon encountering H_2_S. Its activity can be restored to 90% of its original level within 1 h after stopping H_2_S. This long-term durability highlights that sulfur oxidation via RLOS continues efficiently over extended operation. Notably, even after being exposed to H_2_S poisoning for a duration of 100 h, the catalyst still maintained a significant level of CH_4_ and CO_2_ conversion, with 34% and 52% respectively. This indicated that the system was in equilibrium after balancing the relationship between sulfur adsorption and sulfur desorption, and a relatively stable performance ensued.


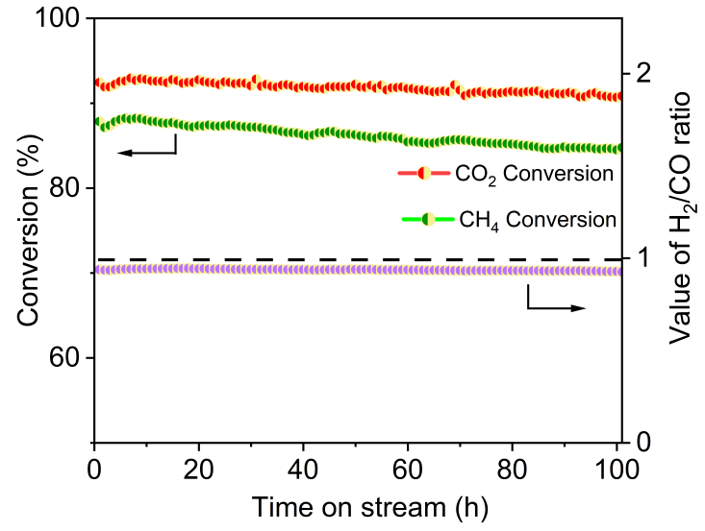


Figure S49. Conversion and H_2_/CO ratio of Pt/CeO_2_ with time on stream of 100 h at 800 °C.


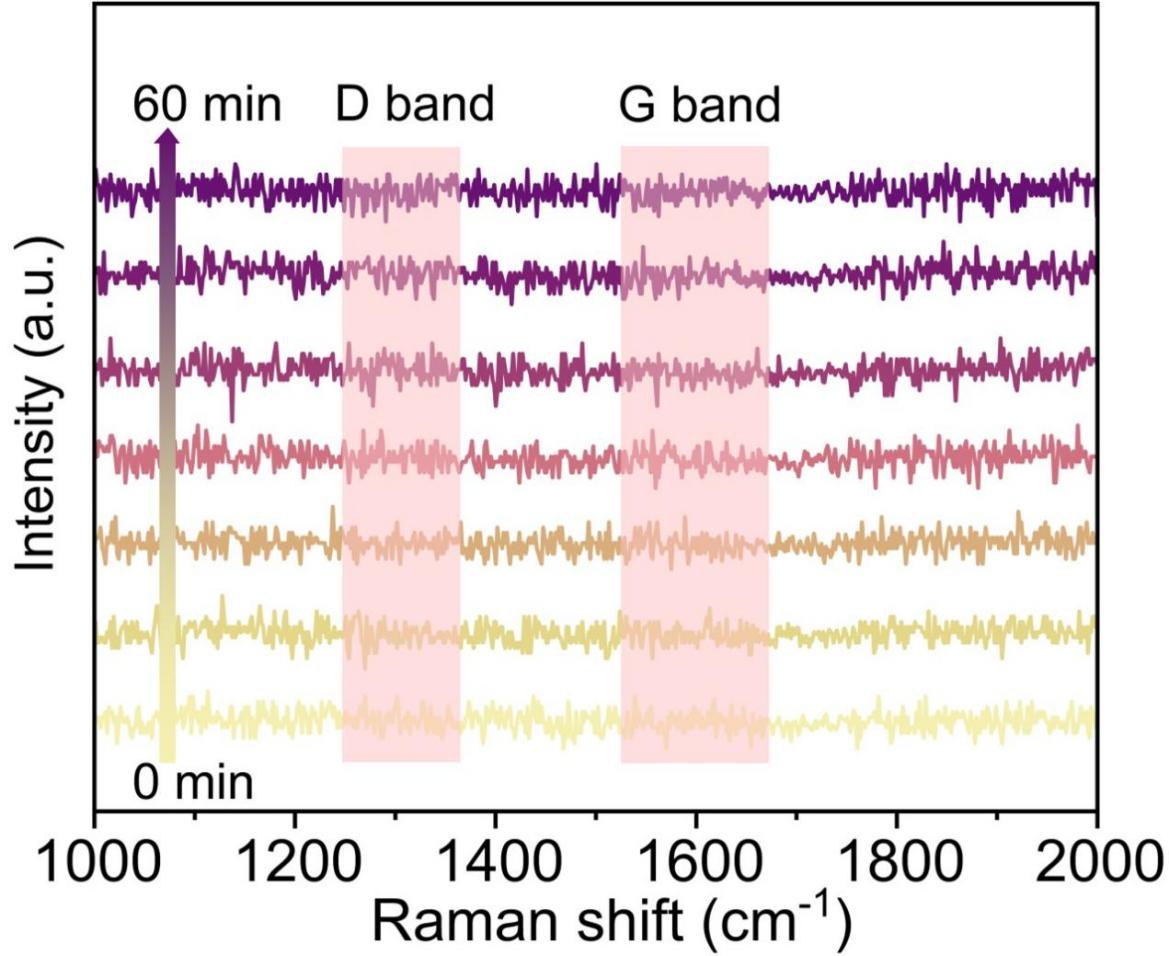


Figure S50. In situ Raman spectra of the Pt/CeO_2_ catalyst in a flow of CH_4_/CO_2_ (15/15 mL/min) at 800 °C.


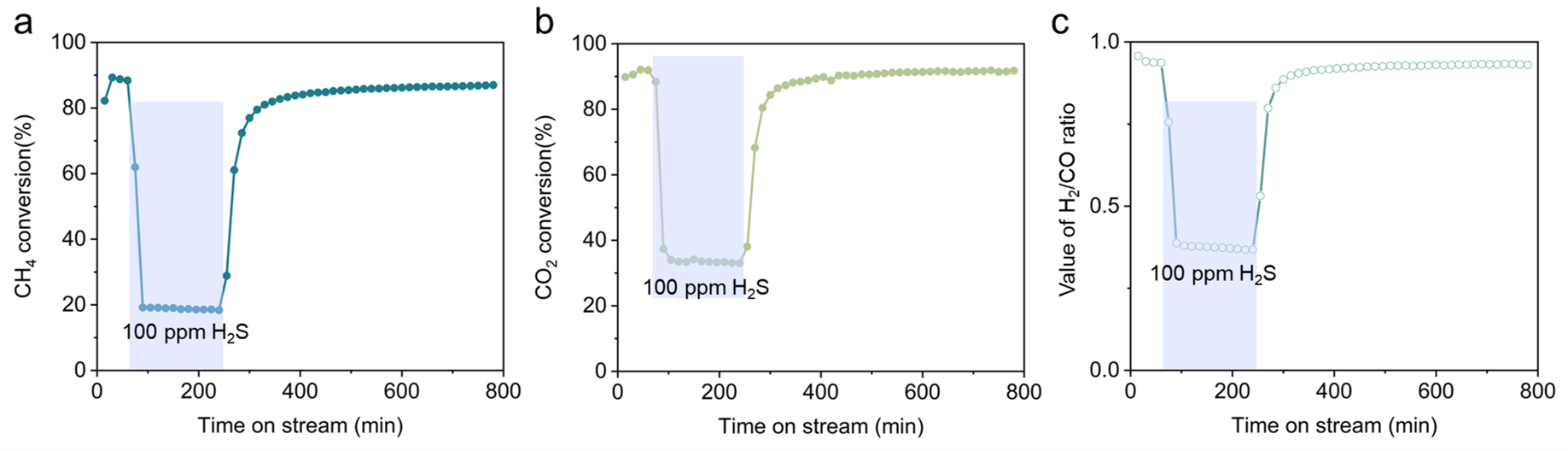


Figure S51. Profiles of (a) CH_4_ conversion, (b) CO_2_ conversion, and (c) H_2_/CO ratio for Pt/CeO_2_ catalyst self-recovery after suffering 3 h poisoning with H_2_S of 100 ppm. The reaction was carried out at 800 °C with WHSV of 50,000 mL·g_cat_^-1^·h^-1^.

Notably, when the concentration of H_2_S was increased to 100 ppm, the Pt/CeO_2_ catalyst continued to exhibit an equilibrium step, as depicted in the Figure S51. Note that, the Pt/CeO_2_ catalyst displayed a prompt recovery, with its activity rebounding to 93% of the initial level within a single hour after the H_2_S shutdown, regardless of whether it was exposed to 10 ppm or 100 ppm of H_2_S.

**Table S1.** EXAFS fitting parameters at the Pt L_3_-edge for Pt foil and calcined Pt/CeO_2_.

|  | | | | | |
| --- | --- | --- | --- | --- | --- |
| **sample** | **Scattering** | **R (Å)^a^** | **CN^b^** | **σ^2^ (10^-3^)^c^** | **R-factor^d^** |
| Pt foil | Pt-Pt | 2.79(0.02) | 12 | 3.2(0.4) | 0.006 |
| Calcined Pt/CeO_2_ | Pt-O | 1.98(0.02) | 6.2(0.9) | 3.7(0.6) | 0.038 |
|  | Pt-O-Ce | 3.68(0.02) | 7.3(0.9) | 8.7(0.7) |  |
| PtO_2_ | Pt-O | 2.01(0.02) | 8.5(0.7) | 6.7(0.5) | 0.014 |

^a^R is the interatomic distance (the bond length between central atoms and surrounding coordination atoms); ^b^CN is the coordination number; ^c^σ^2^ is Debye-Waller factor (a measure of thermal and static disorder in absorber-scatter distances); ^d^R factor is used to value the quality of the fitting.

**Table S2.** Corresponding active metal content for different catalysts as obtained by ICP analysis.


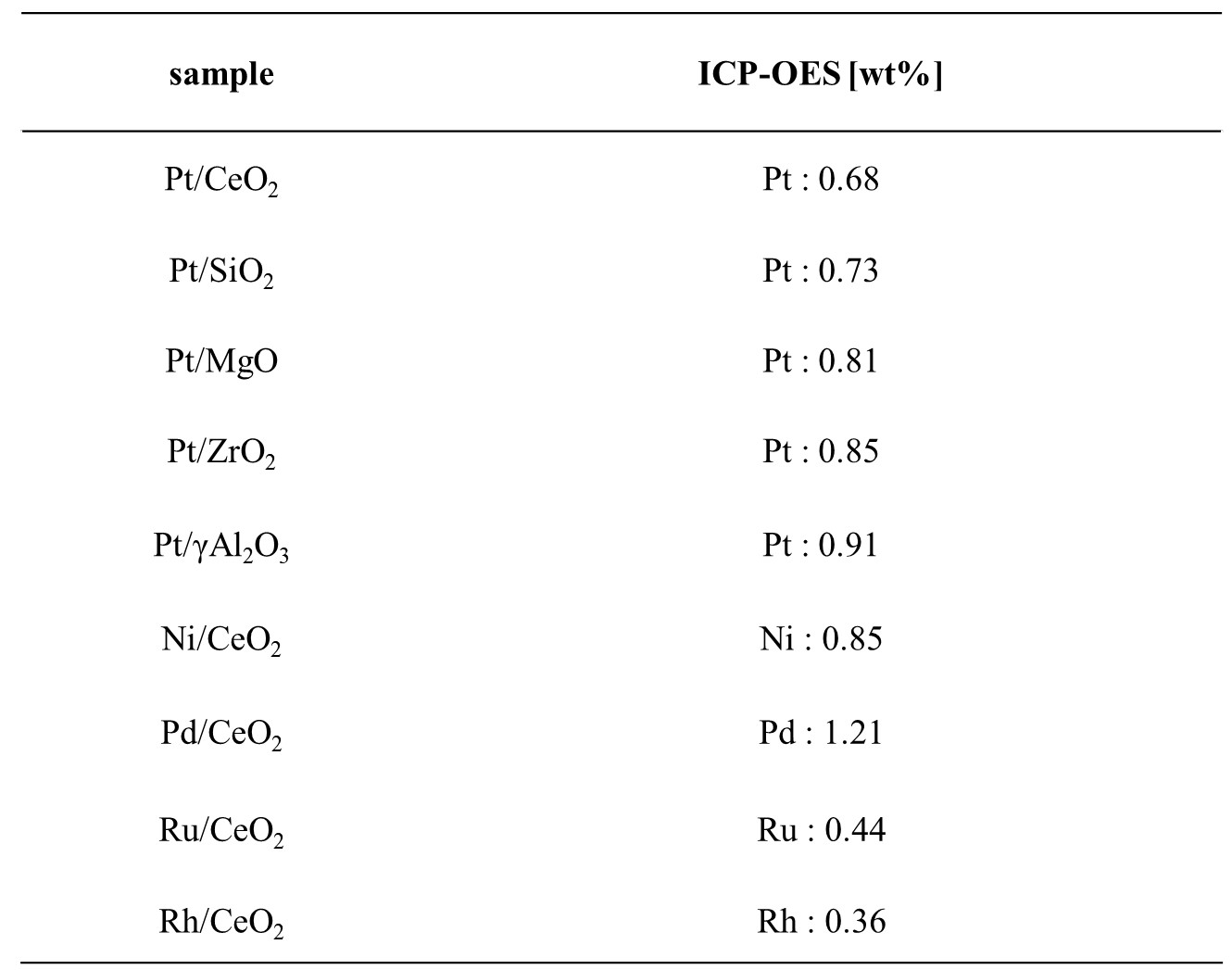


**Table S3.** Carbon and sulfur content in Pt/CeO_2_ catalyst as obtained by CS 600CR.


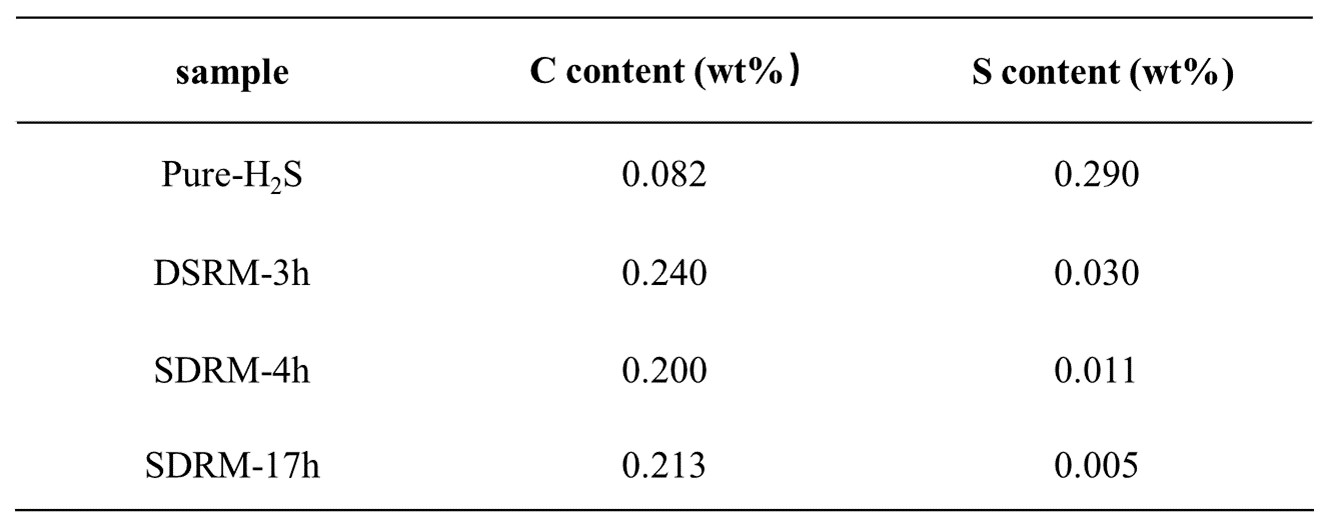


**Table S4.** Carbon and sulfur content in other catalysts as obtained by CS 600CR.


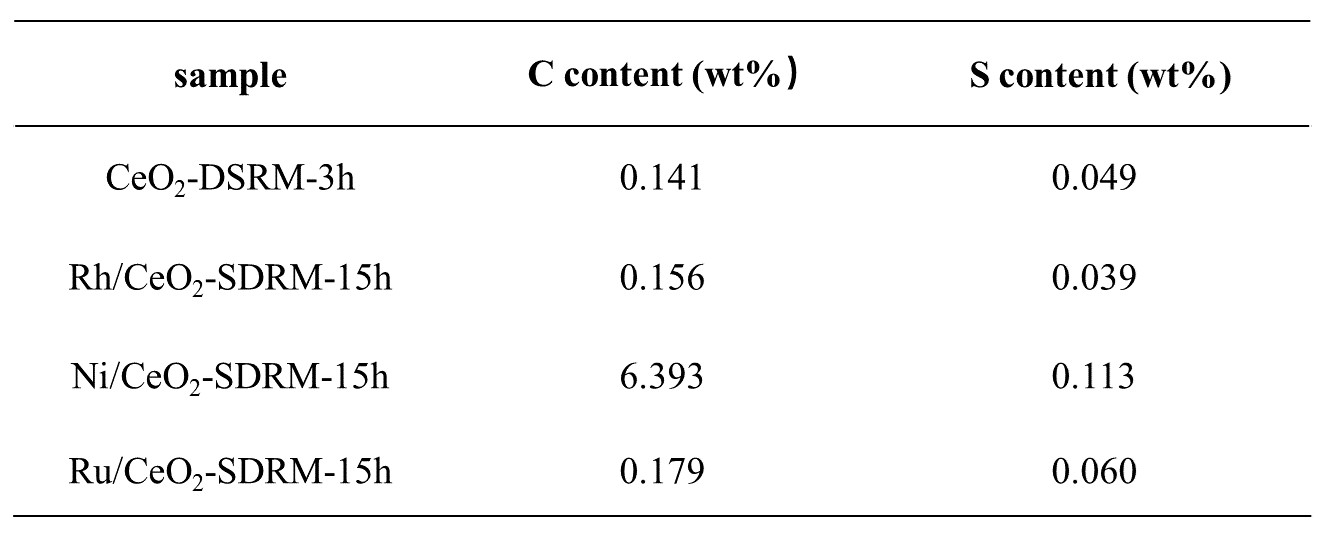


**Table S5.** Composition of surface Pt species calculated from Pt 4f XPS data for as synthesized, reduced, and spent Pt/CeO_2_.


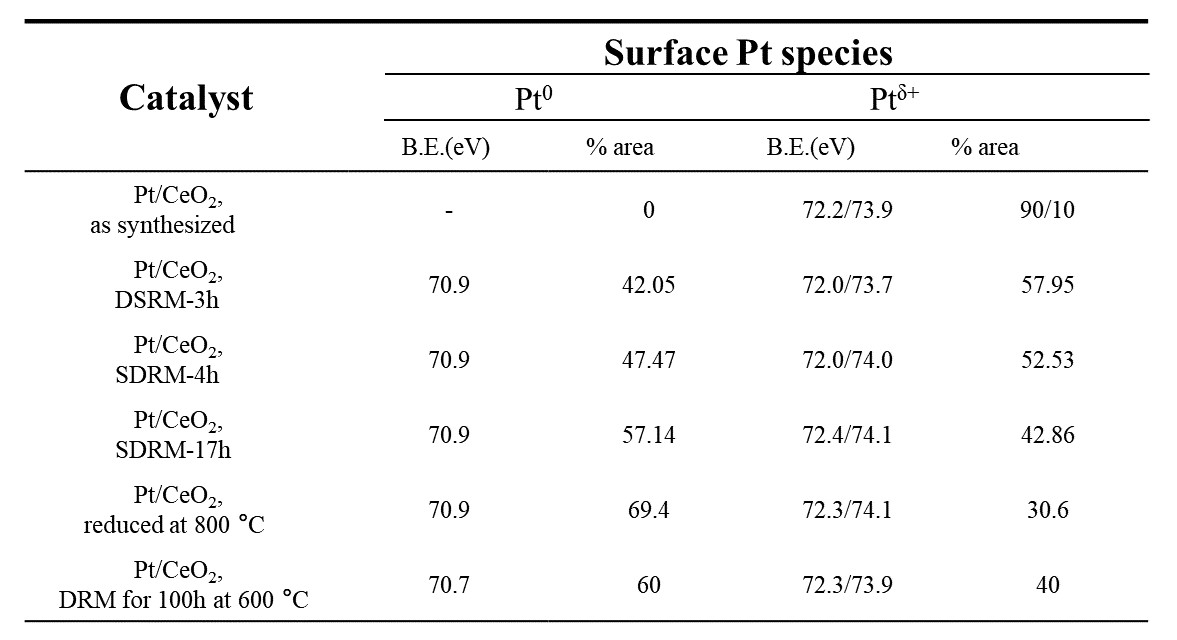


| **Table S6.** Phase compositions obtained from LCF analysis of normalized Pt L_3_-edge XANES spectra for Pt/CeO_2_ sample. | | | | | |
| --- | --- | --- | --- | --- | --- |
| Treatment ^a^ | Phase | Weight Fraction  (%) | Uncertainty ^b^ (%) | R-factor ^c^ | Reduced χ^2 d^ |
| Calcined | Pt | 0.326 | 0.015 | 0.00357 | 0.0424 |
|  | PtO_2_ | 0.674 | 0.015 |  |  |
| Reduced | Pt | 0.970 | 0.01 | 0.00225 | 0.0188 |
|  | PtO_2_ | 0.030 | 0.01 |  |  |
| DSRM-3h | Pt | 0.808 | 0.014 | 0.00421 | 0.0362 |
|  | PtO_2_ | 0.192 | 0.014 |  |  |
| SDRM-4h | Pt | 0.799 | 0.014 | 0.0040 | 0.036 |
|  | PtO_2_ | 0.201 | 0.014 |  |  |
| SDRM-17h | Pt | 0.806 | 0.016 | 0.00554 | 0.0486 |
|  | PtO_2_ | 0.194 | 0.016 |  |  |

^a^ The calcined Pt/CeO_2_ catalyst (Calcined) was treated via two different routes: one involved reduction at 800 °C (Reduced); the other consisted of initial exposure to 10 ppm H_2_S for 3 h (DSRM–3h), followed by regeneration under DRM atmosphere for 4 h (SDRM–4h) and 17 h (SDRM–17h), respectively. ^b^Uncertainty indicates the fitting error, which was used for error bar presentation. ^c^The R-factor reflects the residual difference between experimental and fitted spectra. ^d^Reduced χ² is a goodness-of-fit parameter normalized by degrees of freedom, evaluating the overall reliability of the LCF model.

**Table S7.** Quantitative elemental analysis by EDS for sulfur-poisoned Pt/CeO_2_ catalyst during in situ STEM characterization.


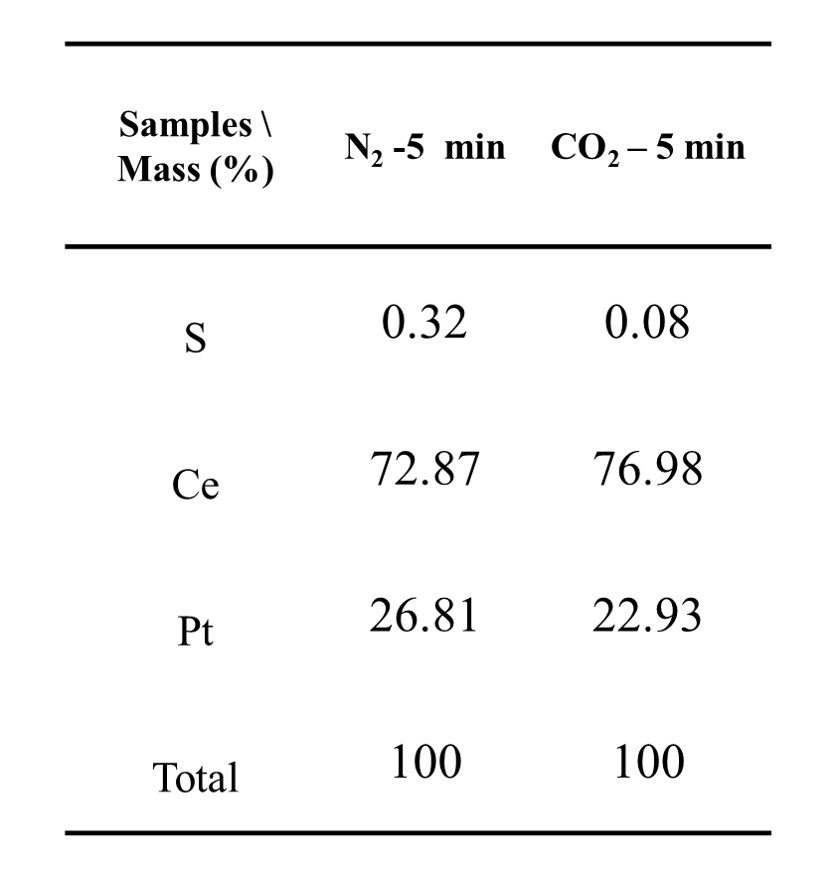


**Table S8.** Composition of catalytic performance for DRM reaction in the presence of H_2_S. (The sample regeneration methods are all break-stop H_2_S)


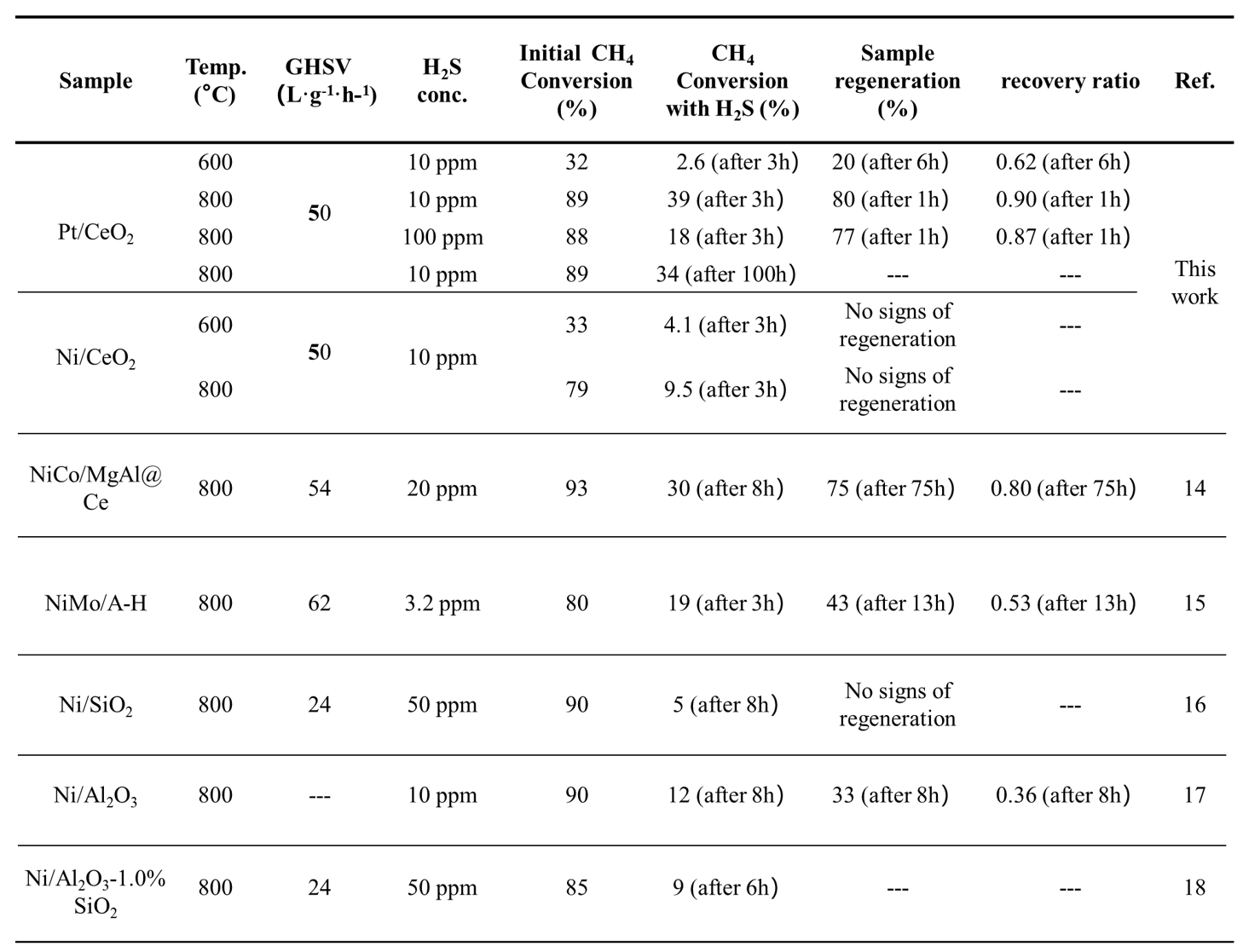


TEM Movie-S1: In-situ TEM of poisoned Pt/CeO_2_ catalyst from 50 °C to 400 °C with a ramp rate of 10 °C min^-1^ in N_2_ gas.

TEM Movie-S2: In situ TEM of a poisoned Pt/CeO_2_ catalyst switched to CO_2_ atmosphere after N2 treatment.

**References**

[1] J. Deng, J. Siewe, C. Sun, J. Yan, “Elucidating the promotional role of Ag in the hydrodechlorination of CHCl_3_ over PtAg/Al_2_O_3_ catalysts” *European Synchrotron Radiation Facility* (2027): doi.org/10.15151/ESRF-ES-1520006712.

[2] B. Ravel, M. Newville, “ATHENA, ARTEMIS, HEPHAESTUS: data analysis for X-ray absorption spectroscopy using IFEFFIT” *J Synchrotron Radiat* **12**, (2005): 537-541.

[3] Kresse, Furthmuller, “Efficient iterative schemes for ab initio total-energy calculations using a plane-wave basis set” *Physical Review B* **54**, (1996): 11169-11186.

[4] G. Kresse, J. Furthmüller, “Efficiency of ab-initio total energy calculations for metals and semiconductors using a plane-wave basis set” *Computational Materials Science* **6**, (1996): 15-50.

[5] n. Blöchl, “Projector augmented-wave method” *Physical Review B* **50**, (1994): 17953-17979.

[6] n. Perdew, n. Burke, n. Ernzerhof, “Generalized Gradient Approximation Made Simple” *Physical Review Letter* **77**, (1996): 3865-3868.

[7] S. Grimme, S. Ehrlich, L. Goerigk, “Effect of the damping function in dispersion corrected density functional theory” *Journal of Computational Chemistry* **32**, (2011): 1456-1465.

[8] A. Heyden, A. T. Bell, F. J. Keil, “Efficient methods for finding transition states in chemical reactions: comparison of improved dimer method and partitioned rational function optimization method” *The Journal Chemical Physics* **123**, (2005): 224101.

[9] K. Momma, F. Izumi, “VESTA 3for three-dimensional visualization of crystal, volumetric and morphology data” *Journal Applied Crystallography* **44**, (2011): 1272-1276.

[10] B. R. Cooper, S. P. Lim, I. Avgin, Q. G. sheng, D. L. Price, “Trade-off between increased size and increased broadening of magneto-optic effects in cerium and uranium systems” *The Journal of Physics and Chemistry of Solids* **11**, (1995): 1509-1516.

[11] H. Harima, “LDA+U method applied for f-electron systems” *Journal of Magnetism and Magnetic Materials.* **226-230**, (2001): 83-84.

[12] X. Zhang, J. Deng, T. Lan, Y. Shen, Q. Zhong, W. Ren, D. Zhang, “Promoting Methane Dry Reforming over Ni Catalysts via Modulating Surface Electronic Structures of BN Supports by Doping Carbon” *ACS Catalysis* **12**, (2022): 14152-14161.

[13] Y. Li, M. Kottwitz, J. L. Vincent, M. J. Enright, Z. Liu, L. Zhang, J. Huang, S. D. Senanayake, W.-C. D. Yang, P. A. Crozier, R. G. Nuzzo, A. I. Frenkel, “Dynamic structure of active sites in ceria-supported Pt catalysts for the water gas shift reaction” *Nature Communications.* **12**, (2021): 914.

[14] Y. Chen, J. Lin, L. Li, B. Qiao, J. Liu, Y. Su, X. Wang, “Identifying Size Effects of Pt as Single Atoms and Nanoparticles Supported on FeOx for the Water-Gas Shift Reaction” *ACS Catalysis* **8**, (2018): 859-868.

[15] S. I. Venturini, E. Antolini, J. Perez, “Effect of CeO_2_ Presence on the Electronic Structure and the Activity for Ethanol Oxidation of Carbon Supported Pt” *Catalysts* **11**, (2021): 579.

[16] Z. Zhang, J. Tian, Y. Lu, S. Yang, D. Jiang, W. Huang, Y. Li, J. Hong, A. S. Hoffman, S. R. Bare, M. H. Engelhard, A. K. Datye, Y. Wang, “Memory-dictated dynamics of single-atom Pt on CeO2 for CO oxidation” *Nature Communications* **14**, (2023): 2664.

[17] J. L. Vincent, P. A. Crozier, “Atomic level fluxional behavior and activity of CeO_2_-supported Pt catalysts for CO oxidation” *Nature Communications.* **12**, (2021): 5789.

[18] J. Yu, X. Qin, Y. Yang, M. Lv, P. Yin, L. Wang, Z. Ren, B. Song, Q. Li, L. Zheng, S. Hong, X. Xing, D. Ma, M. Wei, X. Duan, “Highly Stable Pt/CeO_2_ Catalyst with Embedding Structure toward Water–Gas Shift Reaction” *Journal of the Americn Chemical Society* **146**, (2023): 1071-1080.

[19] S. Das, K. H. Lim, T. Z. H. Gani, S. Aksari, & S. Kawi, “Bi-functional CeO_2_ coated NiCo-MgAl core-shell catalyst with high activity and resistance to coke and H_2_S poisoning in methane dry reforming” *Applied Catalysis B: Environmental* **323**, (2023): 122141.

[20] J. Zheng, S. Impeng, J. Liu, J. Deng, & D. Zhang, “Mo promoting Ni-based catalysts confined by halloysite nanotubes for dry reforming of methane: Insight of coking and H_2_S poisoning resistance”. *Applied Catalysis B: Environmental* **342**, (2024): 123369.

[21] X. Chen, J. Jiang, F. Yan, K. Li, S. Tian, Y. Gao, H. Zhou, “Dry Reforming of Model Biogas on a Ni/SiO_2_ Catalyst: Overall Performance and Mechanisms of Sulfur Poisoning and Regeneration” *ACS Sustainable Chemistry Engineering* **5**, (2017): 10248-10257.

[22] V. Pawar, S. Appari, D. S. Monder, & V. M. Janardhanan, “Study of the Combined Deactivation Due to Sulfur Poisoning and Carbon Deposition during Biogas Dry Reforming on Supported Ni Catalyst” *Industrial & Engineering Chemistry Research* **56**, (2017): 8448-8455.

[23] D. Li, Q. Zhu, Z. Bao, L. Jin, & H. Hu, "New insight and countermeasure for sulfur poisoning on nickel-based catalysts during dry reforming of methane” *Fuel* **363**, (2024): 131045.
